# Supplementary material for: Molecular and developmental deficits in Smith-Magenis syndrome human stem cell-derived cortical neural models
Source: Am J Hum Genet. 2025 Aug 28;112(10):2338–62. doi: 10.1016/j.ajhg.2025.07.020 (PMC12696504; doi:10.1016/j.ajhg.2025.07.020)
Supplement: Document S1. Figures S1–S18 and Tables S1, S2, and S4 [file mmc1.pdf]

The American Journal of Human Genetics, Volume *112*

Supplemental information

Molecular and developmental deficits in Smith-Magenis syndrome human stem cell-derived cortical neural models

Yu-Ju Lee, Ya-Ting Chang, Yoobin Cho, Max Kowalczyk, Adrian Dragoiescu, Alain Pacis, Senthilkumar Kailasam, François Lefebvre, Qihuang Zhang, Xiaojing Gao, and Wei-Hsiang Huang

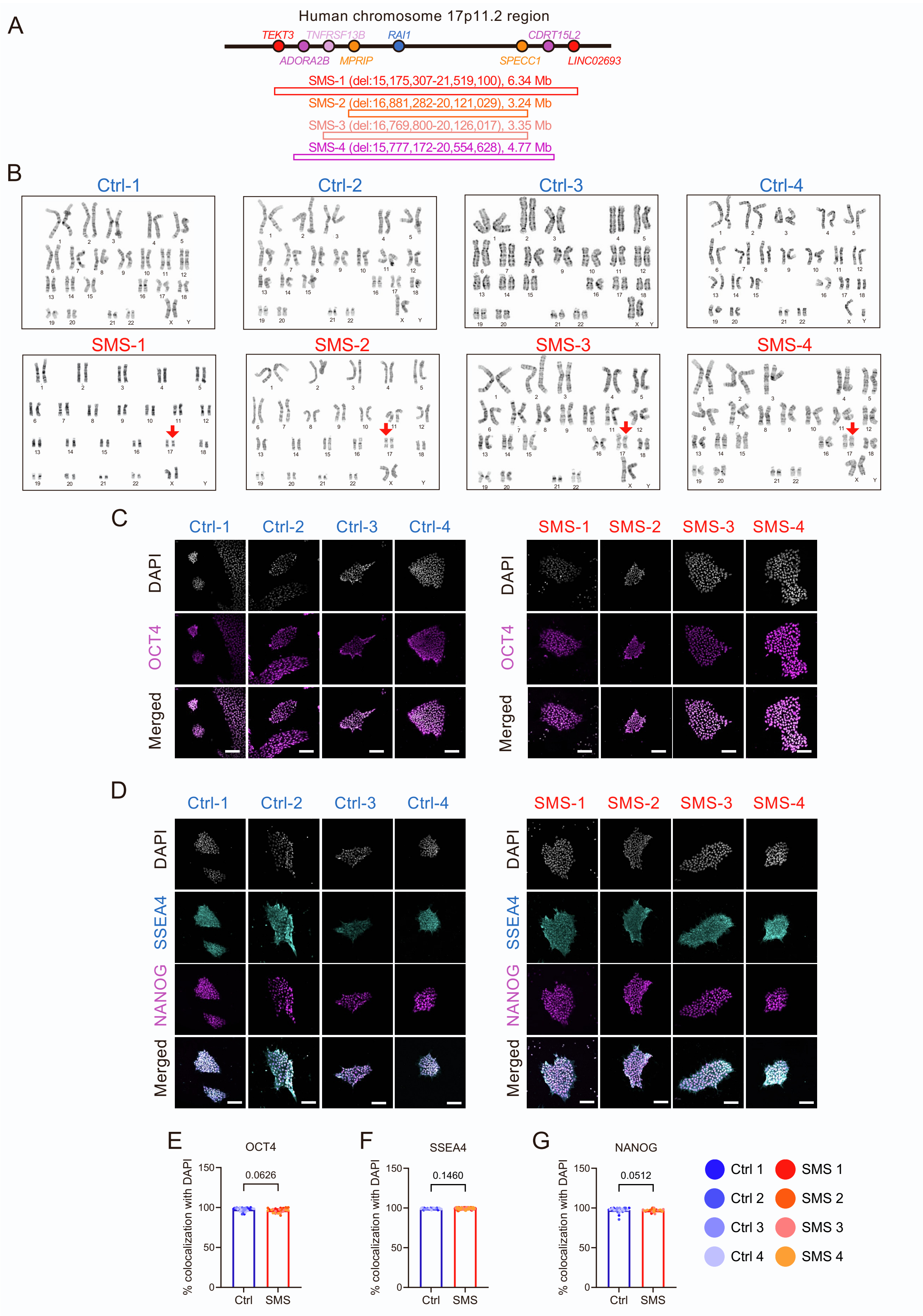
A
Human chromosome 17p11.2 region
TEKT3
TNFRSF13B
RAI1
CDRT15L2
ADORA2B
MPRIP
SPECC1
LINC02693
SMS-1 (del:15,175,307-21,519,100), 6.34 Mb
SMS-2 (del:16,881,282-20,121,029), 3.24 Mb
SMS-3 (del:16,769,800-20,126,017), 3.35 Mb
SMS-4 (del:15,777,172-20,554,628), 4.77 Mb
B
Ctrl-1
Ctrl-2
Ctrl-3
Ctrl-4
SMS-1
SMS-2
SMS-3
SMS-4
C
Ctrl-1 Ctrl-2 Ctrl-3 Ctrl-4
SMS-1 SMS-2 SMS-3 SMS-4
DAPI
OCT4
Merged
D
Ctrl-1 Ctrl-2 Ctrl-3 Ctrl-4
SMS-1 SMS-2 SMS-3 SMS-4
DAPI
SSEA4
NANOG
Merged
E
OCT4
0.0626
F
SSEA4
0.1460
G
NANOG
0.0512
% colocalization with DAPI
150
100
50
0
Ctrl
SMS
Ctrl 1
Ctrl 2
Ctrl 3
Ctrl 4
SMS 1
SMS 2
SMS 3
SMS 4

Figure S1: Characterization of Ctrl and SMS hiPSCs

(A) Schematic diagram showing del(17)p11.2 in four clinically diagnosed SMS patients, including the boundary genes deleted at both extremities of the CNV. From left to right: Tektin 3 (*TEKT3*); Adenosine A2b receptor (*ADORA2B*); TNF receptor superfamily member 13B (*TNFRSF13B*); Myosin phosphatase Rho interacting protein (*MPRIP*); Sperm antigen with calponin homology and coiled-coil domains 1 (*SPECC1*); CMT1A duplicated region transcript 15-like 2 (*CDRT15L2*); Long intergenic non-protein coding RNA 2693 (*LINC02693*). *RAI1* (in blue) is deleted in all four patients.

(B) Karyograms of hiPSCs showing normal chromosomes in all lines except del(17)p11.2 (red arrows) in SMS lines. Note that all samples (except Ctrl-4) were derived from female individuals.

(C) Representative images of Ctrl and SMS hiPSC colonies stained with a pluripotency marker OCT4 (magenta). DAPI-labeled nuclei are in grey. Scale bars: 100µm.

(D) Representative images of Ctrl and SMS hiPSC colonies stained with pluripotency markers SSEA4 (cyan) and NANOG (magenta). DAPI-labeled nuclei are in grey. Scale bars: 100µm.

(E) Quantification of OCT4 in Ctrl and SMS hiPSCs. 32 hiPSC colonies per genotype with 8 colonies per cell line. Each dot represents one colony. $U = 373$, p=0.0626.

(F) Quantification of SSEA4 in Ctrl and SMS hiPSCs. 32 hiPSC colonies per genotype with 8 colonies per cell line. Each dot represents one colony. $U = 405.5$, p=0.1460.

(G) Quantification of NANOG in Ctrl and SMS hiPSCs. 32 hiPSC colonies per genotype with 8 colonies per cell line. Each dot represents one colony. $U = 367$, p=0.0512.

Data presented as mean ± S.E.M.; (**E-G**) U- and p-values by two-tailed Mann-Whitney tests.

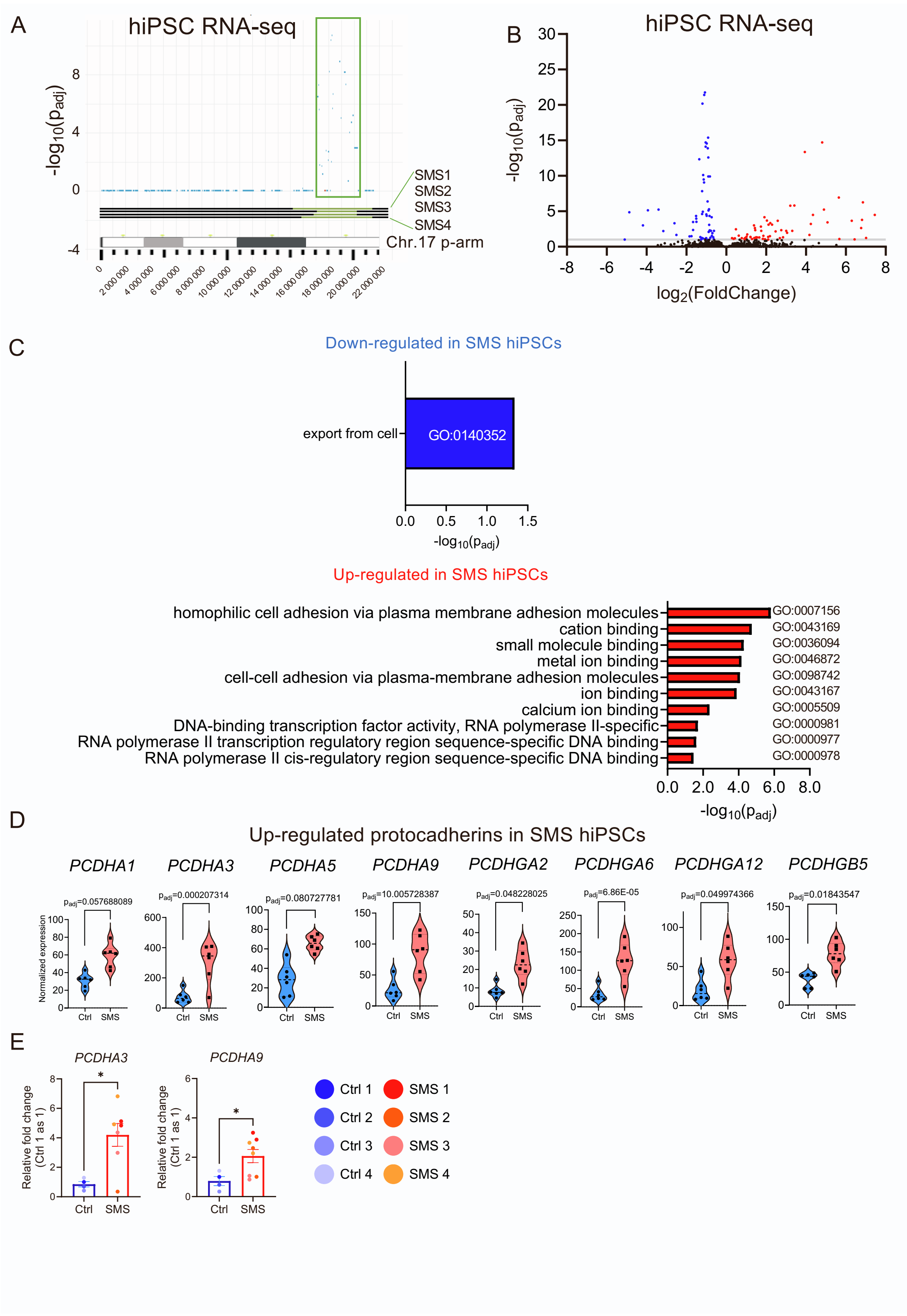
A
hiPSC RNA-seq
-log10(padj)
SMS1
SMS2
SMS3
SMS4
Chr.17 p-arm
B
hiPSC RNA-seq
-log10(padj)
log2(FoldChange)
C
Down-regulated in SMS hiPSCs
export from cell
GO:0140352
-log10(padj)
Up-regulated in SMS hiPSCs
homophilic cell adhesion via plasma membrane adhesion molecules GO:0007156
cation binding GO:0043169
small molecule binding GO:0036094
metal ion binding GO:0046872
cell-cell adhesion via plasma-membrane adhesion molecules GO:0098742
ion binding GO:0043167
calcium ion binding GO:0005509
DNA-binding transcription factor activity, RNA polymerase II-specific GO:0000981
RNA polymerase II transcription regulatory region sequence-specific DNA binding GO:0000977
RNA polymerase II cis-regulatory region sequence-specific DNA binding GO:0000978
-log10(padj)
D
Up-regulated protocadherins in SMS hiPSCs
PCDHA1 padj=0.057688089
PCDHA3 padj=0.000207314
PCDHA5 padj=0.080727781
PCDHA9 padj=10.005728387
PCDHGA2 padj=0.048228025
PCDHGA6 padj=6.86E-05
PCDHGA12 padj=0.049974366
PCDHGB5 padj=0.01843547
Normalized expression
Ctrl
SMS
E
PCDHA3
PCDHA9
Relative fold change (Ctrl 1 as 1)
Ctrl
SMS
Ctrl 1
Ctrl 2
Ctrl 3
Ctrl 4
SMS 1
SMS 2
SMS 3
SMS 4

Figure S2: RNA-sequencing showing dysregulation of genes encoding organismal development and cell adhesion molecules in SMS hiPSCs

(A) A Manhattan plot displaying the genomic landscape of significantly downregulated genes on the chromosome 17 p-arm in SMS hiPSCs (compared to Ctrl hiPSCs) based on $-\log_{10}$ transformed p_{adj} derived from a one-sided Wald test. The x-axis represents the genomic position, and the y-axis corresponds to the significance level of differential expression. The blue dots represent individual genes, with their positions on the x-axis representing their location on chromosome 17p and their heights on the y-axis indicating the significance of their differential expressions. The dark horizontal lines at the bottom indicate chromosomes, with the green segments corresponding to the regions subjected to del(17)p11.2 in SMS hiPSCs.

(B) A Volcano plot showing the global transcriptomic changes when comparing SMS with Ctrl hiPSCs. Each dot represents a gene. The $\log_2$ fold change of each gene is represented on the x-axis and the $-\log_{10}$ of its $\mathbf{p_{adj}}$ is on the y-axis. Up-regulated genes in SMS hiPSCs with p_{adj} less than 0.1 are indicated by red dots. Down-regulated genes in SMS hiPSCs with p_{adj} less than 0.1 are indicated by blue dots. The grey line indicates p_{adj}=0.1.

(C) GO analysis showing that cell export genes are down-regulated in SMS hiPSCs and cell adhesion molecules are up-regulated in SMS hiPSCs. The GO terms for down-regulated genes (in blue) and up-regulated genes (in red) and the respective $-\log_{10}(p_{adj})$ are shown.

(D) Violin plots illustrating that bulk RNA-seq detected up-regulated protocadherin genes in SMS hiPSCs when compared to Ctrls. Each dot represents a sample. p_{adj} by Wald test.

(E) Quantitative RT-PCR experiments confirming altered expression of *PCDHA3* (t=3.181, df=9, p=0.0112) and *PCDHA9* (t=2.487, df=10, p=0.0322) in SMS hiPSCs when compared to Ctrls (relative to Ctrl 1). t-ratios, df, and p-values by two-tailed unpaired t-tests.

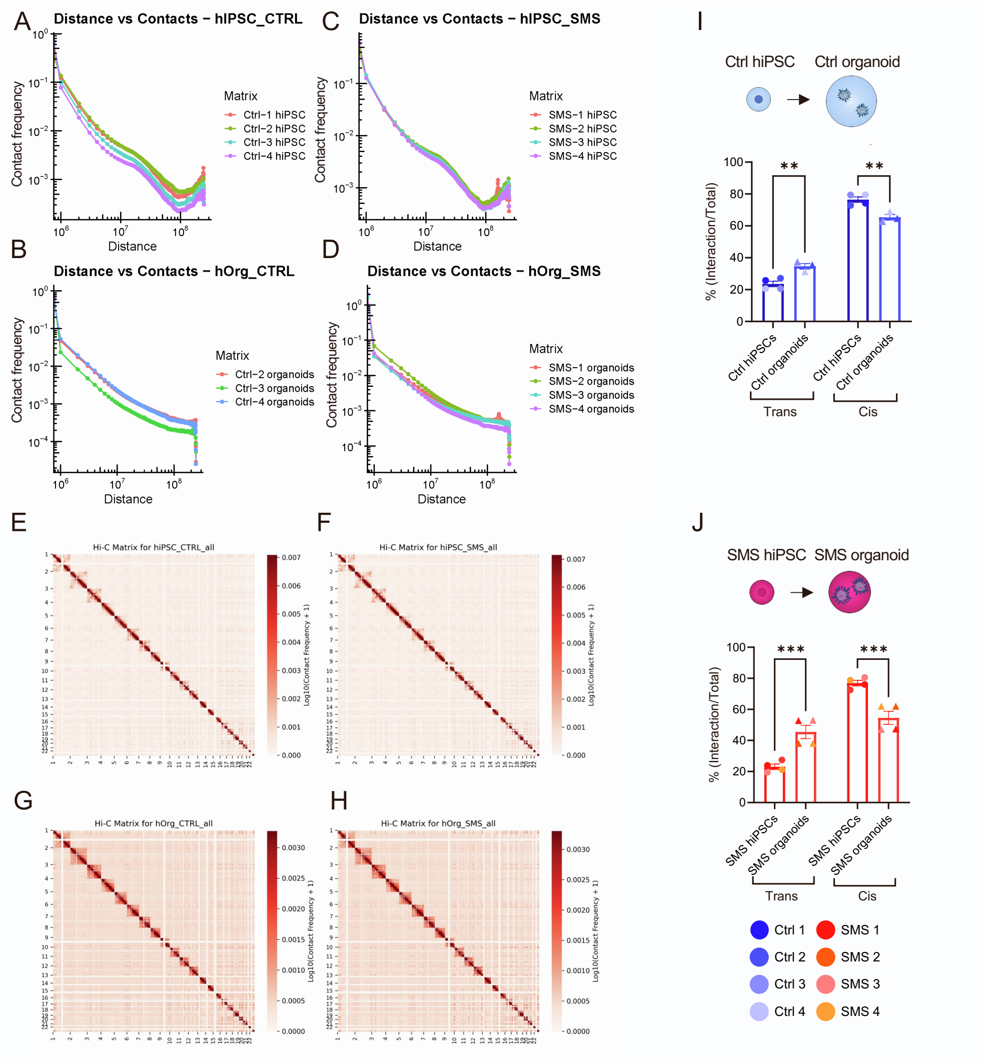

Figure S3: Quality control of Hi-C data obtained from Ctrl and SMS hiPSCs and cortical organoid models at day 75 differentiation

(A-D) Plots showing interaction frequency decay as a function of the genomic distance. Normalized Hi-C data binned at 250 kb were converted to contact frequency datasets. The X-axis indicates distance and the Y-axis indicates contact frequencies (both in $\log_{10}$ scales). Shown are the decay plots for Ctrl hiPSC (**A**), Ctrl cortical organoids (**B**), SMS hiPSC (**C**), and SMS cortical organoids (**D**). Note that the Ctrl-1 organoid library was removed due to insufficient data quality.

(E-H) Interchromosomal contact maps in Ctrl hiPSCs (1-4 combined, **E**), SMS hiPSCs (1-4 combined, **F**), Ctrl cortical organoids (2-4 combined, **G**), and SMS cortical organoids (1-4 combined, **H**).

(I-J) Percent distribution of trans- and cis-chromosomal interactions during in vitro corticogenesis from hiPSCs to organoids for Ctrl (**I**, $F(1,10)=38.8$, $p=0.0027$) and SMS (**J**, $F(1,12)=47.19$, $p=0.0008$) samples. F(DFn, DFd) and *p*-values were calculated by two-way ANOVA with post hoc Šídák multiple comparisons. Ctrl and SMS organoids showed increased trans-interactions and decreased cis-interactions compared to corresponding hiPSCs. Circles represent hiPSCs and triangles represent organoids.

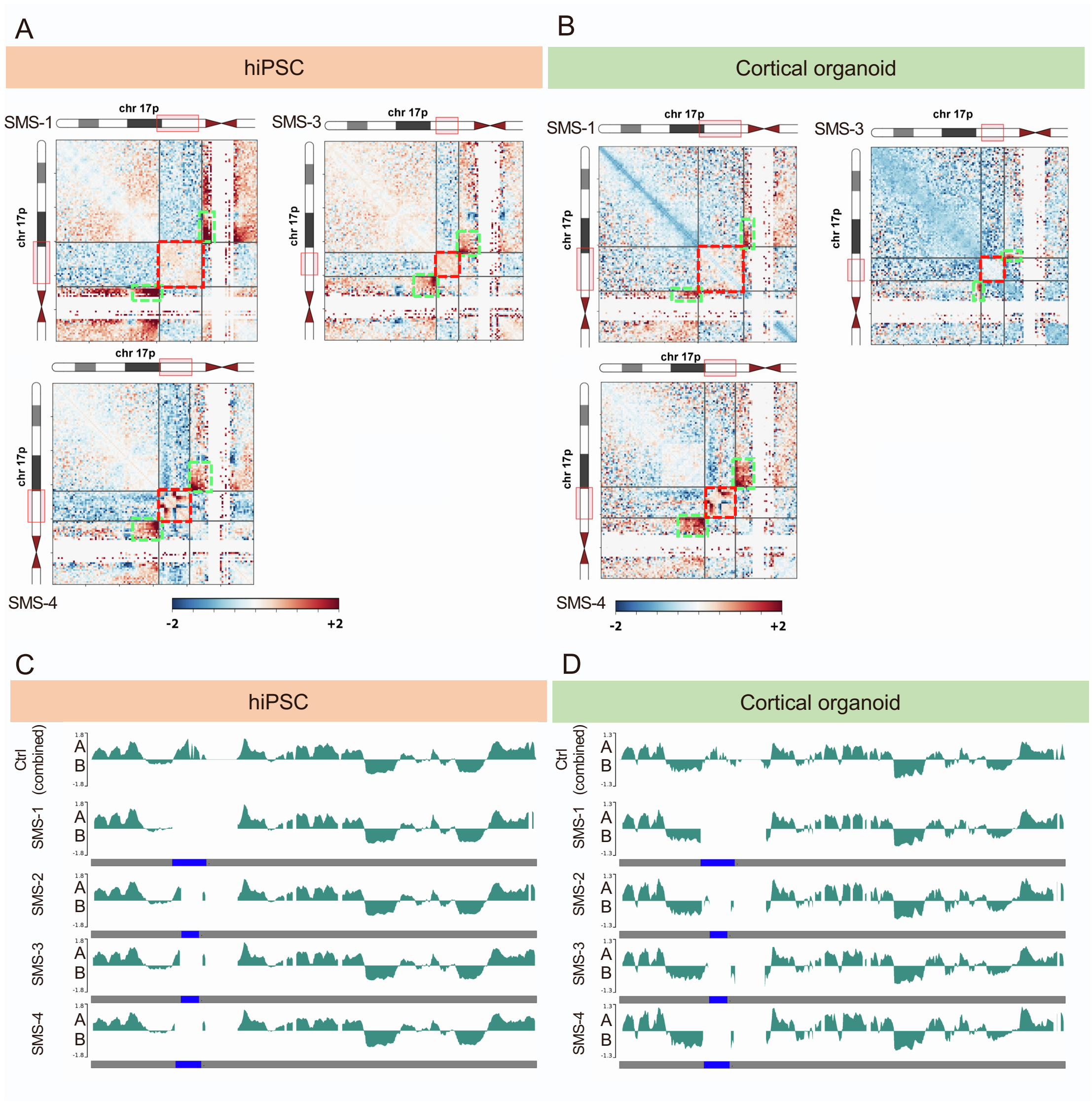

Figure S4: Hi-C data obtained from Ctrl and SMS hiPSCs and cortical organoid models at day 75 differentiation

(A-B) Heatmaps showing increased cis-contacts within del(17)p11.2 deletion boundaries (red dashed boxes) and between the DNA regions flanking del(17)p11.2 (green dashed boxes) in SMS hiPSCs (**A**) and SMS cortical organoids (**B**) compared to corresponding Ctrl samples.

(C-D) A/B compartments identified on chromosome 17 in Ctrl hiPSCs (1-4 combined) and SMS 1-4 hiPSC (**C**) and in Ctrl cortical organoids (2-4 combined) and SMS 1-4 cortical organoids (**D**). Del(17)p11.2 regions in each SMS line are indicated by blue boxes at the bottom. No A/B switching was observed in SMS lines.

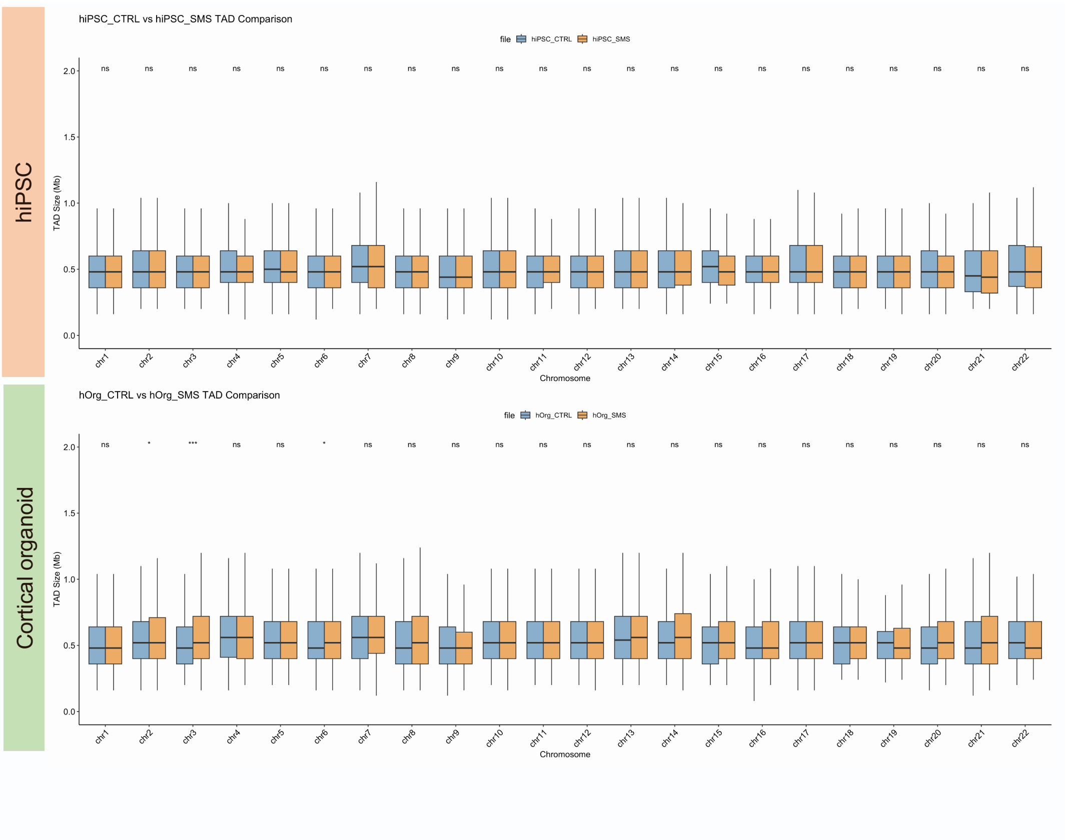

Figure S5: Comparison of TAD sizes in Ctrl and SMS hiPSCs and cortical organoid models at day 75 differentiation

Averaged TAD size (Mb) in Ctrl hiPSCs (1-4 combined) versus SMS hiPSCs (1-4 combined) (top) and Ctrl cortical organoids (2-4 combined) versus SMS cortical organoids (1-4 combined) (bottom). At this scale, no significant differences in TAD sizes were observed in most pairs of autosomes (ns= p >0.05, two-tailed t-tests).

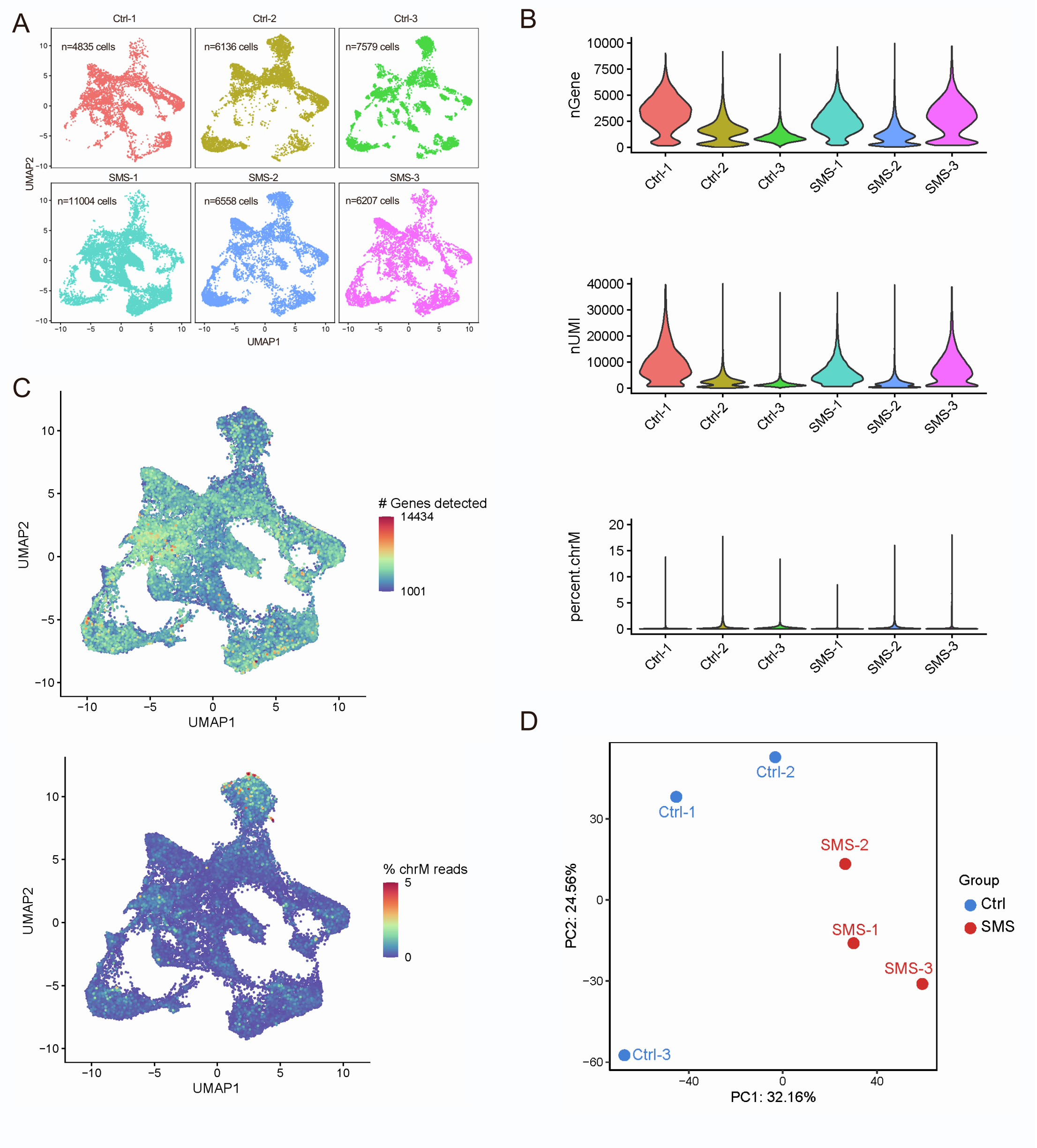

Figure S6: Quality control of snRNA-seq data from Ctrl and SMS hiPSCs-derived cortical organoids at day 75 differentiation

(A-D) Quality control of snRNA-seq data, including (**A**), the number of cells harvested from each line (**B**), the number of detected genes (top), the number of unique molecular identifiers (UMIs) (middle), and the percentage of UMIs assigned to mitochondrial genes (bottom) per cell for each line. **(C)** UMAP plot of all cells color-coded by number of genes detected and the proportion of mitochondrial reads. **(D)** PCA analysis showing that the pseudo-bulked transcriptomes of Ctrl-1-3 cortical organoids were separated from SMS-1-3 cortical organoids.

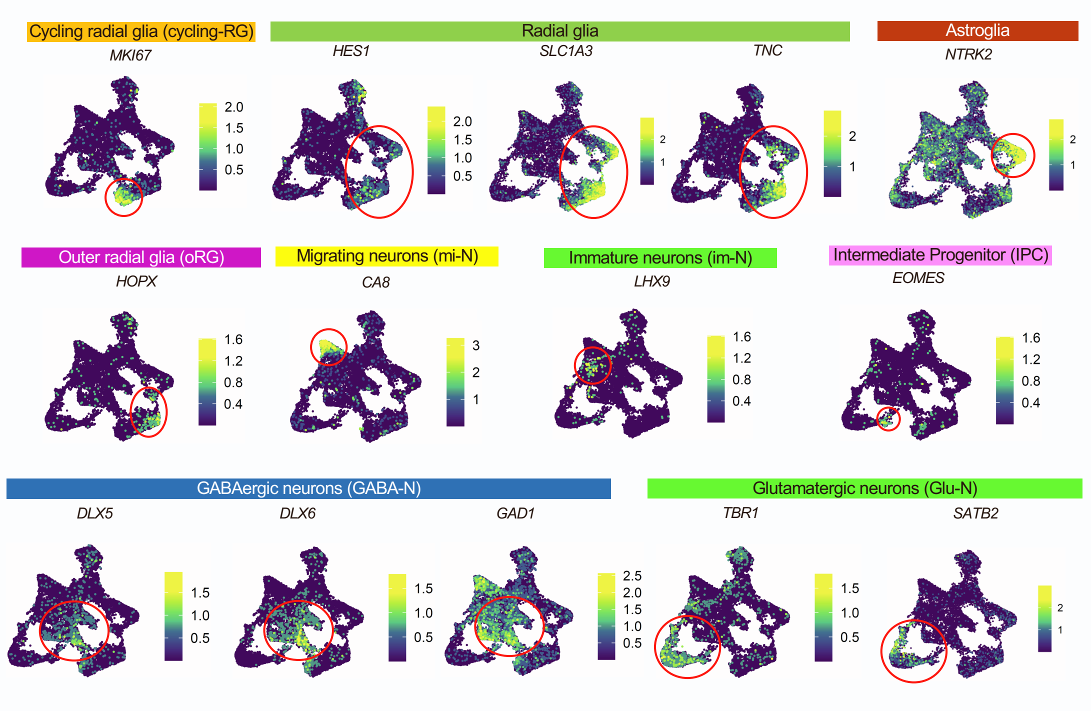

Figure S7: Cell type classification using snRNA-seq data from Ctrl and SMS hiPSCs-derived cortical organoids at day 75 differentiation

UMAP visualization of all cell clusters for additional markers indicated with red circles. *MKI67* shows actively dividing cycling-RGs, radial glial progenitors are marked by *HES1*, *SLC1A3*, and *TNC*, astroglia expresses high levels of *NTRK2*, outer radial glia (oRG) is labelled by *HOPX*, *CA8* expression is enriched in migrating neurons (mi-N), *LHX9* is expressed in immature neurons (im-N), intermediate progenitor cells (IPC) express *EOMES*, multiple GABAergic neuronal groups (GABA-N) express *DLX5*, *DLX6*, and *GAD1*, and glutamatergic neurons (Glu-N) are defined by *TBR1* and *SATB2* expression. Note that *TBR1* and *GAD1* are also expressed in some tRG, immature neurons, and migrating neurons that likely become Glu-N or GABA-N.

A

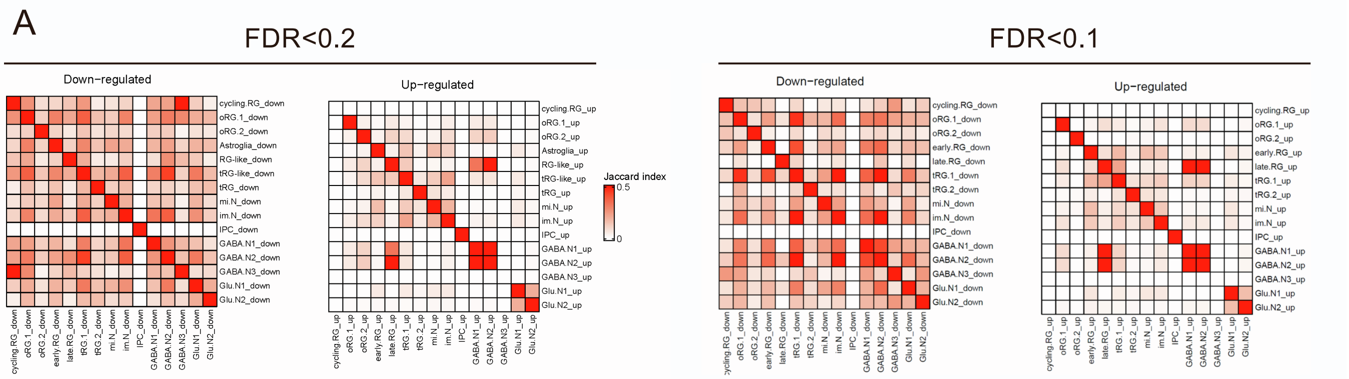

B

DEG number	Cycling-RG	Early RG	GABA-N1	GABA-N2	GAB A-N3	Glu-N1	Glu-N2	im-N	IPC	Late-RG	mi-N	oRG-1	oRG-2	tRG-1	tR G-2
FDR<0.2	8	77	37	19	7	128	96	72	5	10	149	47	48	48	187
FDR<0.1	4	42	26	15	4	84	51	46	3	5	93	37	24	30	90

C

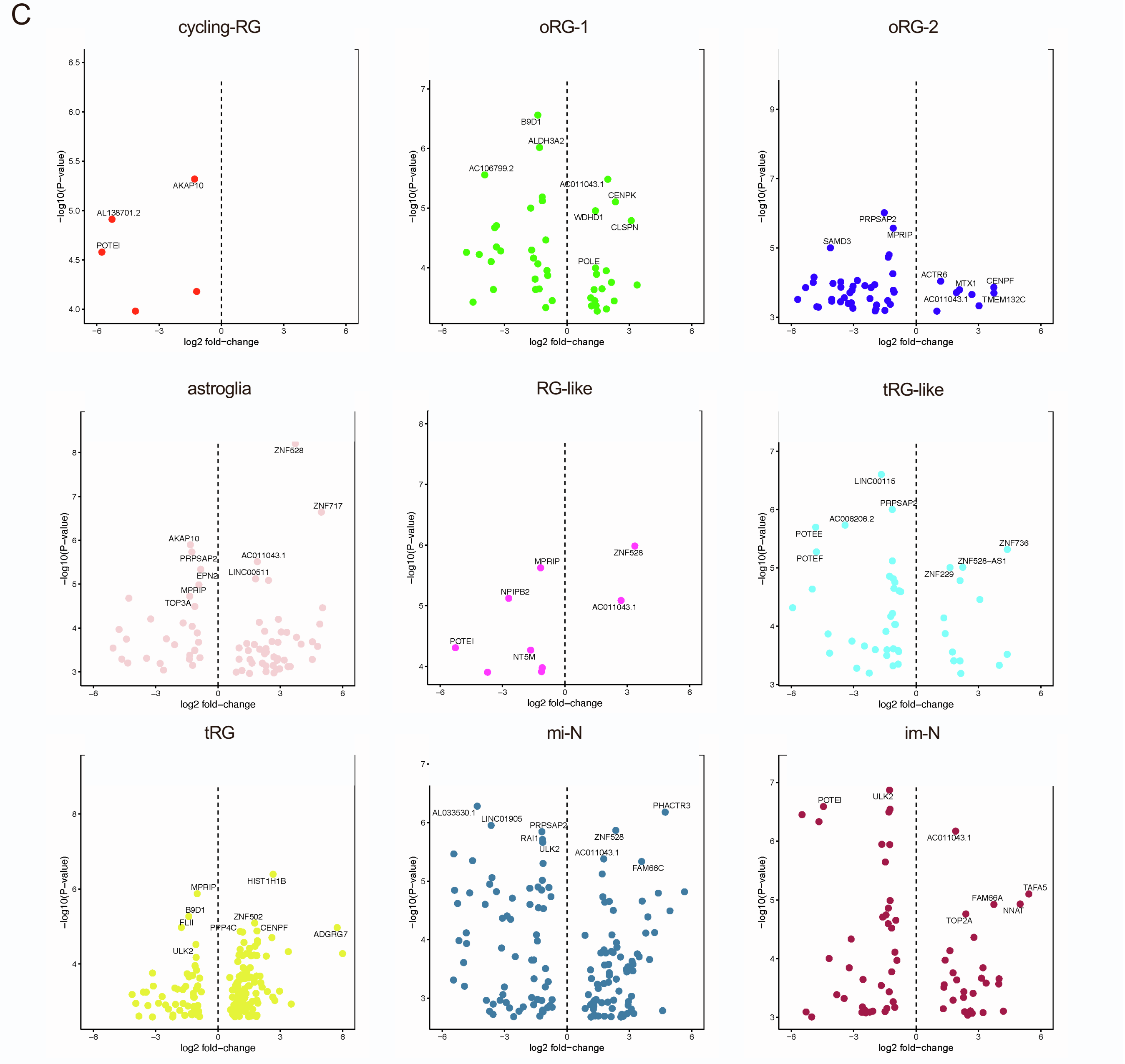

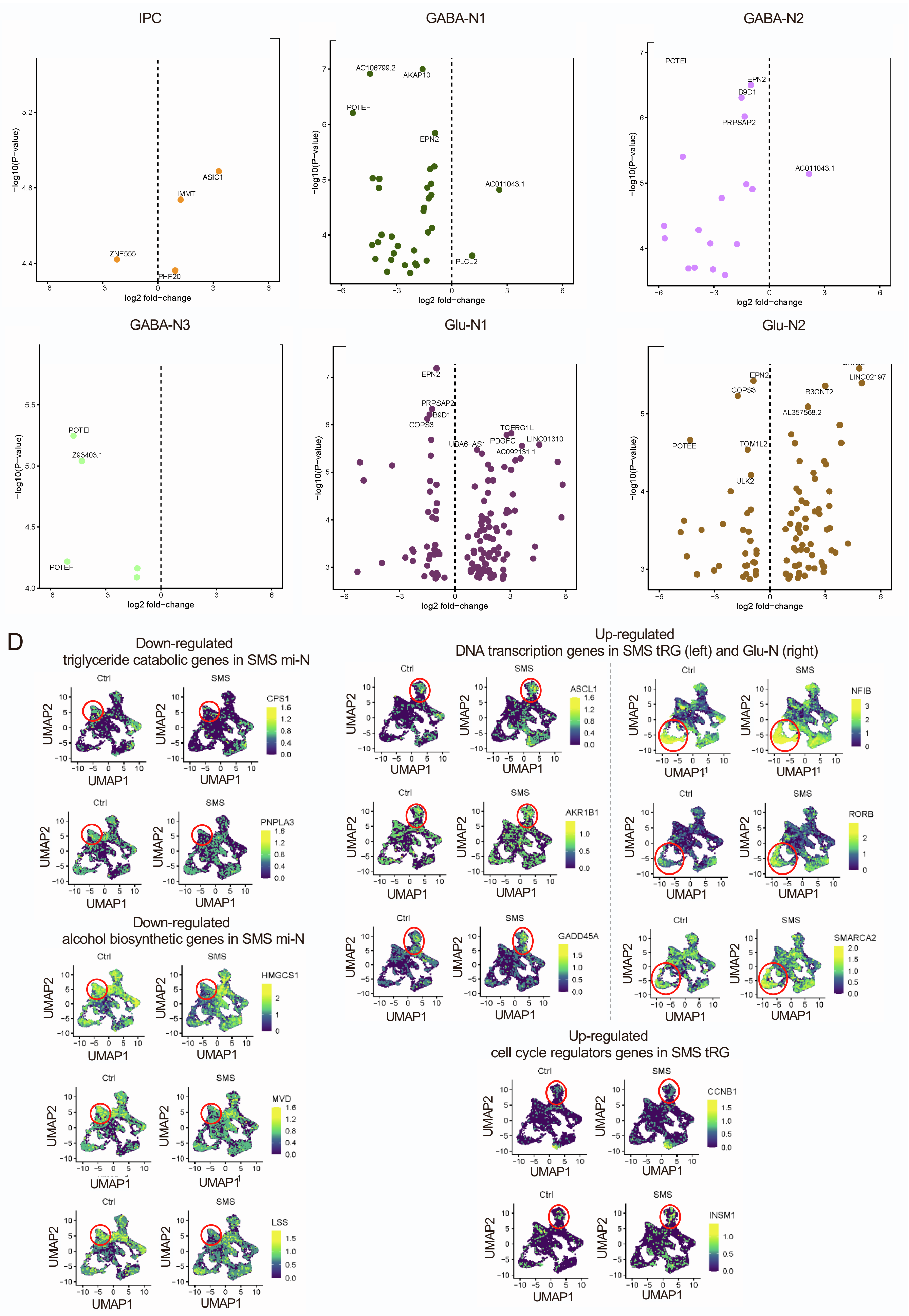

IPC
GABA-N1
GABA-N2
GABA-N3
Glu-N1
Glu-N2
−log10(P−value)
log2 fold−change
ASIC1
IMMT
ZNF555
PHF20
AC106799.2
AKAP10
POTEF
EPN2
AC011043.1
PLCL2
POTEI
B9D1
PRPSAP2
Z93403.1
COPS3
TCERG1L
UBA6−AS1
PDGFC
LINC01310
AC092131.1
LINC02197
B3GNT2
AL357568.2
POTEE
TOM1L2
ULK2
D
Down-regulated
triglyceride catabolic genes in SMS mi-N
Ctrl
SMS
UMAP1
UMAP2
CPS1
PNPLA3
Down-regulated
alcohol biosynthetic genes in SMS mi-N
HMGCS1
MVD
LSS
Up-regulated
DNA transcription genes in SMS tRG (left) and Glu-N (right)
ASCL1
AKR1B1
GADD45A
NFIB
RORB
SMARCA2
Up-regulated
cell cycle regulators genes in SMS tRG
CCNB1
INSM1

Figure S8: Differential gene expression analysis of snRNA-seq data from Ctrl and SMS hiPSCs-derived cortical organoids at day 75 differentiation

(A) Heatmaps showing similarity levels (Jaccard similarity index) of up-regulated and down-regulated genes between different cell clusters in cortical organoids, even after removing the del(17)p11.2 genes. A Jaccard similarity coefficient of 0 indicates no overlap and 1 indicates complete overlap. Left panels: FDR<0.2; Right panels: FDR<0.1.

(B) The number of DEGs in each cell cluster using FDR <0.2 or FDR<0.1.

(C) Volcano plots showing DEGs in each cell cluster ($p_{adj} < 0.2$). The representations are as follows: x-axis, $\log_2$ fold-change; y-axis, $-\log_{10}$ of a *p*-value.

(D) UMAP visualization of down-regulated catabolic genes in SMS mi-N (upper left), down-regulated alcohol biosynthetic genes in SMS mi-N (lower left), up-regulated DNA transcription genes in SMS tRG and Glu-N (upper right), and up-regulated cell cycle regulator genes in SMS tRG (lower right).

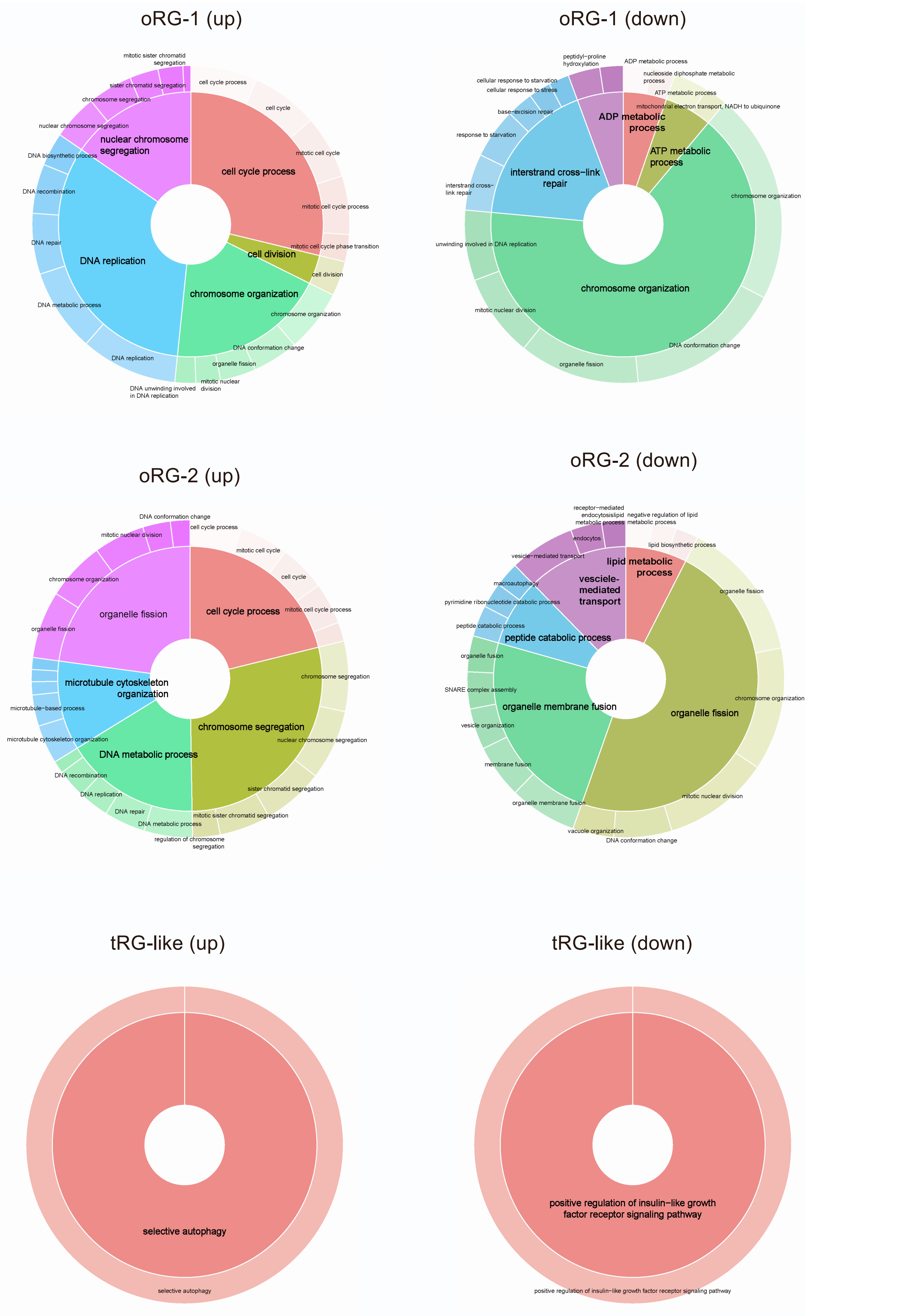

oRG-1 (up)
mitotic sister chromatid segregation
sister chromatid segregation
chromosome segregation
nuclear chromosome segregation
DNA biosynthetic process
DNA recombination
DNA repair
DNA metabolic process
DNA replication
DNA unwinding involved in DNA replication
mitotic nuclear division
organelle fission
DNA conformation change
chromosome organization
cell division
mitotic cell cycle phase transition
mitotic cell cycle process
mitotic cell cycle
cell cycle
cell cycle process
nuclear chromosome segregation
DNA replication
chromosome organization
cell division
cell cycle process
oRG-1 (down)
peptidyl−proline hydroxylation
ADP metabolic process
nucleoside diphosphate metabolic process
ATP metabolic process
mitochondrial electron transport, NADH to ubiquinone
cellular response to starvation
cellular response to stress
base−excision repair
response to starvation
interstrand cross−link repair
unwinding involved in DNA replication
mitotic nuclear division
organelle fission
DNA conformation change
chromosome organization
ADP metabolic process
ATP metabolic process
interstrand cross−link repair
chromosome organization
oRG-2 (up)
DNA conformation change
mitotic nuclear division
cell cycle process
mitotic cell cycle
cell cycle
mitotic cell cycle process
chromosome organization
organelle fission
microtubule−based process
microtubule cytoskeleton organization
DNA recombination
DNA replication
DNA repair
DNA metabolic process
regulation of chromosome segregation
mitotic sister chromatid segregation
sister chromatid segregation
nuclear chromosome segregation
chromosome segregation
organelle fission
cell cycle process
microtubule cytoskeleton organization
DNA metabolic process
chromosome segregation
oRG-2 (down)
receptor−mediated endocytosis
lipid metabolic process
negative regulation of lipid metabolic process
endocytosis
lipid biosynthetic process
vesicle−mediated transport
macroautophagy
pyrimidine ribonucleotide catabolic process
peptide catabolic process
organelle fusion
SNARE complex assembly
vesicle organization
membrane fusion
organelle membrane fusion
vacuole organization
DNA conformation change
mitotic nuclear division
chromosome organization
organelle fission
lipid metabolic process
vesicle-mediated transport
peptide catabolic process
organelle membrane fusion
organelle fission
tRG-like (up)
selective autophagy
selective autophagy
tRG-like (down)
positive regulation of insulin−like growth factor receptor signaling pathway
positive regulation of insulin−like growth factor receptor signaling pathway

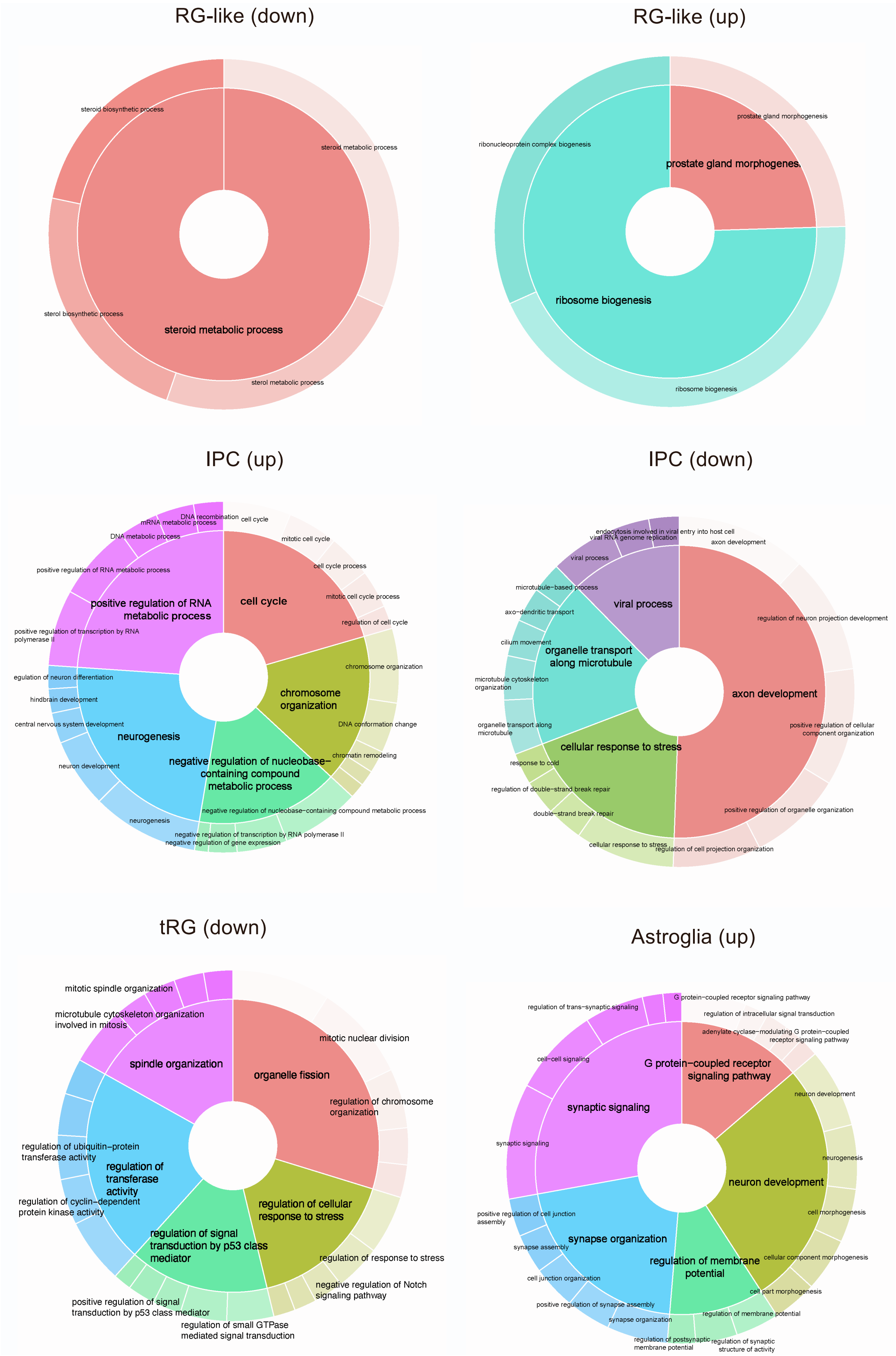
RG-like (down)
steroid biosynthetic process
steroid metabolic process
sterol biosynthetic process
steroid metabolic process
sterol metabolic process
RG-like (up)
prostate gland morphogenesis
ribonucleoprotein complex biogenesis
prostate gland morphogenes
ribosome biogenesis
ribosome biogenesis
IPC (up)
DNA recombination
mRNA metabolic process
cell cycle
DNA metabolic process
mitotic cell cycle
cell cycle process
positive regulation of RNA metabolic process
mitotic cell cycle process
regulation of cell cycle
positive regulation of RNA metabolic process
cell cycle
positive regulation of transcription by RNA polymerase II
chromosome organization
egulation of neuron differentiation
chromosome organization
hindbrain development
central nervous system development
DNA conformation change
neurogenesis
chromatin remodeling
neuron development
negative regulation of nucleobase−containing compound metabolic process
negative regulation of nucleobase−containing compound metabolic process
neurogenesis
negative regulation of transcription by RNA polymerase II
negative regulation of gene expression
IPC (down)
endocytosis involved in viral entry into host cell
viral RNA genome replication
axon development
viral process
microtubule−based process
viral process
axo−dendritic transport
regulation of neuron projection development
cilium movement
organelle transport along microtubule
microtubule cytoskeleton organization
axon development
organelle transport along microtubule
positive regulation of cellular component organization
cellular response to stress
response to cold
regulation of double−strand break repair
double−strand break repair
positive regulation of organelle organization
cellular response to stress
regulation of cell projection organization
tRG (down)
mitotic spindle organization
microtubule cytoskeleton organization involved in mitosis
mitotic nuclear division
spindle organization
organelle fission
regulation of chromosome organization
regulation of ubiquitin−protein transferase activity
regulation of transferase activity
regulation of cyclin−dependent protein kinase activity
regulation of cellular response to stress
regulation of signal transduction by p53 class mediator
regulation of response to stress
negative regulation of Notch signaling pathway
positive regulation of signal transduction by p53 class mediator
regulation of small GTPase mediated signal transduction
Astroglia (up)
G protein−coupled receptor signaling pathway
regulation of trans−synaptic signaling
regulation of intracellular signal transduction
adenylate cyclase−modulating G protein−coupled receptor signaling pathway
cell−cell signaling
G protein−coupled receptor signaling pathway
synaptic signaling
neuron development
synaptic signaling
neurogenesis
neuron development
cell morphogenesis
positive regulation of cell junction assembly
synapse organization
synapse assembly
cellular component morphogenesis
regulation of membrane potential
cell junction organization
cell part morphogenesis
positive regulation of synapse assembly
regulation of membrane potential
synapse organization
regulation of postsynaptic membrane potential
regulation of synaptic structure of activity

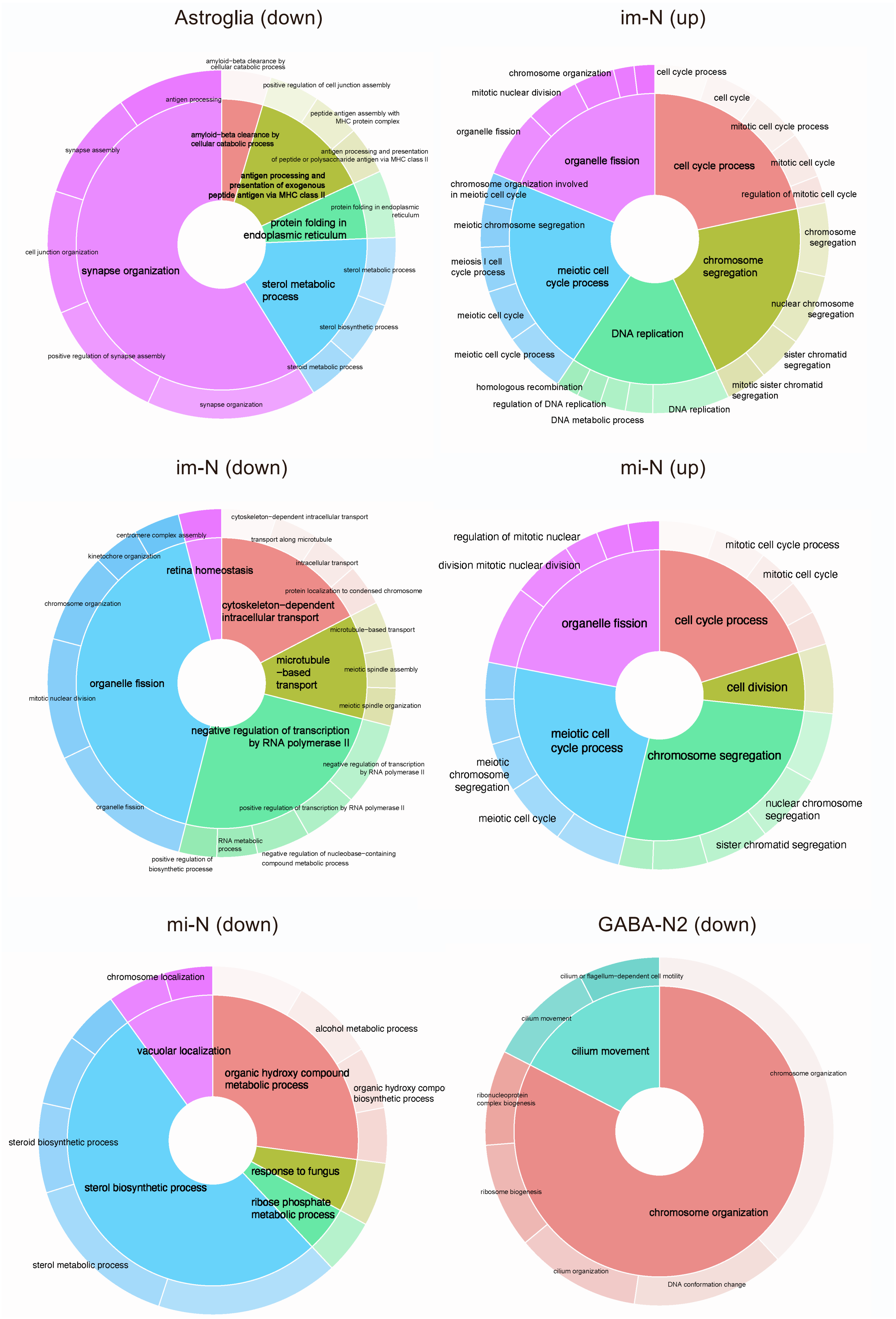
Astroglia (down)
amyloid−beta clearance by cellular catabolic process
positive regulation of cell junction assembly
peptide antigen assembly with MHC protein complex
antigen processing and presentation of peptide or polysaccharide antigen via MHC class II
protein folding in endoplasmic reticulum
sterol metabolic process
sterol biosynthetic process
steroid metabolic process
synapse organization
positive regulation of synapse assembly
cell junction organization
synapse assembly
antigen processing
amyloid−beta clearance by cellular catabolic process
antigen processing and presentation of exogenous peptide antigen via MHC class II
protein folding in endoplasmic reticulum
sterol metabolic process
synapse organization
im-N (up)
cell cycle process
cell cycle
mitotic cell cycle process
mitotic cell cycle
regulation of mitotic cell cycle
chromosome segregation
nuclear chromosome segregation
sister chromatid segregation
mitotic sister chromatid segregation
DNA replication
DNA metabolic process
regulation of DNA replication
homologous recombination
meiotic cell cycle process
meiotic cell cycle
meiosis I cell cycle process
meiotic chromosome segregation
chromosome organization involved in meiotic cell cycle
organelle fission
mitotic nuclear division
chromosome organization
organelle fission
cell cycle process
chromosome segregation
DNA replication
meiotic cell cycle process
im-N (down)
cytoskeleton−dependent intracellular transport
transport along microtubule
intracellular transport
protein localization to condensed chromosome
microtubule−based transport
meiotic spindle assembly
meiotic spindle organization
negative regulation of transcription by RNA polymerase II
positive regulation of transcription by RNA polymerase II
negative regulation of nucleobase−containing compound metabolic process
RNA metabolic process
positive regulation of biosynthetic processe
organelle fission
mitotic nuclear division
chromosome organization
kinetochore organization
centromere complex assembly
retina homeostasis
cytoskeleton−dependent intracellular transport
microtubule −based transport
negative regulation of transcription by RNA polymerase II
organelle fission
mi-N (up)
mitotic cell cycle process
mitotic cell cycle
nuclear chromosome segregation
sister chromatid segregation
meiotic cell cycle
meiotic chromosome segregation
regulation of mitotic nuclear
division mitotic nuclear division
organelle fission
cell cycle process
cell division
chromosome segregation
meiotic cell cycle process
mi-N (down)
chromosome localization
alcohol metabolic process
organic hydroxy compo biosynthetic process
steroid biosynthetic process
sterol metabolic process
vacuolar localization
organic hydroxy compound metabolic process
response to fungus
ribose phosphate metabolic process
sterol biosynthetic process
GABA-N2 (down)
cilium or flagellum−dependent cell motility
cilium movement
chromosome organization
ribonucleoprotein complex biogenesis
ribosome biogenesis
cilium organization
DNA conformation change
cilium movement
chromosome organization

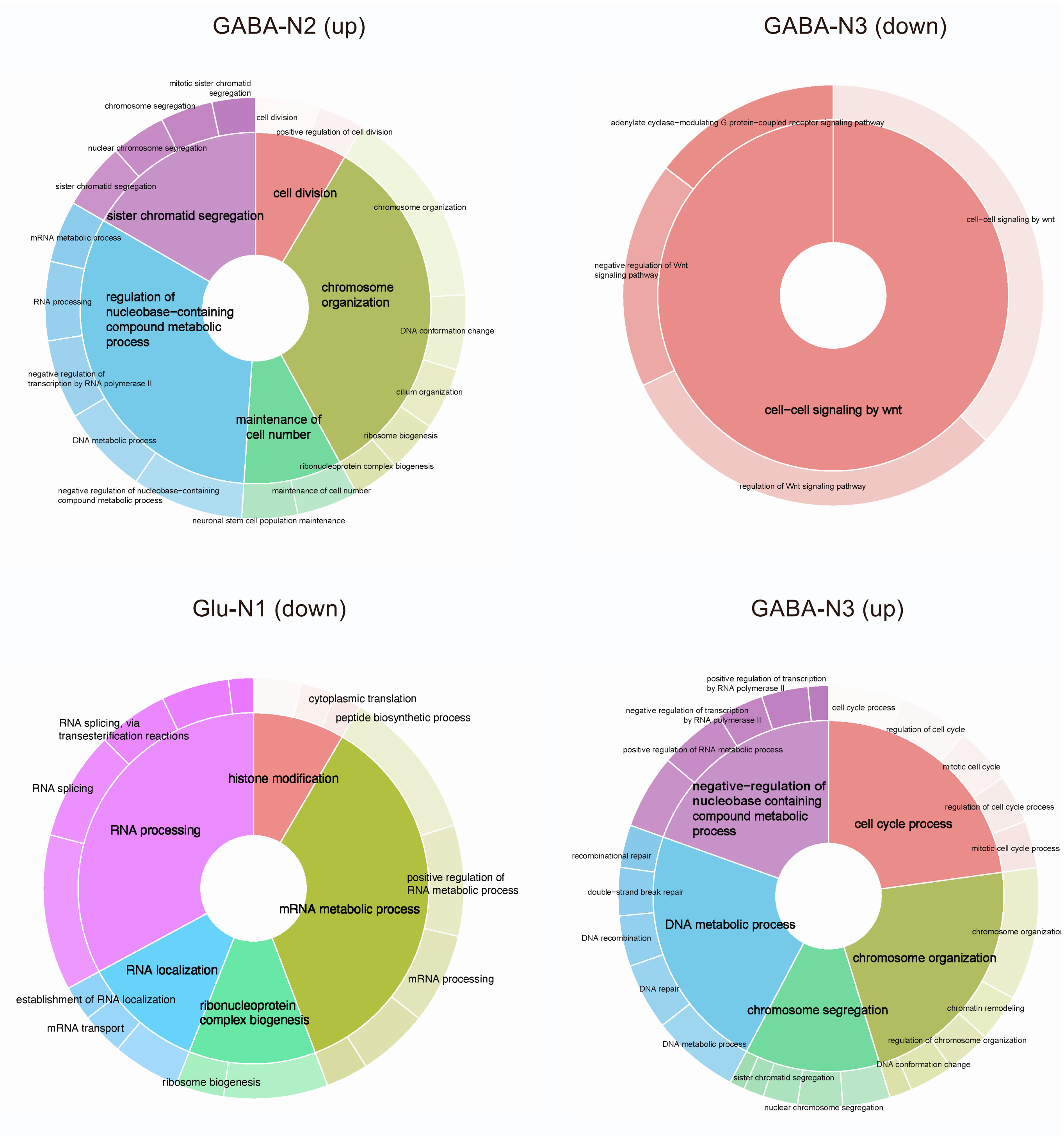

Figure S9: Gene set enrichment analysis (GSEA) and functional enrichment of DEGs in SMS hiPSCs-derived cortical organoids at day 75 differentiation

Hierarchical pie charts showing GO biological processes enriched among up- or down-regulated genes in different SMS cortical organoid cell clusters.

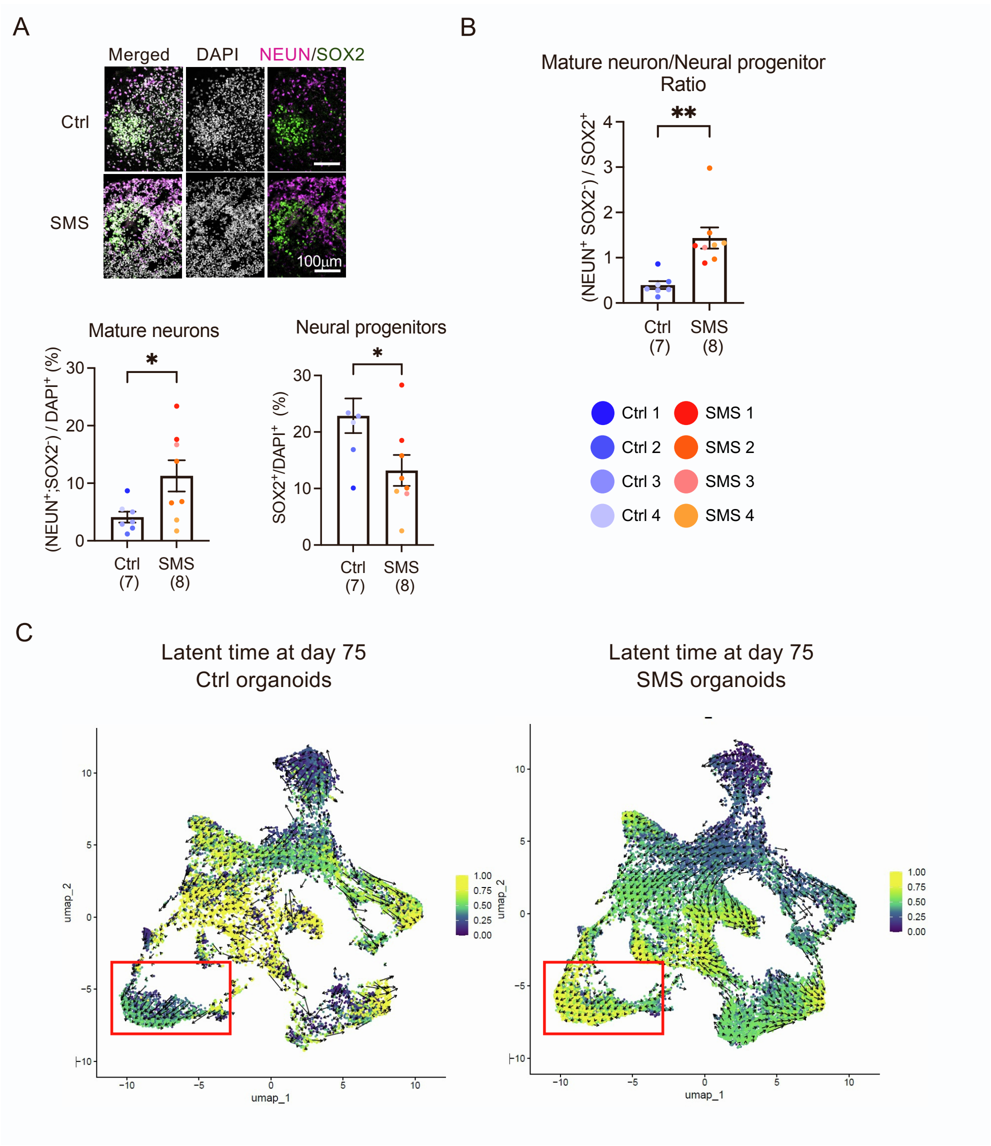

Figure S10: SMS hiPSCs-derived cortical organoids showed an accelerated neuronal maturation

(A-B) Top: Representative images of day 75 Ctrl and SMS organoids with SOX2 and NEUN co-staining. Bottom: SMS organoids showed an increased NEUN$^+$ SOX2$^-$ mature neuronal population, a decreased SOX2$^+$ neural progenitor population **(A)**, and an increased NEUN-to-SOX2 ratio **(B)** at day 75 of differentiation. Data were analyzed from 4um-thick confocal images of >6000 cells from each line (Ctrl n= 7 organoids, 16 images, 63557 cells; SMS n=8 organoids, 12 images, 40744 cells). $^*p<0.05$, $^{**}p<0.01$, *p*-values by Welch's t-tests.

(C) Latent time calculated using the snRNA-seq dataset revealed that SMS Glu-N1 and Glu-N2 populations (red rectangles) undergo accelerated neuronal maturation. By contrast, Ctrl cells are more biased towards the migrating neuron and immature neuronal trajectories than SMS cells. Heatmap: a latent time value of 0 represents the start of a cell's differentiation process, while a latent time value of 1 represents a terminal or final state.

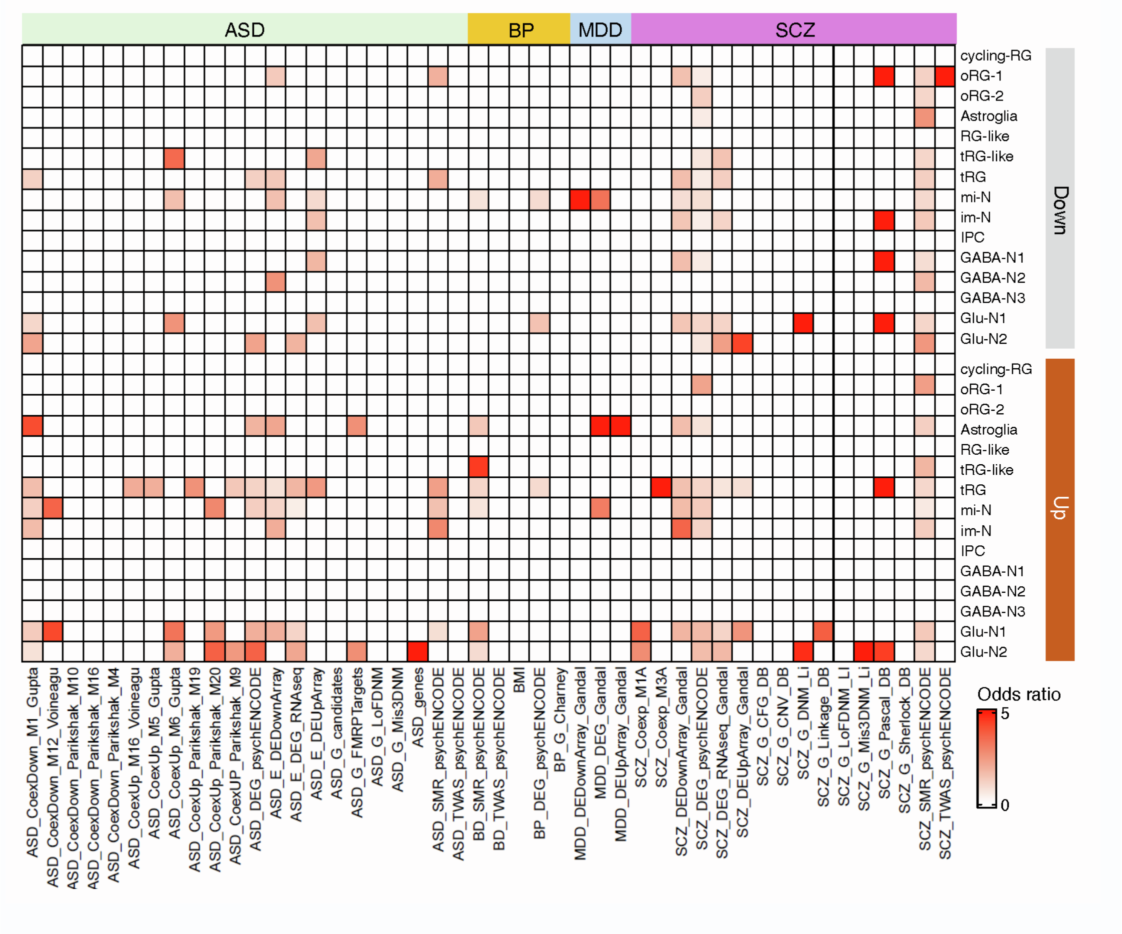

Figure S11: ASD and neuropsychiatric gene enrichment analysis using SMS DEGs (FDR<0.1)
Heatmap showing over-representation of autism spectrum disorder (ASD), bipolar disorder (BD), major depressive disorder (MDD) and schizophrenia (SCZ)-related genes in different cell cluster DEGs (FDR<0.1). The color of the box shows the odds ratio for enrichment. The odds ratios were calculated by Fisher's exact test.

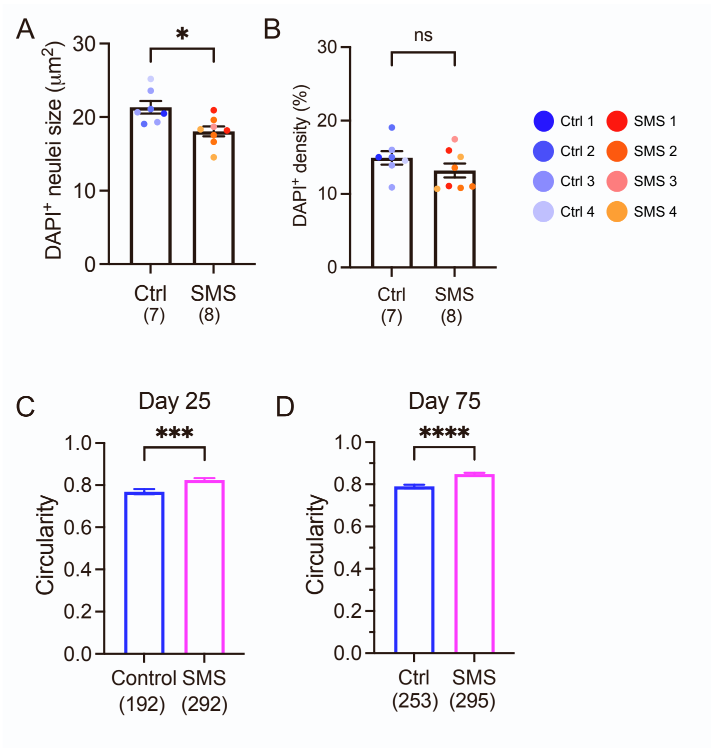

Figure S12: Cellular characterization of SMS cortical organoids

(A-B) SMS organoids exhibited a significantly decreased DAPI$^+$ nuclear size **(A)**, without measurable changes in DAPI$^+$ nuclear density **(B)** at day 75 of differentiation. Data were analyzed from 4μm-thick, confocal images of >40,000 cells from each genotype (Ctrl n= 7 orgnaoids; SMS n=8 orgnaoids). DAPI$^+$ density was measured by the percentage of DAPI$^+$ area within the orgnoid's surface area (excluding the ventricles).

(C-D) Measuring the circularity found that SMS organoids showed a small but significant increase in the C value at both day 25 **(C)** and day 75 **(D)** of differentiation when compared to age-matched Ctrl organoids. Shown are mean±SEM. The number of organoids in each group are listed below.

ns, not statistically different; *p<0.05, ***p<0.001, ****p<0.001, *p*-values by Welch's t-tests.

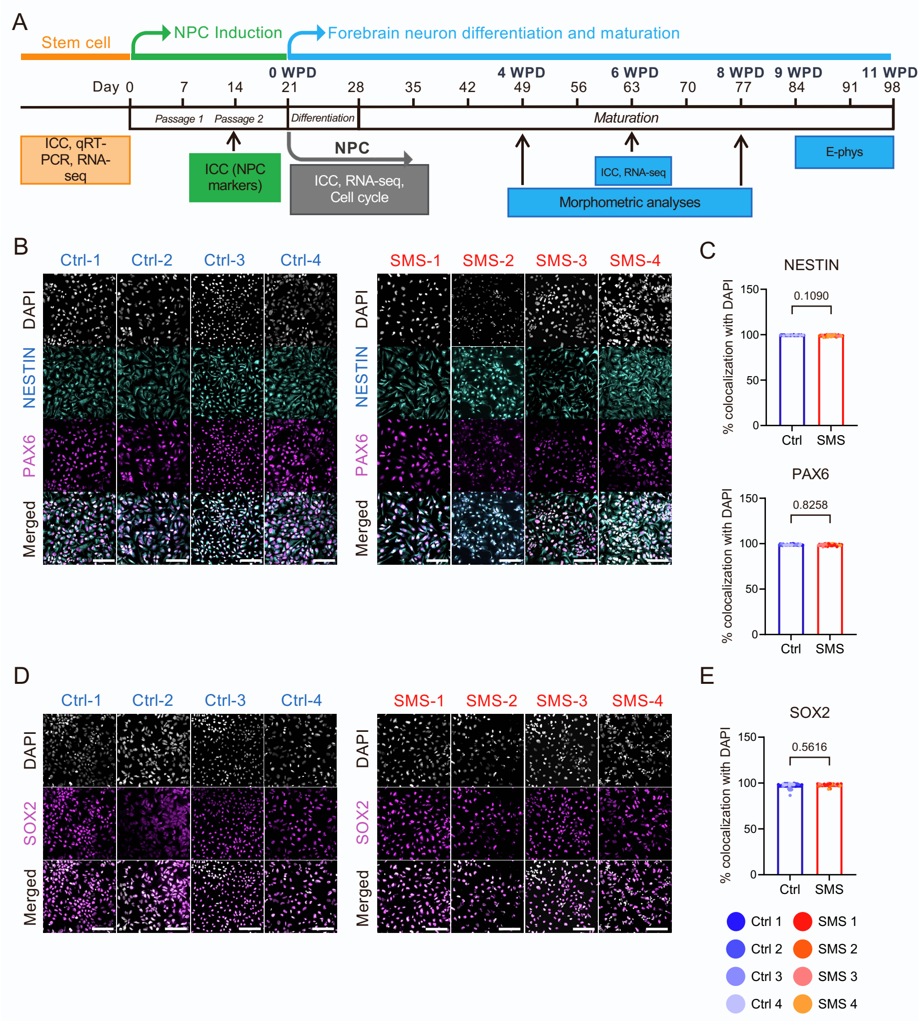

Figure S13: Characterization of Ctrl and SMS hiPSCs-derived neural progenitor cells (NPCs)

(A) Schematic diagram of neural induction from hiPSC into NPC using a monolayer protocol then differentiation into cortical neurons, denoting timepoints of experiments. WPD: weeks post-neural differentiation.

(B) Representative images of Ctrl and SMS hiPSC-derived NPCs stained with NPC markers NESTIN (cyan) and PAX6 (magenta). DAPI-labeled nuclei are in grey. Scale bars: 100µm.

(C) Quantification of NESTIN (top) and PAX6 (bottom) in Ctrl and SMS hiPSC-derived NPCs. n = 32 images per genotype with 8 images per cell line, each dot represents 1 image coloured based on the cell line. NESTIN: U=392.5, p=0.1090; PAX6: U=495, p=0.8258.

(D) Representative images of Ctrl and SMS hiPSC-derived NPCs stained with the NPC marker SOX2 (magenta). DAPI-labeled nuclei are in grey. Scale bars: 100µm.

(E) Quantification of SOX2 in Ctrl and SMS hiPSC-derived NPCs. n = 32 images per genotype with 8 images per cell line, each dot represents 1 image coloured based on the cell line. U=468, p=0.5616.

Data presented as means ± S.E.M.; (**C** and **E**) U- and p-values by two-tailed Mann-Whitney tests.

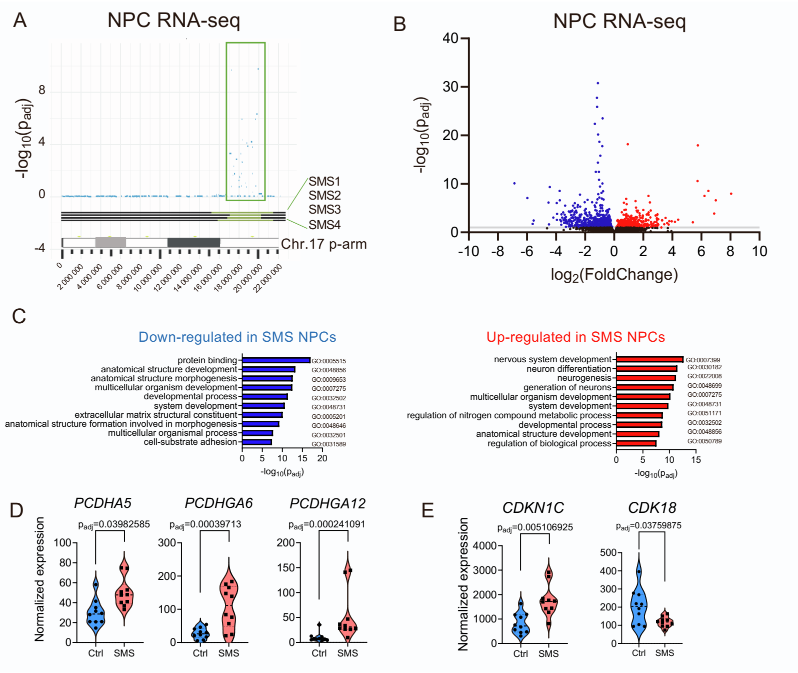

Figure S14: RNA-seq data obtained from Ctrl and SMS hiPSCs-derived neural progenitor cells (NPCs)

(A) A Manhattan plot displaying the genomic landscape of significantly downregulated genes on the chromosome 17 p-arm in SMS NPCs based on -$\log_{10}$ transformed p_{adj} derived from a one-sided Wald test. The x-axis represents the genomic position, and the y-axis corresponds to the significance level of differential expression. The blue dots represent individual genes, with their positions on the x-axis representing their location on chromosome 17p and their heights indicating the significance of their differential expressions. The dark horizontal lines indicate chromosomes, with the green segments corresponding to the regions subjected to del(17)p11.2 in SMS NPCs.

(B) Volcano plots showing the global transcriptomic changes when comparing SMS with Ctrl NPCs. Each dot represents a gene. The $\log_2$ fold change of each gene is represented on the x-axis and the -$\log_{10}$ of its p_{adj} is on the y-axis. Up-regulated genes in SMS NPCs with a p_{adj} less than 0.1 are indicated by red dots. Down-regulated genes in SMS NPCs with a p_{adj} less than 0.1 are indicated by blue dots.

(C) Gene ontology analyses of genes involved in anatomical development and protein binding that were down-regulated in SMS NPCs. By contrast, nervous system developmental and differentiation genes were up-regulated in SMS NPCs. The top 10 GO terms for differentially up-regulated genes (in red), down-regulated genes (in blue), and their respective -$\log_{10}(p_{adj})$ are shown.

(D) Violin plots illustrating down-regulated protocadherin genes in SMS NPCs that are similarly mis-regulated in SMS hiPSCs. Each dot represents a sample (2-3 samples/cell line, n = 20). p_{adj} by Wald test.

(E) Violin plots illustrating selective mis-regulated cell cycle genes in SMS NPCs. Each dot represents a sample (2-3 samples/cell line, n = 20). p_{adj} by Wald test.

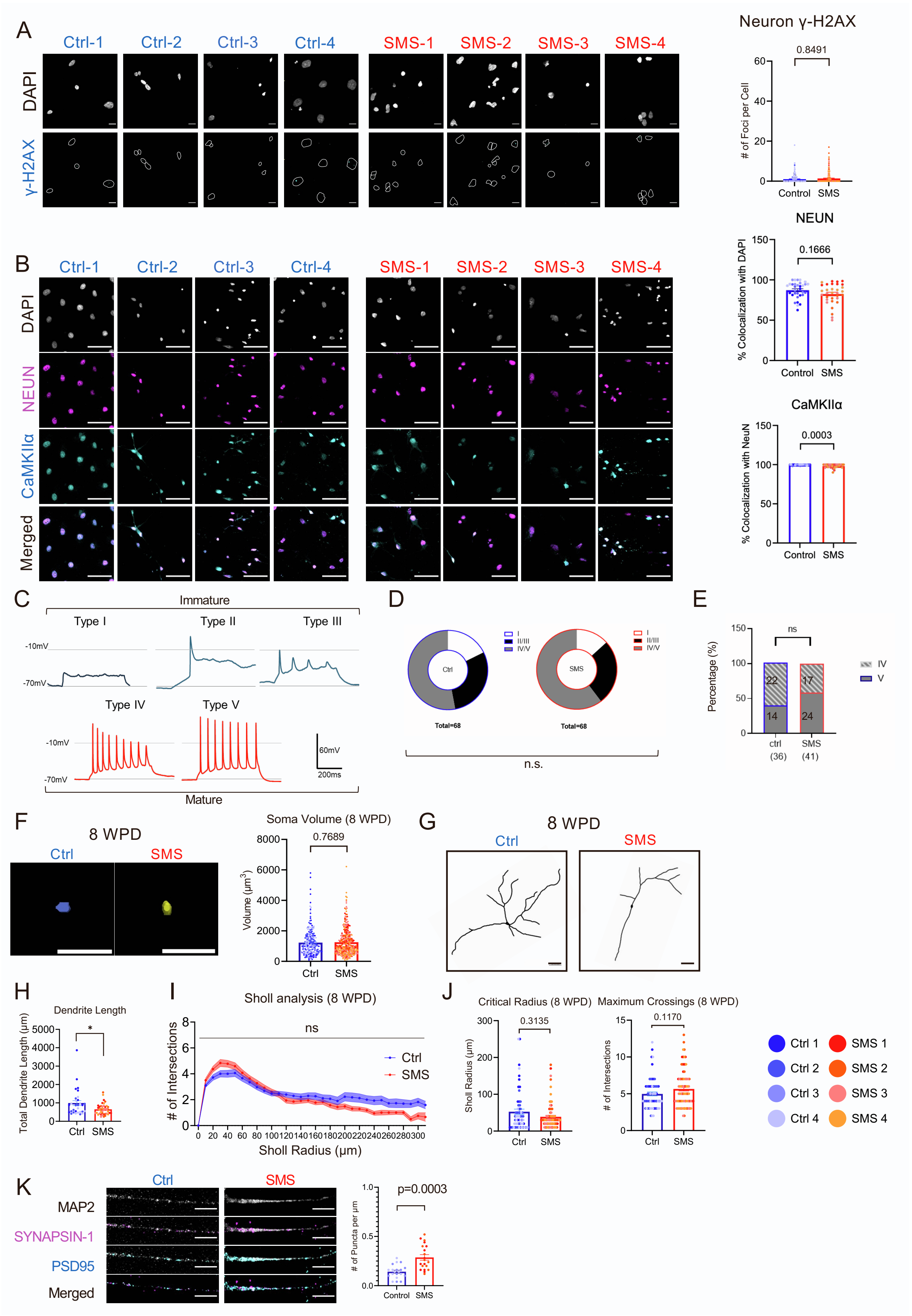
A
Ctrl-1
Ctrl-2
Ctrl-3
Ctrl-4
SMS-1
SMS-2
SMS-3
SMS-4
DAPI
γ-H2AX
Neuron γ-H2AX
0.8491
# of Foci per Cell
Control
SMS
NEUN
0.1666
% Colocalization with DAPI
CaMKIIα
0.0003
% Colocalization with NeuN
B
NEUN
CaMKIIα
Merged
C
Immature
Type I
Type II
Type III
-10mV
-70mV
Type IV
Type V
60mV
200ms
Mature
D
Ctrl
SMS
I
II/III
IV/V
Total=68
n.s.
E
ns
Percentage (%)
IV
V
ctrl
(36)
SMS
(41)
F
8 WPD
Soma Volume (8 WPD)
0.7689
Volume (μm³)
G
8 WPD
H
Dendrite Length
Total Dendrite Length (μm)
I
Sholl analysis (8 WPD)
ns
# of Intersections
Sholl Radius (μm)
J
Critical Radius (8 WPD)
0.3135
Sholl Radius (μm)
Maximum Crossings (8 WPD)
0.1170
# of Intersections
Ctrl 1
Ctrl 2
Ctrl 3
Ctrl 4
SMS 1
SMS 2
SMS 3
SMS 4
K
MAP2
SYNAPSIN-1
PSD95
Merged
p=0.0003
# of Puncta per μm

Figure S15: Morphological and functional characterization of Ctrl and SMS hiPSC-derived cortical neurons

(A) Left: Representative images of Ctrl and SMS hiPSC-derived neurons stained with a double-strand break DNA damage marker γ-H2AX (cyan). Bottom: outlined nuclei for γ-H2AX quantification. Scale bars: 10µm. Right: Quantification showing that a similar number of γ-H2AX foci in Ctrl and SMS neurons. Each dot represents one neuron. U=31490, p=0.8491, two-tailed Mann-Whitney test.

(B) Left: Representative images of Ctrl and SMS hiPSC-derived neurons stained with a mature neuronal marker NEUN (magenta) and an excitatory cortical neuronal marker CaMKIIα (cyan). DAPI-labeled nuclei are in grey. Scale bars: 50µm. Upper right: Quantification showing that a similar number of $DAPI^+$ Ctrl and SMS cells express NEUN. Lower right: Quantification showing that 99.8% of Ctrl $NEUN^+$ neurons and 98.2% of SMS $NEUN^+$ neurons express CaMKIIα. Each dot represents one neuron. NEUN: U=408.5, p=0.1666; CaMKIIα: U=289.5, p=0.0003. U- and p-values by two-tailed Mann-Whitney tests.

(C) Representative traces of hiPSC-derived cortical neuronal action potential (AP) firing (I_{clamp} mode). A small current was injected to maintain the resting membrane potential around –70mV. Next, APs were evoked by current steps (increment, 10pA, 500ms, from –110 pA to the deplorization step causing membrane potential rises and APs to fail). A successful AP firing is defined as a fast depolarization overshoot greater than –10 mV. Immature neurons include Types I-III: Type I, failed to fire AP; Type II: single AP firing; Type III, single AP firing followed by unsuccessful events. Mature cortical neurons include Types IV and V: Type IV, repetitive AP firing with decaying overshoot values; Type V, repetitive and uniform AP firing.

(D) Pie charts showing a similar proportion of hiPSC-derived Ctrl (blue border) and SMS (red border) cortical neurons in each AP firing category. n.s., not significantly different, *Chi-square*=0.2506, *df*=2, p=0.8822, Chi-squared analysis.

(E) A similar percentage of Ctrl (blue border) and SMS (red border) neurons in type IV (grey stripes) and type V (grey bar) categories. n.s., not significantly different, chi-squared analysis. *Chi-square*=0.5951, *df*=1, p=0.4405, Chi-squared analysis.

(F) Left: Representative 8 WPD soma 3D images of Ctrl and SMS cortical neurons transduced with myrGFP lentivirus. Scale bar: 50µm. Right: Bar plot showing a similar soma volume between Ctrl and SMS neurons at 8 WPD. n = 225-300 cells per genotype with 75 cells per cell line. Each dot represents one soma coloured based on the cell line. t=0.2939, *df*=523, p=0.7689, unpaired t-test.

(G) Representative 3D reconstituted neuron images of myrGFP-transduced Ctrl and SMS hiPSC-derived neurons at 8 WPD. Scale bar: 50µm.

(H) Quantification showing $myrGFP^+$ Ctrl hiPSC-derived neurons have longer dendrites compared to SMS neurons at 8WPD. Ctrl group has 30 neurons and SMS group has 40 neurons, with 10 neurons per cell line. Each dot represents one neuron. U=393, p=0.0136, two-tailed Mann-Whitney test.

(I) Sholl analysis of Ctrl and SMS hiPSC-derived neurons at 8 WPD. n = 60 Ctrl neurons and 80 SMS neurons. Presented as differences between means (line) ± S.E.M (shade). p-values >0.05 for all bins, calculated by two-sided multiple t-tests.

(J) Quantification of Sholl critical radius (left) and maximum crossings (right) of Ctrl and SMS hiPSC-derived neurons at 8 WPD. Ctrl group has 60 neurons and SMS group has 80 neurons, with 20 neurons per cell line. Each point represents one neuron. Critical radius: U=2163, p=0.3135; Max crossings: U=2033, p=0.1170. U- and p-values by two-tailed Mann-Whitney tests.

(K) Quantification of excitatory synapse density (presynaptic SYNAPSIN-1^{+} and postsynaptic PSD95^{+}) in Ctrl (Ctrl-3 and Ctrl-4) and SMS (SMS-1 and SMS-2) cortical neurons at 6 WPD. Left: Representative images of Ctrl and SMS neurons stained with MAP2 (grey), SYANPSIN-1 (magenta), and PSD95 (cyan). Scale bar: 10μm. Right: Quantification of the number of puncta (SYANPSIN-1^{+} and PSD95^{+}) per μm. Each dot represents one 50μm segment, n=20 segments per genotype with 10 segments per cell line. U=62, p=0.0003, two-tailed Mann-Whitney test.

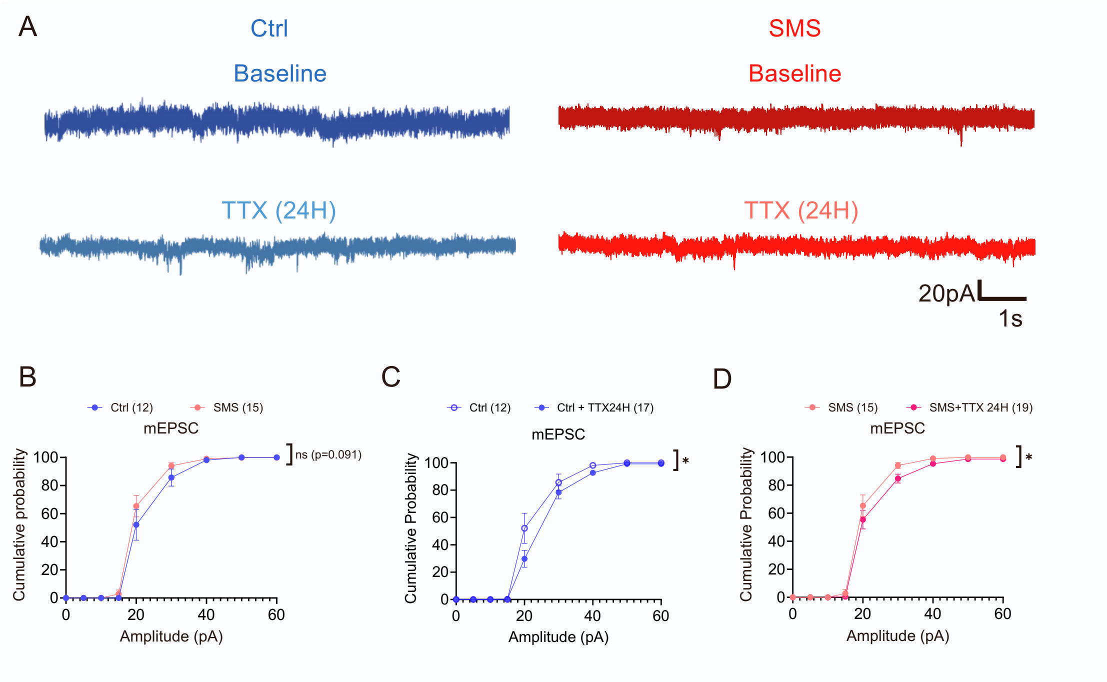

Figure S16: Normal glutamatergic synaptic transmission and up-scaling in Ctrl and SMS hiPSCs-derived 2D cortical neurons

(A) Representative traces of mEPSCs in Ctrl and SMS hiPSC-derived cortical neurons (holding potential at −70 mV). Top: baseline condition with acute TTX treatment to block AP; Bottom: after 24 hours of TTX treatment to silence neuronal firing and induce synaptic upscaling.

(B-D) Distribution of mEPSC amplitudes in Ctrl vs SMS neurons at baseline (genotype=$F(1,325)=2.873$, $p=0.091$; amplitude=$F(12,325)=510.6$, $p<0.0001$) **(B)**. TTX treatment for 24 hours (TTX 24H) in Ctrl neurons induced a right shift in the cumulative probability plot (treatment=$F(1,351)=6.450$, $p=0.0115$; amplitude=$F(12,351)=549.0$, $p<0.0001$) **(C)**, indicating a normal synaptic upscaling. Similarly, TTX treatment for 24 hours in SMS neurons induced synaptic upscaling (treatment=$F(1,416)=6.343$, $p=0.0122$; amplitude=$F(12,416)=817.0$, $p<0.0001$) **(D)**. F(DFn, DFd) and *p*-values were calculated by two-way ANOVA with post hoc Šídák multiple comparisons.

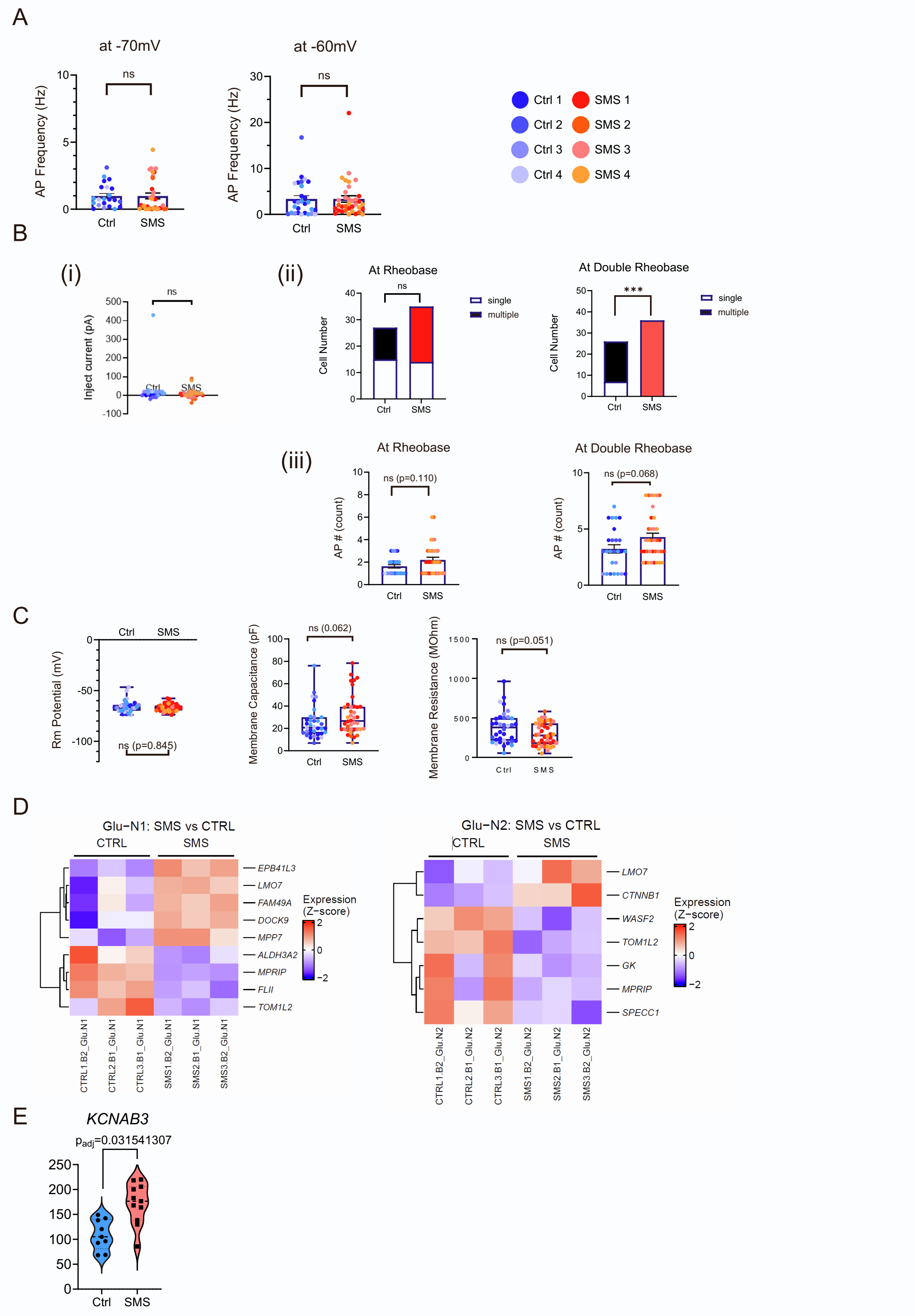
A
at -70mV
at -60mV
AP Frequency (Hz)
ns
Ctrl
SMS
Ctrl 1
Ctrl 2
Ctrl 3
Ctrl 4
SMS 1
SMS 2
SMS 3
SMS 4
B
(i)
Inject current (pA)
(ii)
At Rheobase
At Double Rheobase
Cell Number
single
multiple
***
(iii)
AP # (count)
ns (p=0.110)
ns (p=0.068)
C
Rm Potential (mV)
ns (p=0.845)
Membrane Capacitance (pF)
ns (0.062)
Membrane Resistance (MOhm)
ns (p=0.051)
D
Glu−N1: SMS vs CTRL
Glu−N2: SMS vs CTRL
CTRL
SMS
EPB41L3
LMO7
FAM49A
DOCK9
MPP7
ALDH3A2
MPRIP
FLII
TOM1L2
CTNNB1
WASF2
GK
SPECC1
Expression (Z−score)
CTRL1.B2_Glu.N1
CTRL2.B1_Glu.N1
CTRL3.B1_Glu.N1
SMS1.B2_Glu.N1
SMS2.B1_Glu.N1
SMS3.B2_Glu.N1
CTRL1.B2_Glu.N2
CTRL2.B1_Glu.N2
CTRL3.B1_Glu.N2
SMS1.B2_Glu.N2
SMS2.B1_Glu.N2
SMS3.B2_Glu.N2
E
KCNAB3
p adj=0.031541307

Figure S17: Intrinsic excitability of Ctrl and SMS hiPSCs-derived 2D cortical neurons

(A) Spontaneous AP firing frequency at a holding potential of -70mV (left) and -60mV (right). The silenced cortical neurons were excluded. No significant differences were found between Ctrl and SMS cortical neurons. -70mV: *U*=306, *p*=0.3318; -60 mV: *U*=468, *p*=0.7650; two-tailed Mann-Whitney tests.

(B) At I_{clamp}, (i) the injected currents in Ctrl and SMS neurons to evoke AP at the rheobase were similar, *U*=402.5, *p*=0.235, two-tailed Mann-Whitney test; (ii) There were similar numbers of single and multiple firing Ctrl and SMS neuron populations at the rheobase (*chi-cauqre*=0.922, *df*=1, *p*=0.3368, Chi-squared analysis) and more SMS neurons fired multiple APs at double rheobase (*chi-cauqre*=10.93, *df*=1, *p*=0.0009, Chi-squared analysis) (iii) The AP numbers were similar between Ctrl and SMS cortical neurons. Rheobase: *U*=367, *p*=0.110; Double Rheobase: *U*=342, *p*=0.0681, *U*- and *p*-values by two-tailed Mann-Whitney tests.

(C) The membrane properties of Ctrl and SMS cortical neurons. There were no significant differences in resting membrane potential, membrane capacitance, and membrane resistance. Rm: *U*=521, *p*=0.845; membrane capacitance: *U*= 524, *p*=0.062; membrane resistance: *U*= 561.5, *p*=0.051; *U*- and *p*-values by two-tailed Mann-Whitney tests.

(**D**) Heatmaps showing that Glu-N1 and Glu-N2 excitatory neurons from SMS organoids overexpressed several synaptic genes. By contrast, synaptic genes down-regulated in SMS excitatory neurons belong to del(17)p11.2.

(E) Violin plots illustrating that bulk RNA-seq detected up-regulated *KCNAB3* in SMS neurons when compared to Ctrls. Each dot represents a sample. p_{adj} by Wald test.

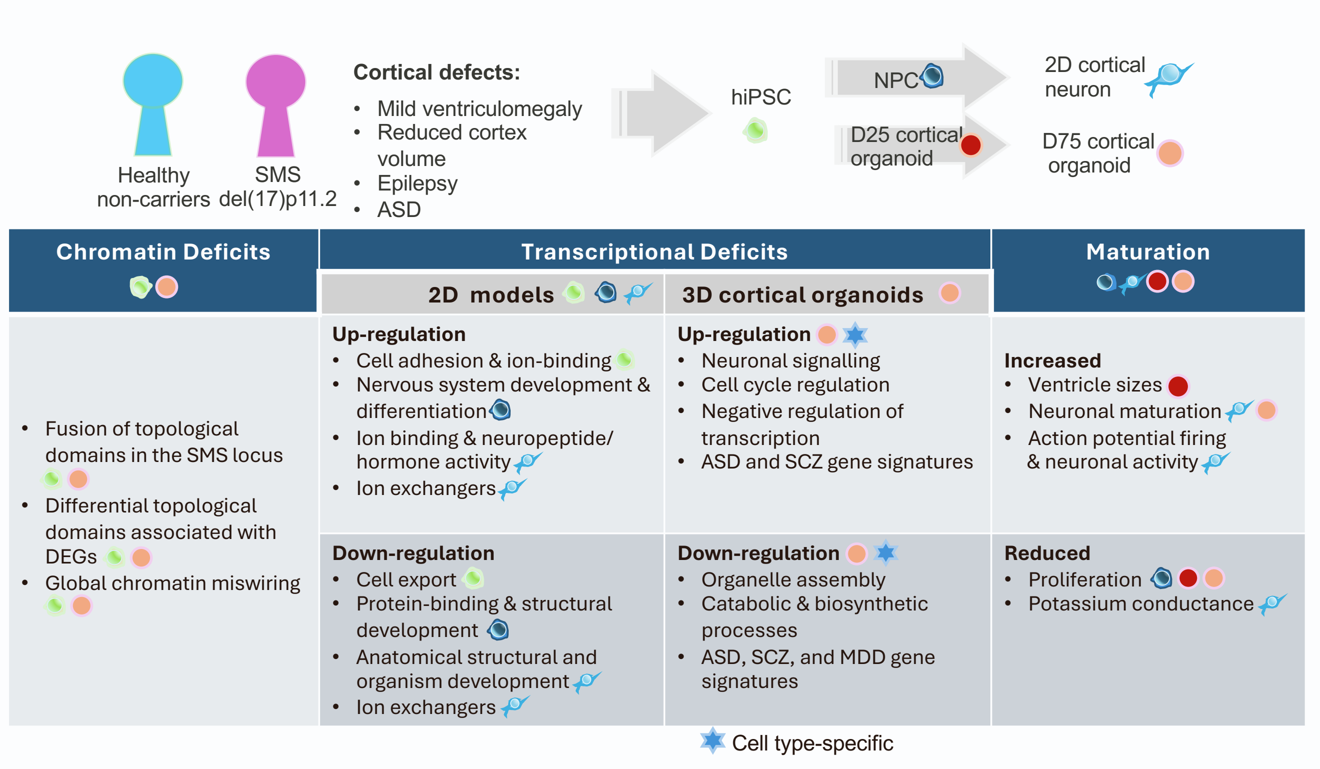

Figure S18: Summary of the findings

Table S1: Information on hiPSC lines

Name	Coriell number	Disease	Genomic deletions	Biopsy Sources	Tissue types	Sex	Age
Ctrl-1	GM23279	Apparently healthy	None	Skin	hiPSC	Female	36 years
Ctrl -2	GM23720	Apparently healthy	None	B-Lymphocyte	hiPSC	Female	22 years
Ctrl -3	GM23716	Apparently healthy	None	Skin	hiPSC	Female	37 years
Ctrl -4	AG27875	Apparently healthy	None	Skin	hiPSC	Male	37 years
SMS-1	NA	SMS	Chr17 del:15,175,307-21,519,100	Skin	hiPSC (reprogrammed from GM25367)	Female	21 years
SMS-2	GM27918	SMS	Chr17 del:16,881,282-20,121,029	Skin	hiPSC	Female	20 years
SMS-3	NA	SMS	Chr17 del:16,769,800-20,126,017	Skin	hiPSC (reprogrammed from GM25371)	Female	12 years
SMS-4	NA	SMS	Chr17 del:15,777,172-20,554,628	Skin	hiPSC (reprogrammed from GM24311)	Female	23 years

Table S2: Clinical features of SMS patients

SMS patients	Clinical features
SMS-1	Diagnosed at age 7 years via FISH test; brachycephaly; midface retrusion (hypoplasia); broad nasal bridge; down-turned upper lip; prognathism; synophrys; defective vision; strabismus; hearing defect; decreased pain sensitivity; dental abnormalities; hoarse voice; pes planus; scoliosis; syndactyly; tetralogy of Fallot; constipation; short stature; overweight/obesity; high total cholesterol; self-injurious behavior
SMS-2	Diagnosed at age 2 years via FISH test; breech pregnancy; laryngomalacia; defective vision; hearing defect; decreased pain sensitivity; coloboma; midface retrusion (hypoplasia); dental abnormalities; down-turned upper lip; scoliosis; cardiac issues include: ASD, VSD, pulmonary stenosis; intellectual disability (52 IQ/DQ); self-injurious behavior
SMS-3	Diagnosed at 9 years of age; brachycephaly; large forehead; down-turned upper lip; hearing defect; hypotonia; decreased pain sensitivity; abnormal gait; brachydactyly; pes planus; scoliosis; constipation; short stature; speech delay; overweight/obese; decreased tolerance to exercise; intellectual disability; sleep disturbance; self-hugging; immunologic abnormalities; ear infections
SMS-4	Diagnosed at 14 years; dysmorphic facial features; hypotelorism; dental abnormalities; hoarse voice; dry skin; brachydactyly; scoliosis; short stature; hypotonia; obesity; defective vision, strabismus; hearing defect; ataxia, decreased pain sensitivity; constipation; fine motor delay; gross motor delay; speech delay; intellectual disability; ADHD; oppositional defiant disorder; obsessive compulsive disorder; sleep disturbance; repetitive behaviors; self-injurious behaviors; anxiety disorder; disruptive behavior disorder; high total cholesterol

Table S3: Hi-C contacts per chromosome for each sample (Excel file provided)

Table S4: qRT-PCR primer sequences

Gene	Forward	Reverse
RAI1	CCTCAGCATTCCCAGTCCTTC	CTGTGCAACTCTTATAGGAGTGG
GAPDH	AAGGTGAAGGTCGGAGTCAA	AATGAAGGGGTCATTGATGG
PCDHA3	CGTGTACTTGATCGTCGCCAT	CAAGGAGGAAGGCTAGGGCTA
PCDHA9	CGCGGTGTCTAGCCTGTTG	CCCGTTCGCTCTGTAGATCC

Table S5: Number of samples for each line used in each experiment (Excel file provided)
