# Supplementary material for: Molecular and developmental deficits in Smith-Magenis syndrome human stem cell-derived cortical neural models
Source: Am J Hum Genet. 2025 Aug 28;112(10):2338–62. doi: 10.1016/j.ajhg.2025.07.020 (PMC12696504; doi:10.1016/j.ajhg.2025.07.020)
Supplement: Document S2. Article plus supplemental information [file mmc4.pdf]

Molecular and developmental deficits in Smith-Magenis syndrome human stem cell-derived cortical neural models

Graphical abstract

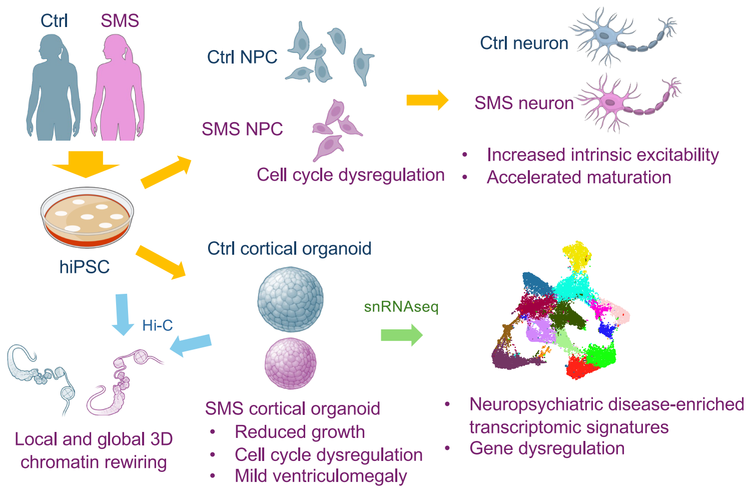

Authors

Yu-Ju Lee, Ya-Ting Chang, Yoobin Cho, ..., Qihuang Zhang, Xiaojing Gao, Wei-Hsiang Huang

Correspondence

wei-hsiang.huang@mcgill.ca

Smith-Magenis syndrome (SMS) is caused by del(17)p11.2 and haploinsufficiency of *RAI1*. To understand disease etiology, we engineered SMS hiPSC-derived 2D cortical neurons and 3D organoid models. We found that del(17) p11.2 disrupts 3D chromatin wiring and transcriptional regulation, causing defective neural growth, differentiation, and cellular hyperexcitability.

Lee et al., 2025, The American Journal of Human Genetics *112*, 2338–2362

October 2, 2025 © 2025 The Authors. Published by Elsevier Inc. on behalf of American Society of Human Genetics.

https://doi.org/10.1016/j.ajhg.2025.07.020

ARTICLE

Molecular and developmental deficits in Smith-Magenis syndrome human stem cell-derived cortical neural models

Yu-Ju Lee,[1,5] Ya-Ting Chang,[1,5] Yoobin Cho,[1] Max Kowalczyk,[1] Adrian Dragoiescu,[1] Alain Pacis,[2] Senthilkumar Kailasam,[2] François Lefebvre,[2] Qihuang Zhang,[3] Xiaojing Gao,[4] and Wei-Hsiang Huang[1,*]

Summary

Smith-Magenis syndrome (SMS) is a genomic disorder caused by the deletion of a chromosomal region at 17p11.2. Individuals with SMS are frequently diagnosed with autism and have profound cortical deficits, including reduced cortex volume, mild ventriculomegaly, and epilepsy. Here, we developed human induced pluripotent stem cell (hiPSC)-derived neuronal models to understand how del (17)p11.2 affects cortical development. Hi-C experiments identified local fusion and global reorganization of topological domains, as well as genome-wide miswiring of chromatin three-dimensional (3D) interactions in SMS hiPSCs and 3D cortical organoids. Single-nucleus RNA sequencing of SMS cortical organoids identified neuropsychiatric disease-enriched transcriptional signatures and dysregulation of genes involved in catabolic and biosynthetic pathways, cell-cycle processes, and neuronal signaling. SMS cortical organoids displayed reduced growth, enlarged ventricles, impaired cell-cycle progression, and accelerated neuronal maturation. Through the use of a complementary hiPSC-derived 2D cortical neuronal model, we report that SMS cortical neurons exhibited accelerated dendritic growth, followed by neuronal hyperexcitability associated with reduced potassium conductance. Our study demonstrates that del (17)p11.2 disrupts multiple steps of human cortical development, from chromatin wiring, transcriptional regulation, cell-cycle progression, and morphological maturation to neurophysiological properties, and hiPSC-derived models recapitulate key neuroanatomical and neurophysiological features of SMS.

Introduction

Heterozygous deletion of the chromosomal region 17p11.2 (del(17)p11.2) causes Smith-Magenis syndrome (SMS; MIM: 182290), a genomic disorder associated with growth failure, intellectual disability, metabolic defects, obesity, self-injury, hearing loss, epilepsy, and neuropsychiatric features.[1] 90% of individuals with SMS are diagnosed with autism spectrum disorder (ASD),[2] and SMS is unique among ASDs because of the reversed sex ratio for autistic traits (male:female = 1:3) relative to other ASDs (male:female = 4:1).[3] 10% of individuals with SMS have heterozygous mutations in retinoic acid-induced 1 (*RAI1*; MIM: 607642), a gene encompassed within del(17)p11.2, and experience a milder form of SMS.[4] However, 90% of individuals with SMS harbor del(17)p11.2 and display severe intellectual disability as well as speech and sensory issues not seen in individuals with *RAI1* mutations.[5,6] The neuroanatomical hallmarks of SMS include reduced gray matter volume in the insular cortex[7] and mild ventriculomegaly (enlarged ventricles) with occasional hydrocephalus,[8–10] which can be identified prenatally.[11,12] Enlarged ventricles and reduced cortical volume are common in ASD and likely result from impaired neural progenitor cell (NPC) progression and corticogenesis.[13] How del(17) p11.2 leads to cortical malformation and hyperexcitability remains unclear.

SMS mouse models recapitulate aspects of human pathophysiology, such as obesity,[14,15] social dysfunction,[16] and epilepsy.[17] However, unlike in individuals with SMS, the cortical structures of SMS mouse models remain largely intact.[17] This could be attributed to several unique aspects of human neocortical development. First, while both human and mouse cortical neurons are generated from NPCs lining the ventricles, human NPCs have distinct cell-cycle regulation—cell cycles are three times longer than those of mice. Human NPCs also have a prolonged proliferative phase that delays neurogenic onset, allowing greater expansion of the NPC pool and contributing to the uniquely complex brain architectures in humans.[18,19] Additionally, human and mouse neocortices have distinct cell types, including unique types of radial glial cells[20,21] as well as different chromatin spatial organization and gene-expression patterns[20,21] and excitability.[20,22,23] During human cortical development, NPCs undergo changes in chromatin topology, gene expression, morphology, and neuronal excitability to

[1]Department of Neurology and Neurosurgery, Centre for Research in Neuroscience, The Research Institute of the McGill University Health Centre, Montreal, QC H3G 1A3, Canada; [2]Canadian Centre for Computational Genomics, McGill University, Montreal, QC H3A 0G1, Canada; [3]Department of Epidemiology, Biostatistics and Occupational Health, McGill University, Montreal, QC H3A 1Y7, Canada; [4]Department of Chemical Engineering, Stanford University, Stanford, CA 94305, USA
[5]These authors contributed equally
*Correspondence: wei-hsiang.huang@mcgill.ca
https://doi.org/10.1016/j.ajhg.2025.07.020.
© 2025 The Authors. Published by Elsevier Inc. on behalf of American Society of Human Genetics.
This is an open access article under the CC BY license (http://creativecommons.org/licenses/by/4.0/).

acquire features of mature neurons.[24,25] Each of these processes, including the formation of 3D nuclear architectures,[26] cell-cycle regulation in NPCs,[27] expression of cell-type-specific genes,[28] and the development of proper neuronal morphology[29] and excitability[30–32] are susceptible to genetic mutations. Developing human induced pluripotent stem cell (hiPSC)-derived models is essential to recapitulate the human pathophysiology of SMS. To investigate how del(17)p11.2 impacts human cortical development, we differentiated hiPSCs originating from individuals with SMS into NPCs and then into two-dimensional (2D) cortical neurons[33–35] or 3D patterned dorsal cortical organoids.[36] We characterized hiPSC-derived SMS cortical neural models with functional genomic and neurophysiological tools. At the molecular level, SMS organoids showed strong alterations in local and global chromatin contact patterns and transcriptional perturbation in various cell types. We identified gene-expression signatures that mirrored that of other neuropsychiatric diseases. At the cellular level, SMS organoids and NPCs showed reduced growth and had ventriculomegaly-like phenotypes that could be attributed to reduced progenitor proliferative capacity. SMS neurons had increased dendritic growth initially, followed by hyperexcitability driven by a reduction in potassium conductance. Altogether, this study establishes 2D and 3D hiPSC-derived neural models of SMS and reveals molecular and cellular pathogenic mechanisms by which del(17)p11.2 impacts human corticogenesis.

Material and methods

Derivation, characterization, and maintenance of hiPSCs

The use of hiPSCs in this research was approved by the research ethics board of the Research Institute of the McGill University Health Center, with proper informed consent obtained. All but one of the hiPSC lines (Ctrl-4) were derived from female individuals, given the higher ratio of ASD symptomatology in female individuals with SMS compared to male individuals.[3] Control (Ctrl) hiPSCs from four healthy individuals and an hiPSC line derived from an individual carrying del(17)p11.2 were obtained from the Montreal Neurological Institute (Ctrl-3 and Ctrl-4) and the Coriell Institute (Ctrl-1, Ctrl-2, and SMS-2). To generate three additional hiPSC lines carrying del(17)p11.2, fibroblasts harvested from three individuals clinically diagnosed with SMS (Coriell Institute SMS-1: GM25367; SMS-3: GM25371; and SMS-4: GM24311) were reprogrammed into hiPSCs with the integration-free Sendai virus (Cytotune 2.0 kit, Life Technologies). hiPSCs were cultured under feeder-free conditions in mTeSR Plus medium (STEMCELL Technologies) with 100 μg/mL Primocin (InvivoGen) in Matrigel (Corning Matrigel hESC-Qualified Matrix, 354277)-coated dishes. Cells were incubated in a humidified incubator with 5% CO_2 at 37°C. All hiPSC lines were characterized by karyotyping, free of mycoplasma, and expressed pluripotency markers. See Tables S1 and S2 for information regarding each line and Table S5 for lines used in each experiment.

Generation of 3D cortical organoids from hiPSCs

Patterned human cortical organoids were generated using the AggreWell system[36] (STEMCELL) according to the manufacturer's instructions. In brief, hiPSCs were dissociated into single cells using Gentle Cell Dissociation Reagent (GCDR) (STEMCELL), and cells from each well were centrifuged with 500 μL of Anti-Adherence Rinsing Solution at 1,300 × g for 3 min. 1.5–3 million cells were seeded in each prepared well by spinning at 100 × g for 5 min in 1.5 mL of STEMdiff Seeding Medium I with ROCK inhibitor Y-27632 (10 μM). For the next 5 days, 1 mL per well of fresh STEMdiff Forebrain Organoid Formation Medium I was carefully exchanged. On day 6, one AggreWell of embryoid bodies was filtered through a 37-μm strainer and transferred to six wells of a 6-Well Ultra-Low Adherent Plate (Corning) containing 2 mL of STEMdiff Forebrain Organoid Expansion Medium. STEMdiff Forebrain Organoid Expansion Medium was changed every other day until day 25, at which point the medium was changed to STEMdiff Forebrain Organoid Differentiation Medium. Organoids were cultured in differentiation medium until day 43. Following day 43, organoids were cultured in STEMdiff Forebrain Organoid Maintenance Medium indefinitely. Maintenance medium was changed every 2–3 days, and the volume was increased to 2.2–3 mL when necessary.

Generation of 2D NPCs and cortical neurons from hiPSCs

NPCs were differentiated from hiPSCs using the STEMdiff SMADi Neural Induction Kit (STEMCELL). In brief, hiPSC colonies were dissociated into single cells using GCDR and seeded in Matrigel-coated plates in STEMdiff SMADi Neural Induction medium with ROCK inhibitor Y-27632 (10 μM). The medium was changed daily with STEMdiff SMADi Neural Induction medium without ROCK inhibitor. Cells were passaged every 7 days with Accutase (STEMCELL) for three passages. NPC markers were examined at the second passage. After the third passage, NPCs were cultured in STEMdiff Neural Progenitor medium in Matrigel-coated plates for NPC experiments.

To generate 2D cortical neurons, NPCs were first cultured in STEMdiff Neural Progenitor medium in poly-L-ornithine (PLO) (Sigma-Aldrich)/laminin (Invitrogen)-coated plates. The next day, the medium was changed to STEMdiff Forebrain Neuron Differentiation medium (STEMCELL), and a daily medium change was performed for 6 days. Neuronal precursors were passaged using Accutase and seeded in STEMdiff Forebrain Neuron Maturation medium in PLO/laminin-coated coverslips or dishes. A half-medium change was performed every 3 days until the neurons were ready for experiments.[33–35]

Quantification of cell cycle using flow cytometry in NPC and EdU chase in cortical organoids

To assess the cell-cycle profile, NPCs were seeded in six-well plates with 2.5×10^6 cells/well and harvested 48 h after seeding using Accutase. After centrifugation at 400 × g for 5 min, cells were washed twice with cold phosphate-buffered saline (PBS). Cells were fixed with 70% ethanol and stored at −20°C. On the day of the experiment, cells were centrifuged at 300 × g for 5 min at 4°C to remove ethanol. After being washed twice with cold PBS, cells were stained with 50 μg/mL propidium iodide (Thermo Fisher P3566) with 100 μg/mL RNase A (Thermo Fisher EN0531) in darkness for 1 h, then analyzed by flow cytometry (BD FACSCanto II). Each cell line has three biological replicates, and

data were analyzed using FlowJo software with the Watson Pragmatic Algorithm (Tree Star).[37]

To assess short- and long-term cell-cycle regulation in day-25 cortical organoids, 10 μM 5-ethynyl-2′-deoxyuridine (EdU) (Click-iT Plus EdU Cell Proliferation Kit, Thermo Fisher) was administered for 30 min and 24 h, respectively. The EdU-treated organoids were stained with 0.5% trypan blue in PBS for 4 min at room temperature, fixed in 4% paraformaldehyde, and processed for cryosection and immunocytochemistry (ICC). Images were taken using Nikon Epi-FL (CFI 40× oil lens, NA 1.3) and Olympus FV-1000 (60×, NA 1.3) confocal laser scanning microscopes. Image stacks were taken with a step size of 1.0 μm (four optical sections per image). The colocalization between EdU^+, $Ki67^+$, and $DAPI^+$ cells was calculated using ImageJ (Fiji v.2.3.0) software. One to three images per organoid and 1–3 organoids per line were analyzed. On average, we counted 2,300 cells per organoid.

Hi-C and data analysis

The Hi-C libraries were generated using hiPSCs (four control libraries [Ctrl 1–4] and four SMS libraries [SMS 1–4], n = one 10-cm dish per library) and day-75 cortical organoids (three control libraries [Ctrl 2–4] and four SMS libraries [SMS 1–4], $n = 5$ organoids per library) following the manufacturer's instructions for the Arima High Coverage HiC Kit. Libraries were sequenced on the Illumina NovaSeq 6000 sequencing system (150-bp paired-end sequencing). The Hi-C reads were processed using hic v.2.1.0 pipeline within nf-core[38] using the following options: "–digestion arima –tads_caller hicexplorer –min_mapq 10 –genome GRCh38." The reads were mapped using HiC-Pro 3.1.0[39] with the bowtie2 arguments "–very-sensitive -L 30 –score-min L,-0.6,-0.2 –end-to-end –reorder." Individual mapping was performed for all replicates, ensuring that only valid pairs were utilized for subsequent steps. Contact maps were generated at resolutions of 10 kb, 40 kb, 25 kb, and 1 Mb using Cooler.[40] Most of the post-processing and visualization were done using HiCExplorer.[41] The contact matrices were balanced using the ICE algorithm and were saved in .hic and .cool file formats. The relationship between contact frequencies and genomic distance was calculated using hicPlotDistVsCounts. We analyzed Hi-C data from hiPSC_CTRL (Ctrl group) and hiPSC_SMS (experimental group) samples to identify differential chromosomal interactions. Replicate Hi-C datasets were processed from .cool files at 1 Mb resolution, converted to InteractionSet objects to store interaction counts, and filtered for low-abundance interactions using the filterDirect and filterDiag functions from diffHic.[42] Control replicates were merged using the hicSumMatrices module from HiCExplorer to create a single aggregated control matrix for comparison with individual ICE-corrected SMS samples. All matrices, including individual SMS samples, were bias corrected using the ICE algorithm (hicCorrectMatrix) and normalized with hicNormalize for cross-sample comparability. Only high-confidence interactions were retained. LOESS normalization was applied to adjust for distance-dependent decay of interactions, and dispersion was estimated using the estimateDisp function from edgeR,[43] which also corrects for overdispersion in sparse Hi-C count data. Differential interactions between conditions were tested using a generalized linear model, and multiple testing was controlled using the Benjamini-Hochberg procedure (false discovery rate [FDR] < 0.05).[42] Differential interaction maps were also generated using the hicCompareMatrices tool in HiCExplorer with the "–operation Log2ratio" option and visualized using hicPlotMatrix. Topologically associated domains (TADs) were identified at 40-kb resolution using hicFindTADs with the "–correctForMultipleTesting fdr" option. To assess differential TADs, we used the hicDifferentialTAD module with the "-p 0.01 -t 1 -mr one" option, comparing target samples against the combined control dataset. Intra-TAD and inter-TAD regions with FDR < 0.01 were concatenated and sorted by genomic position to generate consensus BED files. The statistical overlap between differentially expressed genes (DEGs) and differential TAD boundaries was tested using Fisher's exact test. For A/B compartment analysis, eigenvector decomposition was performed on balanced Hi-C matrices using Cooltools (eigs-cis). Visualization of TADs and differential interaction maps employed hicPlotTADs, pyGenomeTracks, and custom Python/R scripts. Contacts for each chromosome are listed in Table S3.

snRNA-seq and data analysis

Nuclei from day-75 cortical organoids (three control libraries [Ctrl 1–3] and three SMS libraries [SMS 1–3], $n = 5$ organoids per library; all single-nucleus RNA sequencing [snRNA-seq] libraries were derived from female samples) were isolated using the Miltenyi Nuclei Extraction Buffer (Miltenyi Biotec), following the manufacturer's guidelines with gentle MACS Dissociation and C tubes. Upon isolation, the nuclei were counted on the Nexcelom Cellaca MX. snRNA libraries were generated using 10× Genomics' Single Cell Gene Expression kits (RNA-seq and ATAC bundle for Ctrl2, Ctrl3, and SMS2; RNA-seq for Ctrl1, SMS1, and SMS3), loaded onto the Chromium instrument for downstream analysis, and processed according to the manufacturer's standard specifications. The sequencing libraries were evaluated for quality on the Agilent TapeStation (Agilent Technologies) and quantified using a Qubit 2.0 fluorometer (Invitrogen). Libraries were quantified using qPCR (Applied Biosystems) before loading onto an Illumina NovaSeq instrument. The samples were sequenced at a configuration compatible with the recommended guidelines outlined by 10× Genomics.

Droplet libraries were processed using the Cell Ranger count pipeline (10× Genomics).[44] Sequencing reads were aligned to the GRCh38 human reference genome, and transcript counts were quantified for each annotated gene within every cell. Count matrices (genes × cells) were loaded into the R package Seurat[45] for quality control and downstream analyses. Low-quality cells were filtered out using the criteria that (1) the number of detected genes is ≤1,000 and (2) the percentage of mitochondrial RNA is >5%. Cell doublets were detected and removed using the R package scDblFinder.[46] Following SCTransform normalization, individual samples were integrated using the HarmonyIntegration method. Uniform manifold approximation and projection (UMAP) dimension reduction was generated based on the first 15 principal components (PCs). A nearest-neighbor graph using the first 15 PCs was calculated using the FindNeighbors function, followed by clustering using the FindClusters function. Cluster-specific marker genes were identified using the function FindMarkers with the cutoffs $\log_2$ fold change >0.5 and adjusted p value <0.05 (upregulated genes only). Clusters were manually annotated to cell types by canonical markers. Differential composition analysis was performed using the R package sccomp.[47]

Per-cluster differential expression testing between two groups was conducted using a pseudobulk approach. We excluded lowly expressed genes with an average read count lower than 10 across all samples/cell types. Raw counts were normalized using edgeR's TMMwsp algorithm[48] and were then transformed to $\log_2$ counts

per million (log_2CPM) using the voomLmFit function implemented in the R package limma.[49] To assess differences in gene-expression levels, we fitted a linear model using the lmfit function and considered batch effects. Nominal p values were corrected for multiple testing using the Benjamini-Hochberg method. Significantly differentially expressed genes (DEGs) were obtained using a p-adjusted value of <0.2. Over-representation analysis was performed using enrichR.[50] Gene set enrichment analysis based on pre-ranked gene list by t-statistic was performed using the R package fgsea (http://bioconductor.org/packages/fgsea/). To visualize the Gene Ontology (GO) hierarchy, nested pie charts were used to show significant GO enrichments in a hierarchical parent-child manner following GO redundancy reduction. The R package rrvgo[51] was used to simplify the redundancy of GO lists based on semantic similarity. It groups terms that are at least within a similarity below the threshold and selected as the group representative/parent the term with the higher score within the group (p values with minus-log-transform as scores). The nested pie charts show only the top five parent GO terms and their respective top five child GO terms. The width of the pie is correlated to $-\log(p)$ of the GO term.

We tested for enrichment between DEGs and neuropsychiatric disorder-associated genes using the R package GeneOverlap (https://bioconductor.org/packages/release/bioc/html/GeneOverlap.html) and an established list of known susceptibility genes of psychiatric disorders.[52] To estimate the age of human brain organoids, we correlated their pseudobulked gene-expression profiles to a transcriptomic dataset, focusing on the developing human cortical tissues (including frontal, parietal, temporal, and occipital cortices) (https://hbatlas.org).[53] Velocity analysis was performed using the scvelo algorithm, implemented in the R package velociraptor.[54] Spliced and unspliced read counts were computed with Velocyto (https://bioconductor.org/packages/release/bioc/html/velociraptor.html) from the Cell Ranger output.[55]

Bulk RNA-seq and RT-qPCR

Cells were washed twice with PBS, dissolved in TRIzol (ThermoFisher), and stored at −80°C. Total RNA was extracted using the phenol-chloroform extraction method, and its integrity was evaluated using Agilent TapeStation 4200 (Agilent Technologies). RNA-seq libraries were prepared using the NEBNext Ultra II RNA Library Prep Kit for Illumina following the manufacturer's instructions (New England Biolabs). In brief, mRNAs were initially enriched with Oligod(T) beads. Enriched mRNAs were fragmented for 15 min at 94°C. First-strand and second-strand cDNA were subsequently synthesized. cDNA fragments were end repaired and adenylated at 3′ ends, and universal adapters were ligated to cDNA fragments, followed by index addition and library enrichment by PCR with limited cycles. The sequencing library was validated on the Agilent TapeStation (Agilent Technologies) and quantified by using a Qubit 2.0 fluorometer (Invitrogen) as well as by qPCR (KAPA Biosystems). The sequencing libraries were multiplexed and clustered onto a flow cell on the Illumina NovaSeq instrument according to the manufacturer's instructions and sequenced using a 150-bp paired-end configuration. Image analysis and base calling were conducted using NovaSeq Control software. Raw sequence data (.bcl files) generated from Illumina NovaSeq were converted into fastq files and de-multiplexed using Illumina bcl2fastq 2.20 software. One mismatch was allowed for index sequence identification. After investigating the quality of the raw data, sequence reads were trimmed to remove possible adapter sequences and nucleotides with poor quality. The trimmed reads were mapped to the reference genome available on ENSEMBL using the STAR aligner v.2.5.2b. The STAR aligner is a splice aligner that detects splice junctions and incorporates them to help align the entire read sequences. This step generated BAM files. Unique gene hit counts were calculated by using the feature Counts from the Subread package v.1.5.2. Only unique reads that fell within exon regions were counted. After the extraction of gene hit counts, the gene hit counts table was used for downstream differential expression analysis. Using DESeq2, a comparison of gene expression between the groups of samples was performed. The Wald test was used to generate p values and log_2 fold changes. Genes with $p_{adj} < 0.1$ were called differentially expressed for each comparison. Due to the presence of 1 male sample (Ctrl-4), Y chromosome genes were removed from downstream analysis. GO analyses were performed on the statistically significant set of genes using g:Profiler.[56]

For Manhattan plots of the chromosome 17p arm, the dataset was filtered to remove genes with low counts across samples (gene count equal to or greater than 10). Technical replicates were collapsed using the collapseReplicates() function provided by DESeq2. Differential expression analysis was performed using the DESeq2 package. Given the primary interest in identifying significantly downregulated genes, a one-sided Wald test was conducted on the dataset using DESeq(), with the null hypothesis that the gene expression in the SMS group is expected to be as high as in the Ctrl group, with the alternative hypothesis being that gene expression was lower in the SMS condition compared to the Ctrl group.

For RT-qPCR, RNA was reverse transcribed using the SuperScript III First-Strand Synthesis System (Thermo Fisher), and qPCR reactions were conducted using SsoAdvanced Universal SYBR Green Supermix (Bio-Rad) in the StepOnePlus real-time PCR system (Applied Biosystems). *GAPDH* was used as a housekeeping control. Primer sequences are in Table S4.

Immunocytochemistry

ICC experiments were performed as described previously.[57–59] In brief, 2D cells (hiPSCs, NPCs, and cortical neurons) were plated on Matrigel-coated or PLO/laminin-coated coverslips, fixed with 4% paraformaldehyde in PBS for 15 min, permeabilized with 0.1% Triton X-100 in PBS, and blocked in 2% normal donkey serum (NDS)/5% bovine serum albumin (BSA) in PBS for 1 h. Cells were incubated overnight with primary antibodies (see below) in 2% NDS/5% BSA in PBS at 4°C. After several PBS washing steps, cells were incubated with fluorescence-conjugated secondary antibodies (see below) for 1 h at room temperature. After PBS washing, cells were mounted onto glass slides with DAPI Fluoromount-G (Southern Biotech). Image acquisition was performed on a confocal microscope (Olympus FV-1000 confocal laser scanning microscope) or an epifluorescence microscopy.

Organoids were fixed with 4% paraformaldehyde in PBS at 4°C overnight, washed with PBS, transferred to a 30% sucrose solution in PBS, and incubated at 4°C overnight. Organoids were then embedded in an OCT medium, snap frozen in dry ice, and stored at −80°C. Frozen organoids were sectioned 10–12 μm thick at −16°C to −20°C in a cryostat and applied to positively charged slides, which were dried at room temperature and stored at −80°C. ICC was performed as described above.

Primary antibodies used in this study for ICC are as follows: NANOG (Abcam, ab109250, dilution 1:250); SSEA4 (Abcam,

ab16287, dilution 1:100); OCT4 (Abcam, ab19857, dilution 1:100); PAX6 (BioLegend, #901302, clone Poly19013, dilution 1:1,000–2,000); Nestin (MilliporeSigma, MAB5326, clone 10C2, dilution 1:250); SOX2 (Abcam, ab97959, dilution 1:100); phospho-histone H2A.X (Ser139) (Cell Signaling, #80312, clone D7T2V, dilution 1:400); phospho-53BP1 (Ser1778) (Cell Signaling, #2675, dilution 1:100); GFP (Abcam, ab13970, dilution 1:250); PSD95 (Abcam, ab2723, clone 6G6-1C9, dilution 1:500); VGLUT1 (MilliporeSigma, AB5905, dilution 1:2,500); SYNAPSIN-1 (Abcam, ab254349, dilution 1:500); MAP2 (Abcam, ab5392, dilution 1:5,000); NEUN (Abcam, ab177487, clone EPR12763, dilution 1:300); CaMKIIα (Abcam, ab22609, clone 6G9, dilution 1:500); Ki67 (R&D Systems, AF7649, dilution 1:100–200); and Ki67 (Sigma, MAB4190, clone Ki-S5, dilution 1:500).

Secondary antibodies used in this study include Cy3 AffiniPure donkey anti-rabbit immunoglobulin G (IgG) (H + L) (Jackson ImmunoResearch, #711-165-152, dilution 1:2,000); Cy3 AffiniPure donkey anti-guinea pig IgG (H + L) (Jackson ImmunoResearch, #706-165-148, dilution 1:2,000); fluorescein isothiocyanate (FITC) AffiniPure $F(ab')_2$ fragment donkey anti-chicken IgY (IgG) (H + L) (Jackson ImmunoResearch, #703-096-155, dilution 1:2,000); Alexa Fluor 647 AffiniPure donkey anti-mouse IgG (H + L) (Jackson ImmunoResearch, #715-605-150, dilution 1:2,000); Cy3 AffiniPure donkey anti-chicken IgY (IgG) (H + L) (Jackson ImmunoResearch, #703-165-155, dilution 1:2,000), and FITC AffiniPure donkey anti-mouse IgG (H + L) (Jackson ImmunoResearch, #715-095-151, dilution 1:2,000).

Imaging analysis

The number of fields of view per slide or section, and the number of slides or sections per sample, are indicated in Table S5. For neuronal morphometric analyses, forebrain neurons at two different time points (3 weeks post differentiation [WPD] and 7 WPD) were sparsely infected with a lentivirus that delivers GFP to myristoylation sites on the cell membrane (myrGFP, MOI = 0.01). Neurons were fixed with 4% paraformaldehyde 7 days after lentiviral infection (4 WPD and 8 WPD). GFP signal was enhanced using ICC, after which the slides were imaged on a confocal microscope with a 1,024 × 1,024 resolution at 10× magnification. z stacks were taken with a step size of 2.0–3.5 μm. The neurons were individually reconstructed in 3D using user-guided tracing with Neurolucida 360 software as described previously[60] (MBF Biosciences, version 2020.3.3). Neurons for reconstruction had the following features: (1) pyramidal or ovoid-shaped somas, (2) at least two branched neurites, and (3) all neurites visible in the GFP signal. Branched structure analysis (soma volume and total length of dendrite per neuron) and spatial analysis (Sholl analysis, critical radius, and maximum crossings) were analyzed using Neurolucida Explorer (MBF Biosciences). For Sholl analyses, 10-μm increments defined the gradually increasing radius of concentric circles centering at the centroid of the soma. To quantify excitatory synapse density, cortical neurons were stained for MAP2, PSD95, and VGLUT1 or SYNAPSIN-1 antibodies as described above for ICC. Confocal images were taken on an Olympus microscope at 60× magnification using a 0.3- to 1.0-μm step size. Images were then analyzed using ImageJ (Fiji v.2.3.0) software. The number of puncta ($VGLUT1^+PSD95^+$ and $SYNAPSIN\text{-}1^+PSD95^+$) was manually and blindly counted to sample identities. MAP2 signals were used to confirm the dendritic location, and only puncta contacting the dendrites were counted. Confocal or epifluorescence microscopy images were taken at 20× for quantification and representative images of hiPSC markers (step size = 3 μm), and at 10× for quantification and 40× for representative images of NPC markers (step size = 2.5–3 μm). NEUN and CAMKIIα confocal images were taken at 10× for quantification (step size = 3 μm) and 40× for representative images (step size = 1 μm). Ki67 confocal images in NPCs were taken at 40× for quantification and representative images (step size = 2 μm). For γH2AX and p-53BP1 foci, confocal images were taken at 60× (step size = 1 μm). All images were analyzed using ImageJ. Quantification and representative images of organoids were taken by a confocal microscope or an epifluorescence microscope, and images were analyzed using ImageJ or Neurolucida360 studio.

Patch-clamp electrophysiology

Whole-cell patch-clamp recordings were obtained using 3–5 MΩ pipettes filled with an internal solution that contained 131 mM potassium gluconate, 8 mM NaCl, 20 mM KCl, 2 mM EGTA, 10 mM HEPES, 2 mM MgATP, and 0.3 mM Na_3GTP (pH 7.2, with KOH, ~280 mOsm with sucrose) in carbogenated BrainPhys medium (~290 mOsm). Signals were recorded with a 5× gain, low-pass filter at 2 kHz, digitized at 10 kHz (Molecular Devices Multiclamp 700B), and analyzed with pClamp 11 (Molecular Devices). After breaking the membrane seal, cells were first voltage clamped (VC) at −70 mV, and a test pulse of −5 mV was applied to measure passive cell properties (membrane resistance, access resistance, and capacitance). Spontaneous synaptic events mediated by AMPA receptors were recorded for at least 3 min at −70 mV (close to the Cl^- reversal potential). In voltage-clamp (V_{clamp}) mode, cells were maintained at a holding potential of −70 mV and then were subjected to 500-ms depolarizations in 10-mV steps from −90 mV to +50 mV to analyze voltage-gated Na^+ and K^+ components. In current-clamp (I_{clamp}) mode, a small current was injected to maintain resting membrane potential at −70 mV. Current steps (10 pA increments, 500 ms) from hyperpolarized (−110 pA) to polarized (50 mV) steps were applied to evoke action potentials (APs) for classification of neuronal maturity according to firing patterns.[61] The AP properties of types IV/V mature neurons (sodium component at V_{clamp} and multi-AP firing at I_{clamp}) were further analyzed. We analyzed AP properties using the first APs elicited by I_{ramp}, and voltage was held at −60 mV at gap-free mode. For miniature current recordings, the internal solution contained 130 mM gluconate acid, 8 mM CsCl, 1 mM NaCl, 2 mM EGTA, 0.8 mM CsOH, 10 mM HEPES, 2 mM MgATP, and 0.3 mM Na_3GTP (pH 7.2, with CsOH, 290–300 mOsm with sucrose). Miniature excitatory postsynaptic currents (mEPSCs) were recorded in the presence of 1 μM tetrodotoxin (TTX) at a holding potential of −70 mV. Baseline mEPSCs were recorded within 3 h in the acute TTX bath. For synaptic scaling experiments, 1 mM TTX was added to the culture medium 24 h before mEPSC recording. The mEPSCs were analyzed from the template-detected events (with amplitude above noise) from at least 2 min of continuous recordings that contained >12 events/cell (Molecular Devices, Clampfit 11). Synaptic events with amplitude greater than 15 pA were selected for averaged amplitude and cumulative probability analysis.

Statistical analyses

The statistical analysis methods for Hi-C, bulk RNA-seq, and snRNA-seq are described in the corresponding sections. The rest

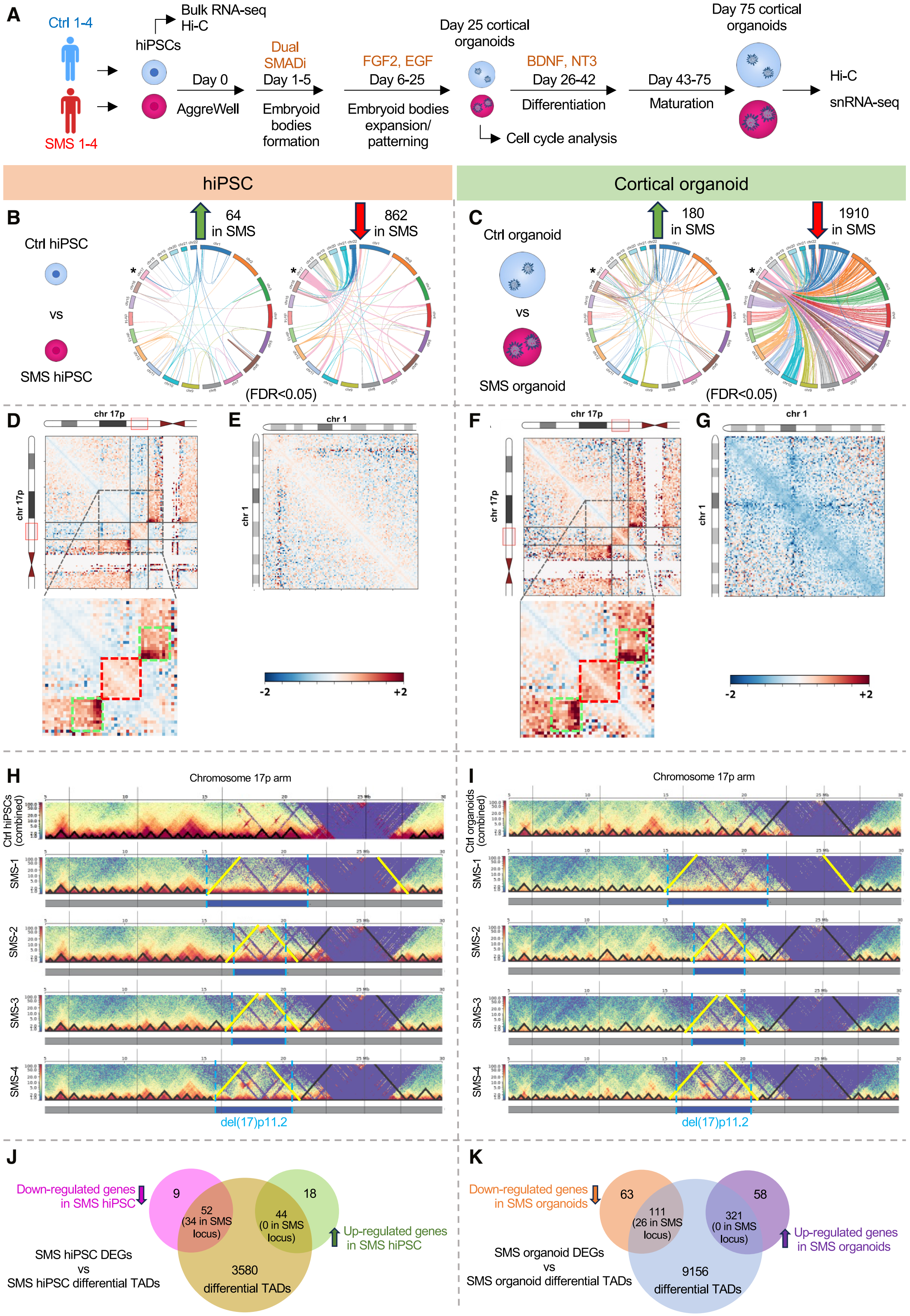

Figure 1. del(17)p11.2 alters global and local chromatin contacts in SMS hiPSCs and hiPSC-derived cortical organoids

(A) Experimental overview. Control (Ctrl) and SMS hiPSCs were differentiated into patterned cortical organoids by exposing them to growth factors and allowing them to mature for up to 75 days.

(B) Circos plots showing statistically significant (FDR < 0.05) increase (left) and decrease (right) of genome-wide intra- and inter-chromosomal contacts in SMS hiPSCs (SMS 1–4) versus Ctrl hiPSCs (Ctrl 1–4). Each line represents a differential chromosomal contact change. Asterisks indicate chromosome 17.

(legend continued on next page)

of the data were analyzed for statistical significance using GraphPad Prism 9.0 software. Error bars in plots represent the standard error of the mean (SEM). The sample size and statistical tests used for each analysis are indicated in the text and figure legends. For datasets that meet the assumptions of parametric testing, unless specified, a two-tailed Student's t test with Welch's correction was used, with the df, t, and p values reported in figure legends. For datasets that do not meet the assumptions of parametric testing, an unpaired Mann-Whitney test was performed, with the U and p values reported in the figure legends. For chi-squared analysis, the chi-squared values, degrees of freedom (df), and p values are reported in the figure legends. For data with more than two groups, analysis of variance (ANOVA) was used, with the corresponding F statistic reported in the figure legends. The differences were considered statistically significant as follows: $*p < 0.05$, $**p < 0.01$, $***p < 0.001$, and $****p < 0.0001$.

Results

Transcriptional deficits in SMS hiPSCs

90% of individuals with SMS carry del(17)p11.2.[1,8] Therefore, we used four hiPSC lines derived from individuals clinically diagnosed with SMS who carry heterozygous del(17)p11.2 (SMS 1–4) and four control hiPSC lines from unrelated healthy noncarriers (Ctrl 1–4) (Figure S1A; Tables S1 and S2). We derived all SMS hiPSCs from female individuals, given the predominance of autistic features in women with SMS.[3] We first characterized the hiPSC lines by verifying the del(17)p11.2 deletion and showing that both Ctrl and SMS hiPSCs have high pluripotency marker expression and expected del(17) p11.2 chromosomal G-banding patterns in the SMS lines (Figures S1B–S1G). Bulk RNA-seq confirmed that genes located within del(17)p11.2 were significantly downregulated in SMS hiPSCs (Figure S2A), indicating that del(17) p11.2 has a *cis* effect on the expression of genes located within the SMS-deleted locus. Globally, DEG analysis identified 61 downregulated genes, including 33 del(17) p11.2 genes and 62 upregulated genes in SMS hiPSCs (Figure S2B). The majority of DEGs (73%) in SMS hiPSCs were outside of del(17)p11.2, suggesting a *trans* effect of del(17)p11.2 on gene expression. In SMS hiPSCs, GO analysis found an enrichment of downregulated genes that mediate cell export; in contrast, genes upregulated in SMS hiPSCs were most enriched for cell-adhesion molecules, including members of the protocadherin family (Figures S2C and S2D, with RT-qPCR validation of selective protocadherins in Figure S2E). These data indicate that del(17)p11.2 has a *cis* effect on the expression of genes within the SMS locus and *trans* effects on non-del (17)p11.2 genes.

del(17)p11.2 alters local and global 3D chromatin topology in SMS hiPSCs and cortical organoids

Studies using non-brain tissues have found that copy-number variants (CNVs) associated with developmental disorders can lead to rearrangements of 3D genome organizations.[62,63] We were thus interested in understanding how del(17)p11.2 impacts the contact maps of human genomic structures in hiPSCs and neural tissues. We performed chromosomal conformational capture (Hi-C) analysis using hiPSCs (lines Ctrl 1–4 and SMS 1–4) and hiPSC-derived day-75 cortical organoids[36] (lines Ctrl 2–4 and SMS 1–4) (Figure 1A). Hi-C identified a total of 6.43 billion unique reads, among which 3.1 billion were high-quality read pairs used for downstream analyses (Figures S3A–S3H). Both Ctrl and SMS cortical organoids had a significantly increased fraction of interchromosomal *trans* interactions and a decreased fraction of intrachromosomal *cis* interactions compared to corresponding hiPSCs (Figures S3I and S3J).

To explore how del(17)p11.2 impacts genome-wide intra- and inter-chromosomal contacts, we compared developmental-stage-matched Ctrl and SMS lines and found that SMS hiPSCs gained 64 and lost 862 chromosomal contacts compared to Ctrl hiPSCs (FDR < 0.05, Figure 1B). Moreover, SMS cortical organoids gained 180 chromosomal contacts and lost 1,910 chromosomal contacts compared to Ctrl organoids (FDR < 0.05, Figure 1C). The majority of decreased chromosomal *trans* contacts in SMS hiPSC (59.2%) and SMS organoids (65.2%) involved chromosome 17. This analysis indicated that genome-wide

(C) Circos plots exhibiting statistically significant (FDR < 0.05) increase (left) and decrease (right) of genome-wide intra- and inter-chromosomal contacts in SMS cortical organoids (SMS 1–4) versus Ctrl cortical organoids (Ctrl 2–4). Each line represents a differential chromosomal contact change. Asterisks indicate chromosome 17.
(D–G) Hi-C heatmaps showing intra- or inter-chromosomal contact frequency in SMS hiPSCs (SMS-2 as an example) versus Ctrl hiPSCs (1–4 combined) (D and E) or SMS-2 cortical organoids versus Ctrl cortical organoids (2–4 combined) (F and G). Each pixel represents one 250-kb region.
(D) Centromeres of chromosome 17 are indicated by red triangles; red solid boxes and black lines indicate the boundaries of del(17) p11.2 (the SMS locus). The SMS locus and its surrounding regions (dashed square) are magnified at the bottom, with the red dashed box indicating increased contacts within del(17)p11.2 and the green dashed boxes indicating increased contacts between the SMS locus-flanking regions in SMS hiPSCs. The color scale goes from −2 (blue) to 0 (white) to +2 (red).
(E) Heatmap showing that chromosome 1 lacks the changes in intra-chromosomal contacts observed in chromosome 17.
(F and G) Similar patterns of chromosomal contact changes were found in SMS cortical organoids.
(H and I) Topological domains on chromosome 17 in Ctrl hiPSCs (1–4 combined) and SMS 1–4 hiPSCs (individually shown) (H) and Ctrl cortical organoids (2–4 combined) and SMS 1–4 cortical organoids (individually shown) (I). Black triangles indicate TADs. Note that the newly formed large TADs in most SMS lines (indicated by yellow lines) encompassed del(17)p11.2 boundaries, which are indicated by blue boxes and dashed lines.
(J and K) Venn diagrams highlight that differential TADs are associated with a significant subset of DEGs identified in SMS hiPSCs (J) and cortical organoids (K).

miswiring of chromosomal contacts induced by del(17) p11.2 became more severe during corticogenesis. Therefore, we focused our analysis on the chromosome 17 p-arm and found that the *cis* chromosomal contacts within del(17)p11.2 deletion boundaries were increased in SMS hiPSCs compared to Ctrl hiPSCs (Figures 1D and S4A). This pattern was consistently observed on chromosome 17 but not on other autosomes in all SMS hiPSC lines (chromosome 1 as an example, Figure 1E). As expected, the chromosomal regions flanking del(17)p11.2 in SMS hiPSCs showed increased chromosomal contacts (Figures 1D and S4A), confirming that the chromosomal regions on either side of the del(17)p11.2 breakpoints were brought into close proximity due to the deletion. The same pattern persisted during corticogenesis, as evidenced by analysis in SMS cortical organoids (Figures 1F, 1G, and S4B). These data indicate that the chromosomal regions within and immediately flanking the SMS locus showed increased chromosomal *cis* contacts in SMS hiPSCs and SMS cortical organoids.

The genome is spatially partitioned into the A compartments displaced in the interior of the nucleus and the B compartments that lie on the nuclear periphery.[64] We analyzed genome-wide A/B compartment classification and observed no differences in the A/B compartments between Ctrl and SMS samples at either developmental stage (Figures S4C and S4D). The A/B compartments comprise a series of self-interacting and cell-type-invariant structural units called TADs.[65] The average sizes of TADs for each chromosome did not differ between Ctrl and SMS tissues (Figure S5). Focusing on the chromosome 17 p-arm, we found that del(17)p11.2 induced TAD fusion in all SMS hiPSCs and organoids. Specifically, in Ctrl hiPSCs and organoids, the SMS locus was partitioned into a dozen smaller TADs (Figures 1H and 1I). By contrast, the entire del(17)p11.2 region in SMS hiPSCs and organoids was encompassed by a newly emerged large TAD (Figures 1H and 1I). The formation of a single self-interacting TAD in SMS lines is consistent with our analysis that showed increased chromosomal contacts within the del(17)p11.2 boundaries (Figures 1D–1F, S4A, and S4B). Next, we performed genome-wide differential TAD analysis, including alterations in inter- and intra-TAD contacts, and found 3,676 differential TADs in SMS hiPSCs and 9,588 differential TADs in SMS cortical organoids. Differential TADs could underlie gene dysregulation.[66,67] Therefore, we compared the differential TADs in SMS hiPSCs with the DEGs identified in SMS hiPSCs. Among the 123 uniquely annotated DEGs in SMS hiPSCs, 34 del(17)p11.2 genes and 62 non-del(17)p11.2 genes (including 18 downregulated and 44 upregulated genes) were associated with differential TADs in SMS hiPSCs (Figure 1J). The overlap between DEGs and differential TADs was highly significant ($p = 1.22 \times 10^{-10}$, odds ratio = 3.59), indicating a strong enrichment of DEGs within differential TADs. By contrast, among 553 uniquely annotated DEGs in SMS cortical organoids (at day 75 of differentiation, see below for details), 26 del(17)p11.2 genes and 406 non-del(17)p11.2 genes (including 85 downregulated and 321 upregulated genes) were affiliated with differential TADs in SMS organoids (Figure 1K) with no significant enrichment detected. We further performed a chi-squared test, which confirmed the statistical significance for hiPSC data (4.12×10^{-10}) and a weak dependence in cortical organoid data (7.75×10^{-6}). Therefore, differential TADs in SMS hiPSCs and SMS organoids could underlie 70% (62/89) and 77% (406/527) of non-del(17)p11.2 DEGs, respectively. Collectively, these data show that in SMS cells, the flanking regions of the SMS locus have increased contacts that incur the fusion of small topological domains separating different del(17)p11.2 genes into a single TAD encompassing the entire SMS locus, potentially altering the gene-regulatory landscape of del(17)p11.2 genes. Beyond the SMS locus, differential TADs identified in SMS hiPSCs were significantly enriched for non-del(17)p11.2 DEGs, indicating a contribution of chromatin topological reorganization in gene dysregulation.

del(17)p11.2 alters the expression of genes associated with cell cycle, neurodevelopment, and synapse assembly in SMS hiPSC-derived cortical organoids

To determine how del(17)p11.2 alters cell-type-specific gene-expression programs, we performed snRNA-seq using Ctrl and SMS organoids at day 75 of differentiation, when multiple radial glial cell and neuronal populations coexist. After quality control and data processing, we generated 42,319 high-quality single-nucleus transcriptomes from three Ctrl and three SMS lines, detecting approximately 1,383–3,527 genes per cell (Figures S6A–S6D). To assess the developmental age of cortical organoids relative to the human brain, we compared the pseudobulked snRNA-seq data to published developing human neocortex transcriptomes.[53] We found that day 75 cortical organoids correlated best with human neocortex at the 17th week post conception (Figure 2A), indicating that the organoids transcriptionally model mid-gestation stages of human telencephalic development.

In line with recent studies of hiPSC-derived cortical organoids,[28,68,69] we identified 16 transcriptionally distinct cell clusters (Figures 2B and 2C). These clusters include populations of radial glial cells such as the *TOP2A*$^+$*MIK67*$^+$ cycling radial glia (cycling RG), two groups of *HOPX*$^+$ outer radial glia (oRG), *S100B*$^+$*NTRK2*$^+$ astroglia, *VIM*$^+$*TNC*$^+$ RG-like cells, *CRYAB*$^+$ truncating radial glia (tRG) and tRG-like cells, *LHX1*$^+$*CA8*$^+$ migrating neurons (mi-N), *LHX8*$^+$*LHX9*$^+$ immature neurons (im-N), *EOMES*$^+$ intermediate progenitor cells (IPCs), three populations of *DLX6-AS1*$^+$*DLX6*$^+$*DLX5*$^+$ GABAergic neurons, and two populations of *SLC17A7*$^+$*TBR1*$^+$*SATB2*$^+$*FEZF2*$^+$ glutamatergic neurons (Figures 2D, 2E, and S7). Interestingly, cell-type compositions were largely consistent between Ctrl and SMS samples, except for an increased proportion of cycling RGs in SMS organoids (Figure 2F).

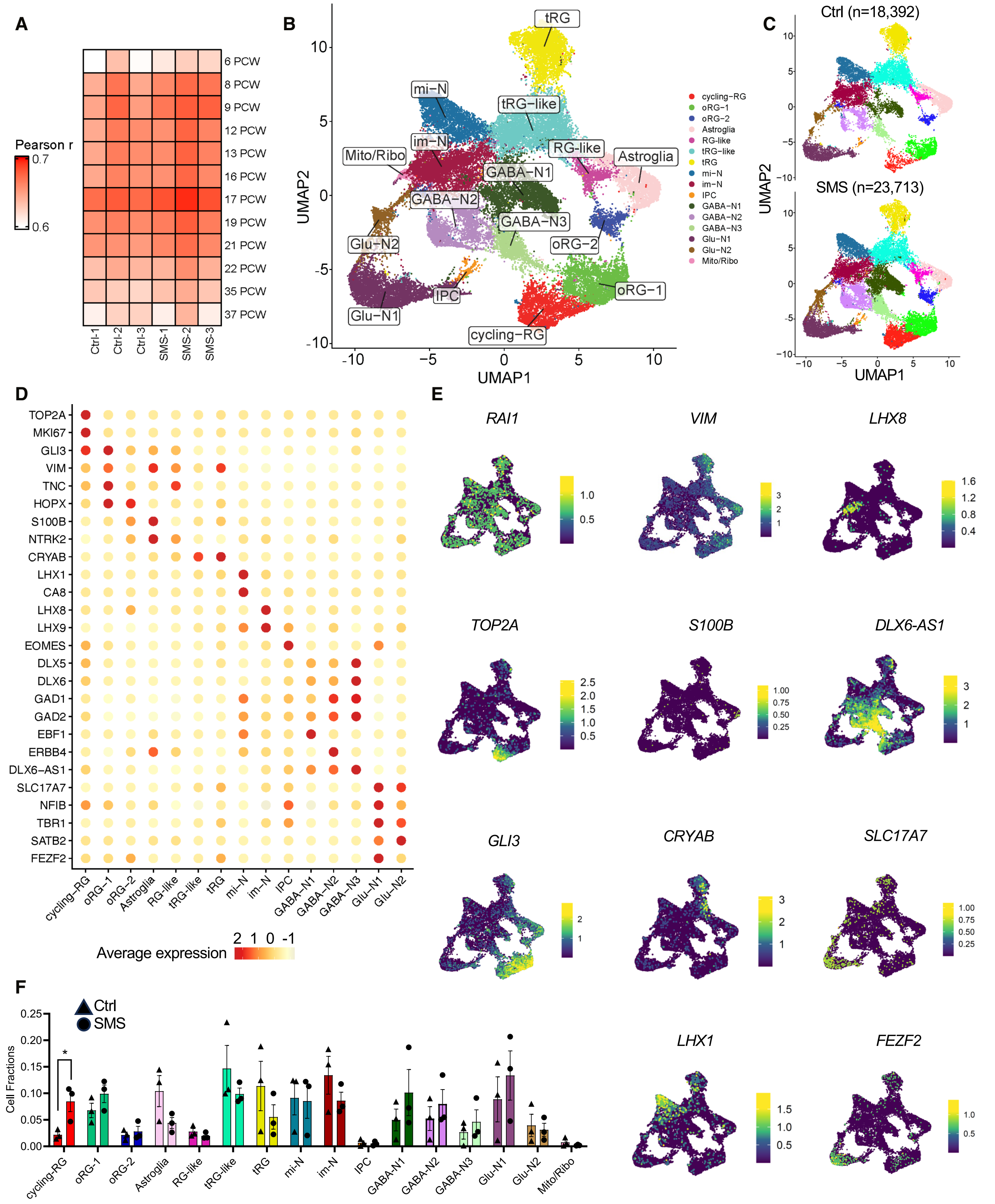

Figure 2. snRNA-seq characterization of Ctrl and SMS cortical organoid transcriptomes

(A) Classification of cortical organoids against a developing human cortex transcriptome dataset.[53] The snRNA-seq samples were pseudobulked, and the correlation coefficients (Pearson's r) were color coded. Data obtained from Ctrl 1–3 and SMS 1–3 day-75 organoids (one library for each line, 3–5 cortical organoids per line).

(B) Uniform manifold approximation and projection (UMAP) visualization showing different cell clusters, including the cycling radial glia (cycling RG), outer radial glia (oRG), truncating radial glia (tRG), intermediate progenitor cells (IPC), migrating neurons (mi-N), immature neurons (im-N), glutamatergic neurons, and GABAergic neurons.

(C) UMAP projection of cell clusters among Ctrl (n = 18,392) and SMS (n = 23,713) lines. Individual samples are shown in Figure S6A.

(legend continued on next page)

A small group of cells expressing mitochondria genes and ribosomal genes (Mito/Ribo) were excluded from downstream analyses. Collectively, initial analysis of snRNA-seq data showed that del(17)p11.2 could impact multiple cell types during early cortical development, especially the highly proliferative cycling RG.

To identify cell-type-specific transcriptional alterations in SMS cortical organoids, we performed pseudobulk DEG analysis for all cell clusters. Our previous work found that SMS mouse models show modest gene-expression changes,[14,16,17,70] similar to those reported in the mouse model of Rett syndrome (MIM: 312750).[71] Therefore, we applied a relatively permissive threshold ($p_{adj} < 0.2$) for the purpose of gene discovery, as reported in similar studies.[72,73] DEG analysis found 938 combined DEGs across all cell clusters, including *RAI1* and other del(17)p11.2 genes (Figures 3A–3D). To determine how del(17)11.2 affects gene expression in *trans*, we removed del(17)11.2 genes and compared overlapping DEGs across all cell clusters. Downregulated genes in SMS organoids were more commonly shared among different cell clusters. In contrast, upregulated genes in SMS organoids were more specific to each cell type, and this trend persisted when DEGs were identified using a more stringent FDR (Figures S8A and S8B). Notably, multiple SMS cell clusters shared downregulation of *POTEI* and *POTEF*, members of a primate-specific *POTE* gene family that contain ankyrin and spectrin repeats and could mediate cell membrane interactions[74] (Figure 3E). DEG patterns were most pronounced in the tRG, mi-N, and glutamatergic neurons (Figure S8C). To determine the overall patterns and enriched functional categories of DEGs from all cell clusters, we visualized the enriched GO structure and hierarchy. GO over-representation analysis of all 938 DEGs (including del(17)p11.2 genes) from all cell clusters demonstrated that SMS organoids showed downregulation of genes in triglyceride catabolic (*CPS1* and *PNPLA3*) and alcohol biosynthetic (*HMGCS1*, *MVD* and *LSS*) processes, prominently in mi-N (Figures 3F and S8D). By contrast, upregulated genes in SMS organoids were enriched for negative regulators of DNA transcription in tRG (*ASCL1*, *AKR1B1*, and *GADD45A*) and glutamatergic neurons (*NFIB*, *RORβ*, and *SMARCA2*) and cell-cycle regulators in tRG (*CCNB1* and *INSM1*) (Figures 3F and S8D). To more fully assess how biological functions and pathways were altered, we considered the levels of all expressed genes and performed gene set enrichment analysis (GSEA), which does not rely on cutoffs to define DEGs. Notably, oRG-1, oRG-2, tRG, mi-N, im-N, GABA-N2, and GABA-N3 showed upregulation of genes involved in cell-cycle regulation processes, whereas synaptic signaling genes were upregulated in astroglia and Glu-N1 clusters (Figures 3G and S9). By contrast, organelle assembly and metabolic and biosynthetic processes were downregulated in oRG-1, oRG-2, astroglia, RG-like cells, mi-N, and Glu-N1 clusters, whereas the tRG, im-N, IPC, and GABA-N2 clusters showed downregulation of the cilium and microtubule organization pathways (Figure S9).

To determine whether del(17)p11.2 alters neuronal maturation, we costained SOX2 (marks NPCs) and NEUN (marks mature neurons) and found that SMS organoids showed an increased proportion of $NEUN^+SOX2^-$ mature neuronal population, a decreased proportion of $SOX2^+$ neural progenitor population (Figure S10A), and a corresponding increase in NEUN-to-SOX2 ratio (Figure S10B). We then performed scvelo analysis[54] by integrating transcriptomic distance, pseudotime, and splicing information to calculate the latent time (a pseudotime value representing the developmental trajectory, Figure S10C). Our analysis showed that SMS Glu-N1 and Glu-N2 have a higher latent time value than in Ctrl samples. Ctrl samples were more biased toward mi-N and im-N states. This indicates that SMS neurons showed accelerated neuronal maturation. Together, these data collectively indicate that del (17)p11.2 drives accelerated excitatory neuronal maturation and impacts cell-type-specific gene networks, driving downregulation of genes involved in metabolic and organelle assembly pathways and upregulation of genes related to the cell cycle and synaptic signaling.

Individuals with SMS are commonly diagnosed with ASD features[2] and show neuropsychiatric features of bipolar mood and depressive disorder.[75] To determine whether del(17)p11.2-dependent DEGs are associated with ASDs and neuropsychiatric disorder gene signatures, we performed an enrichment test using a published list of misregulated genes in ASD, bipolar disorder (BP), schizophrenia (SCZ), and major depressive disorder (MDD).[52] Interestingly, among downregulated genes in SMS samples, we found several cell clusters enriched for either SCZ- or

(D) Dotplot of feature expression. *TOP2A* and *MKI67* mark cycling RG; *GLI3*, *VIM*, and *TNC* are expressed in radial glial lineages; *HOPX* marks outer radial glia (oRG-1 and oRG-2); *S100β* and *NTRK2* are enriched in astroglia; tRG and tRG-like cells express *CRYAB*; *LHX1* and *CA8* are expressed in migrating neurons (mi-N); *LHX8* and *LHX9* are enriched in immature neurons (im-N); *EOMES* marks intermediate progenitor cells (IPC); *DLX5*, *DLX6*, *GAD1*, and *GAD2* are GABAergic markers; GABA-N1 is enriched with a GABAergic projection neuronal marker *EBF1*; GABA-N2 is enriched with *ERBB4*, important for GABA release; *DLX6-AS1* is expressed in all GABA-N and highest in GABA-N3; *SLC17A7* (*VGLUT1*), *TBR1*, *SATB2*, and *FEZF2* are expressed in glutamatergic neurons (Glu-N); *NFIB* marks corticofugal glutamatergic neurons and regulates the radial-glia-to-IPC transition.
(E) UMAP plots showing the expression of key genes in different cell clusters (combining both genotypes): *RAI1*, expressed in all cell clusters; proliferative marker *TOP2A*; radial glia marker *GLI3* and *VIM*; astroglia marker *S100B*; tRG marker *CRYAB*; migrating neuronal marker *LHX1*; immature neuronal marker *LHX8*; *DLX6-AS1*, expressed in GABAergic neurons (GABA-N); and *SLC17A7* and *FEZF2*, expressed in glutamatergic neurons (Glu-N).
(F) Bar chart showing the fractions of each cell cluster in Ctrl and SMS cortical organoids. SMS samples have a higher fraction of cyclin-RGs, *FDR < 0.05, calculated with a sum-constrained Beta-binomial model.[47]

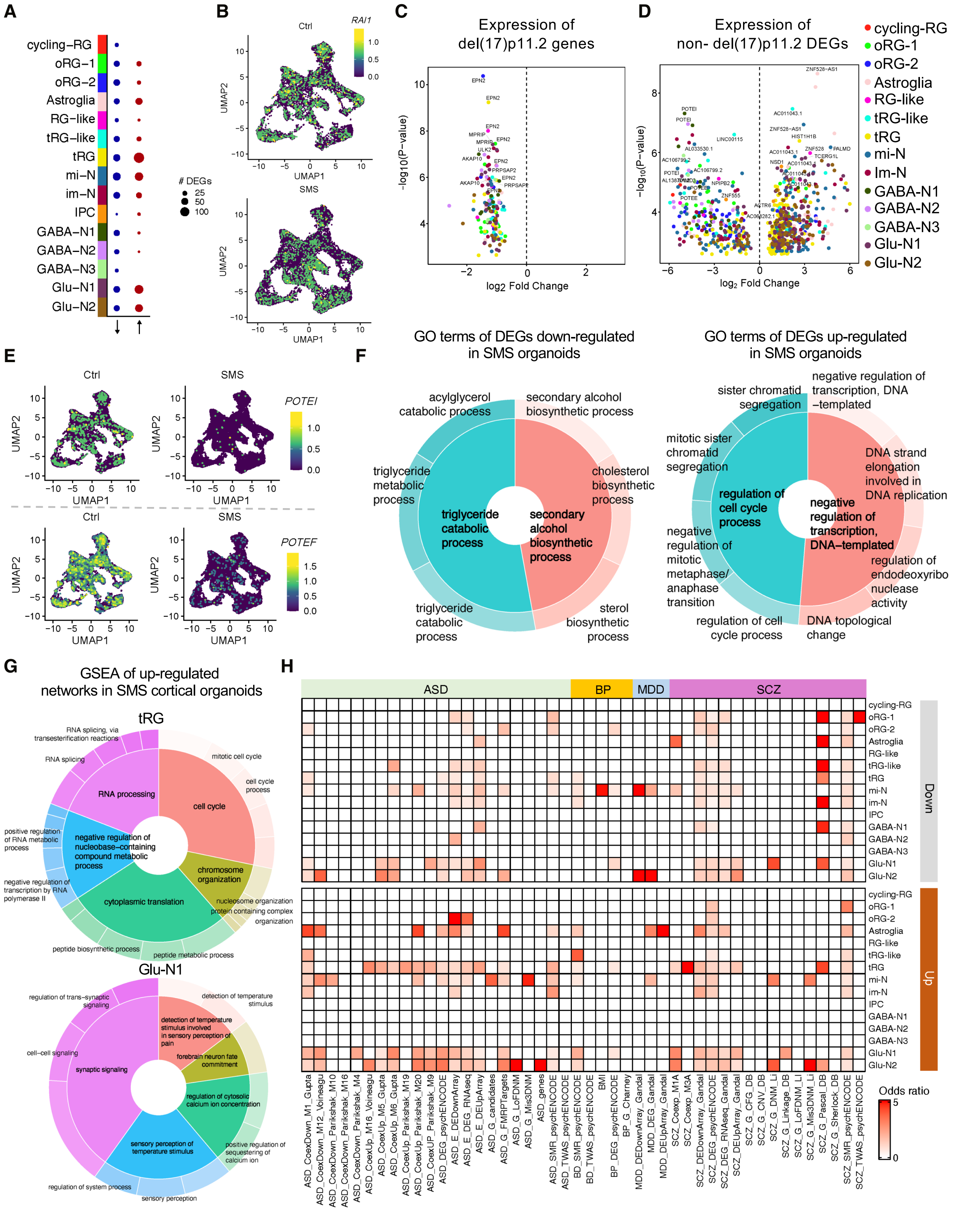

Figure 3. Impaired metabolic, cell-cycle, and neuronal signaling pathways in SMS cortical organoids
(A) Dotplot showing the number of DEGs (indicated by size) in SMS cortical organoid cell clusters.
(B) UMAP plots showing reduced *RAI1* expression in SMS cortical organoid cell clusters.
(C) Volcano plot showing the downregulation of del(17)p11.2 genes in different cell clusters of SMS cortical organoids.
(D) Volcano plot showing the altered expression of non-del(17)p11.2 DEGs in different cell clusters of SMS cortical organoids. DEGs in each cell cluster are shown in Figure S8C.

(legend continued on next page)

MDD-associated genes. Among genes upregulated in SMS, astroglia, tRG, mi-N, and glutamatergic neurons more prominently showed ASD and SCZ gene signatures (FDR < 0.2, Figure 3H; FDR < 0.1, Figure S11). Together, these data indicate that DEGs in SMS cortical organoids share selective transcriptional deficits observed in ASD and SCZ samples, with selective cell types (mi-N, astroglia, and Glu-N) showing an MDD signature.

SMS hiPSC-derived cortical organoids and NPCs exhibit aberrant growth, cell-cycle regulation, and gene expression

To investigate whether the altered 3D chromatin contact map and the transcriptional dysregulation in SMS cortical organoids are associated with neurodevelopmental defects, we monitored the growth of Ctrl and SMS organoids. We divided organoids into three bins by size and found that at day 75 of differentiation, SMS organoids in the largest bin were significantly smaller than age-matched Ctrl organoids (Figure 4A). The dysregulation of cell-cycle-related genes in multiple radial glial cell types suggested that progenitor or differentiation could be impaired. To further investigate, we looked at an earlier time point (day 25 of differentiation), when cortical organoids were enriched with progenitor populations.[36] 25-day-old SMS cortical organoids in the largest bin were also smaller than age-matched Ctrl organoids (Figure 4B). We measured the density and sizes of nuclei in Ctrl and SMS organoids, which revealed a reduction in nuclear size in SMS organoids, despite no changes in nuclear density (Figures S12A and S12B). To evaluate the growth condition of organoids, we measured their circularity (C, with $C = 1$ being a perfect circle) and found that the C values of Ctrl and SMS organoids consistently range between 0.78 and 0.84 at days 25 and 75 of differentiation. SMS organoids showed a small but significant increase in circularity, suggesting a deviation from the typical developmental process (Figures S12C and S12D). These data indicate that SMS organoids exhibited reduced overall growth and smaller nuclear size compared to Ctrl organoids.

To further study the growth dynamics of SMS organoids, we performed immunostaining and found that SMS cortical organoids had enlarged PAX6$^+$ ventricles, both in terms of the absolute mean ventricle sizes (150% increase) and when normalized to the overall sizes of organoids (200% increase, Figures 4C–4E). Further validation found that SMS organoids showed an increased number of ventricles (Figure 4F) and reduced thickness of PAX6$^+$ ventricles (Figure 4G). SMS organoids thus recapitulate mild ventriculomegaly-like features of individuals with SMS.[9,11,12]

Enlarged ventricles and reduced cortical volume could result from impaired NPC progression.[13] Therefore, we examined the short- and long-term proliferative capacity of 25-day-old Ctrl and SMS cortical organoids using EdU pulse-chase experiments (Figure 4H). For the short-term EdU chase, we treated organoids with EdU for 30 min to label cells in the S phase and marked all cycling cells with Ki67. SMS organoids showed a significant decrease in EdU intake and similar proportions of Ki67$^+$ cells compared to Ctrl organoids. Together, this resulted in a significant decrease in the EdU labeling index (EdU$^+$Ki67$^+$/Ki67$^+$ cells) (Figure 4I). For the long-term EdU chase, EdU was treated for 24 h followed by Ki67 staining. We found that SMS organoids had a significantly decreased cell-cycle re-entry index (EdU$^+$Ki67$^+$/EdU$^+$ cells), suggesting that SMS cells exit early from the cell cycle. Correspondingly, SMS organoids had a significantly increased cell-cycle exit index (EdU$^+$Ki67$^-$ cells/EdU$^+$ cells) (Figure 4I). These data indicate that del(17)p11.2 is associated with fewer cells in the S phase and an accelerated cell-cycle exit.

To independently examine how del(17)p11.2 impairs neural progenitor growth in a more homogenous model system than 3D organoids, we differentiated hiPSCs into 2D NPCs (Figure S13A). Ctrl and SMS NPCs expressed a similarly high percentage of NPC markers (Figures S13B–S13E). Bulk RNA-seq confirmed downregulation of del(17)p11.2 genes in SMS NPCs (Figure S14A). DEG analysis found 519 upregulated genes and 874 downregulated genes (including 40 del(17)p11.2 genes) in SMS NPCs (Figure S14B). The majority (97.1%) of DEGs in SMS NPCs were located outside of del(17)p11.2, consistent with a *trans* effect of del(17)p11.2 on gene expression. GO analysis found that genes downregulated in SMS NPCs were involved in protein binding and anatomical structural development and morphogenesis. In contrast, genes upregulated in SMS NPCs participate in nervous system development and neural differentiation (Figure S14C). Several protocadherin genes, including *PCDHA5*, *PCDHGA6*, and *PCDHGA12*, maintained the same dysregulation patterns as in SMS hiPSCs (Figure S14D). Notably, SMS NPCs showed overexpression of *CDKN1C* and underexpression of *CDK18*. Gain-of-function mutations of *CDKN1C* cause the IMAGE syndrome (MIM: 614732) by inducing cell-cycle arrest at G_1,[76] and *CDK18* prevents replication stress and maintains genomic stability (Figure S14E).[77] These data indicate that del(17)p11.2 induces disruption of transcriptional programs important for neural development in 2D NPCs.

(E) UMAP showing the downregulation of primate-specific cell-adhesion-related genes *POTEI* and *POTEF* in multiple cell clusters in SMS cortical organoids.
(F) Nested pie charts showing parent and child GO over-represented terms obtained from DEG analysis ($p_{adj} < 0.05$) by comparing Ctrl and SMS cortical organoids.
(G) Nested pie charts showing upregulated GO terms in SMS tRG and Glu-N1 clusters using gene set enrichment analysis (GSEA).
(H) Heatmap showing over-representation of autism spectrum disorder (ASD), bipolar disorder (BD), major depressive disorder (MDD), and schizophrenia (SCZ) related genes in different cell cluster DEGs (FDR < 0.2). The color of the box shows the odds ratio for enrichment. The odds ratios were calculated by Fisher's exact test.

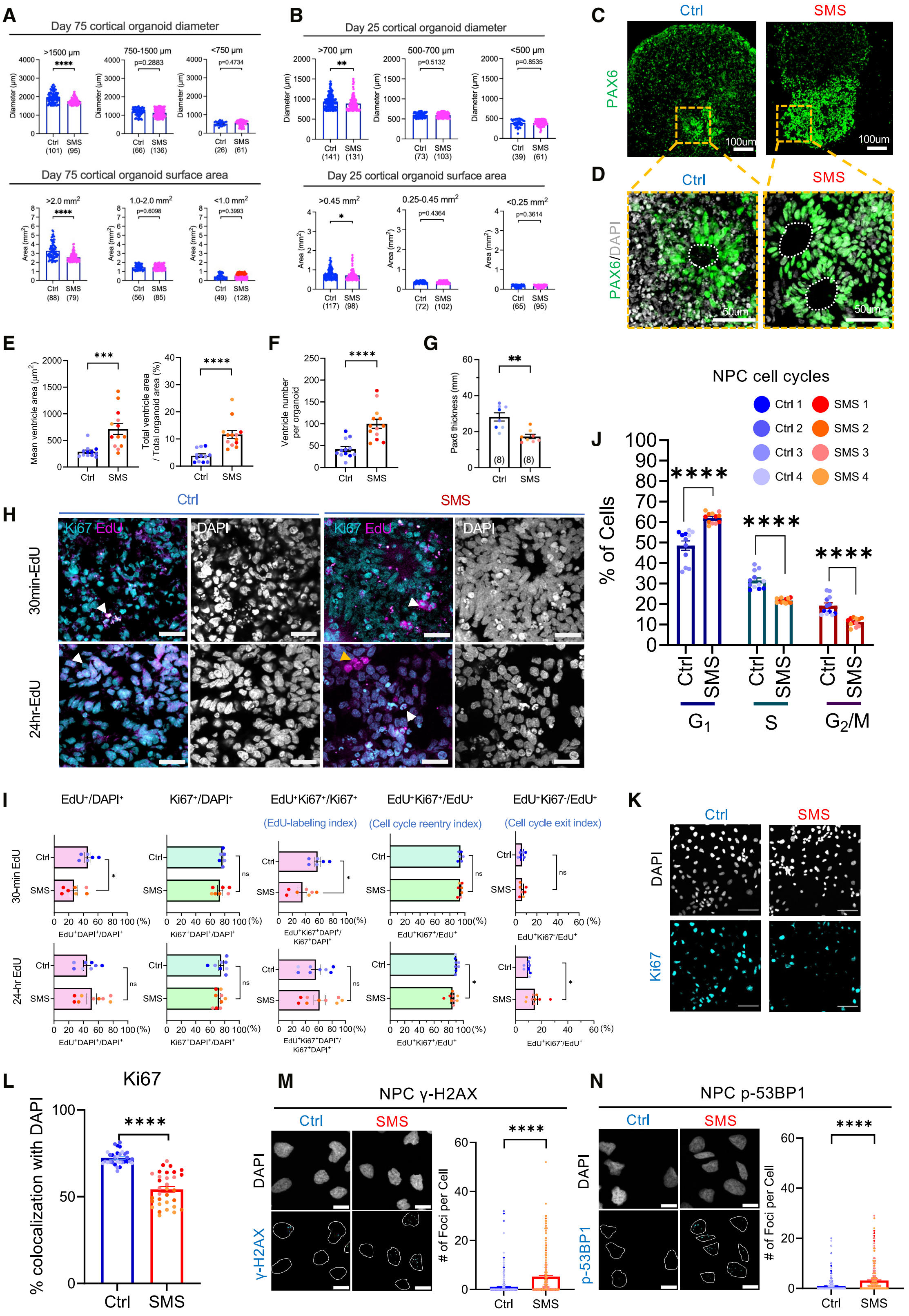

Figure 4. SMS hiPSC-derived neuronal models showed impaired cell-cycle progression and mild ventriculomegaly
(A) Bar graphs of diameter and area of Ctrl and SMS cortical organoids at day 75 of differentiation (divided into three bins by size). SMS organoids in the largest bin were significantly smaller than Ctrl organoids in the same bin. Each dot represents one organoid. Diameter >1,500 μm: $U = 2{,}758$, $p < 0.0001$; area >2 mm^2: $U = 1{,}622$, $p < 0.0001$; two-tailed Mann-Whitney test.

(legend continued on next page)

To determine whether del(17)p11.2 impairs cell-cycle regulation in 2D NPCs, Ctrl and SMS NPCs were treated with propidium iodide followed by flow-cytometry analysis. We found an increased proportion of SMS NPCs in the G_1 phase and a decreased proportion of SMS NPCs in the S and G_2/M phases (Figure 4J), consistent with decreased short-term EdU labeling index in 3D organoids. We found a significantly decreased proportion of SMS NPCs expressing Ki67 (Figures 4K and 4L), consistent with a proliferation defect. Because cell-cycle arrest at the G_1 phase could indicate increased genomic instability,[78] we immunostained for γ-H2AX with phosphorylation at serine 139, a marker for DNA double-strand breaks and replication stress. There was a modest, but significant, increase in γ-H2AX$^+$ foci per cell in SMS NPCs (Figure 4M). Increased DNA damage in SMS NPCs was independently verified with immunostaining for phospho-p53-binding protein 1 (p-53BP1), a sensor for DNA double-strand breaks and replication stress (Figure 4N). DNA damage was not increased in SMS NPC-derived cortical neurons (Figure S15A), suggesting that del(17)p11.2 is associated with genomic instability and/or replication stress in proliferating cells. These data indicate that del(17)p11.2 drives dysregulation of neurodevelopment- and cell-cycle-related genes, increases genomic instability, and reduces proliferative capacity. Furthermore, the reduced organoid size and enlarged ventricles in hiPSC-derived 3D neural models recapitulate disease hallmarks of SMS.

SMS hiPSC-derived neurons showed aberrant gene expression and dendritic growth

To further dissect the impact of del(17)p11.2 on the functional development of cortical neurons, we differentiated NPCs into 2D cortical excitatory neurons. Relative to 3D cortical organoids, hiPSC-derived 2D cortical excitatory neurons are more homogeneous, functionally mature, and accessible to morphometric analysis and electrophysiology recordings.[79] We found a high percentage of Ctrl and SMS-derived cortical neurons expressed postmitotic neuronal marker NEUN (>80%), and >98% of NEUN$^+$ neurons expressed excitatory neuronal marker CAMKIIα (Figure S15B). To determine the functional maturation status of hiPSC-derived cortical neurons, we performed whole-cell patch-clamp electrophysiology. At 9–10 WPD, hiPSC-induced cortical neurons showed resting membrane potentials that hyperpolarize below −50 mV and have characteristics of cortical neurons, including membrane resistance <1 GΩ, action potential half-width (<3 ms), and repetitive AP firing. We then categorized the hiPSC-derived cortical neurons based on their AP patterns using previously established criteria[61] (type I = abortive AP; types

(B) Bar graphs of diameter and area of Ctrl and SMS cortical organoids at day 25 of differentiation (divided into three bins by size). SMS organoids in the largest bin were significantly smaller than Ctrl organoids in the same bin. Each dot represents one organoid. Diameter >700 μm: $U = 7{,}513$, $p = 0.0079$; area: $U = 4{,}756$, $p = 0.0314$; two-tailed Mann-Whitney test.
(C) Representative images showing the overall morphology of Ctrl and SMS organoids at day 25 of differentiation. PAX6 is in green. Orange squares indicate neurogenic zones, which are magnified in (D). Scale bars, 100 μm.
(D) Representative images of PAX6$^+$ ventricles in Ctrl and SMS organoids at day 25 of differentiation. Ventricles are indicated by white dashed lines; PAX6 is in green. Scale bars, 50 μm.
(E) Quantification of PAX6$^+$ ventricle area in Ctrl and SMS organoids, including both absolute size (left) and size when normalized to the total organoid surface area (right). Each dot represents averaged data from one organoid, one image per organoid. Mean ventricle size: $U = 14$, $p = 0.0002$; ventricle size/total organoid area: $U = 5$, $p < 0.0001$; two-tailed Mann-Whitney test.
(F) Quantification found that SMS organoids showed an increased number of PAX6$^+$ ventricles. Each dot represents averaged data from one organoid, one image per organoid. $U = 10.5$, $p < 0.0001$; two-tailed Mann-Whitney test.
(G) Quantification found that SMS organoids showed a decreased thickness of PAX6$^+$ cells around the ventricles. Each dot represents averaged data from one organoid, one image per organoid. $U = 4$, $p = 0.0019$; two-tailed Mann-Whitney test.
(H) Representative images of Ctrl (left) and SMS (right) cortical organoids after a 30-min (top) and 24-h (bottom) EdU pulse. EdU-labeled cells are in magenta, and Ki67-labeled cells are in green. White arrowheads indicate Ki67$^+$EdU$^+$ double-positive cells, and yellow arrowheads indicate Ki67$^-$EdU$^+$ cells. Scale bars, 20 μm.
(I) Top: quantification of the percentages of EdU$^+$ cells ($U = 4$, $p = 0.014$), Ki67$^+$ cells ($U = 16$, $p = 0.5338$), EdU labeling index (EdU$^+$Ki67$^+$ cells/Ki67$^+$ cells, $U = 6$, $p = 0.0315$), cell-cycle re-entry index (EdU$^+$Ki67$^+$ cells/EdU$^+$ cells, $U = 21$, $p > 0.9999$), and cell-cycle exit index (EdU$^+$Ki67$^-$ cells/EdU$^+$ cells, $U = 21$, $p > 0.9999$) after a 30-min EdU pulse. Bottom: quantification of the percentages of EdU$^+$ cells ($U = 27$, $p = 0.4081$), Ki67$^+$ cells ($U = 23$, $p = 0.2268$), EdU labeling index (EdU$^+$Ki67$^+$ cells/Ki67$^+$ cells, $U = 31$, $p = 0.6520$), cell-cycle re-entry index (EdU$^+$Ki67$^+$ cells/EdU$^+$ cells, $U = 12$, $p = 0.019$), and cell-cycle exit index (EdU$^+$Ki67$^-$ cells/EdU$^+$ cells, $U = 12$, $p = 0.019$) after a 24-h EdU pulse. Each dot represents averaged data from one organoid, 1–3 images per organoid. U and p values by two-tailed Mann-Whitney test.
(J) Barplot of cell-cycle profile of SMS and Ctrl NPCs. Three biological replicates per cell line; each dot represents one sample with 4,000–20,000 cells. G_1 phase: $t = 43.99$, df = 11, $p < 0.0001$; S phase: $t = 43.40$, df = 11, $p < 0.0001$; G_2/M phases: $t = 23.41$, df = 11, $p < 0.0001$; one-sample t test.
(K) Representative images of Ctrl and SMS NPCs immunostained for Ki67 (cyan). Scale bars, 50 μm.
(L) Quantification showing a lower percentage of SMS NPCs expressing Ki67. $n = 32$ images per genotype with eight images per cell line; each point represents one image colored based on the cell line. $U = 23$, $p < 0.0001$, two-tailed Mann-Whitney tests.
(M) Expression of γ-H2AX, a DNA-damage marker, in SMS and Ctrl NPCs. Left: representative confocal images of γ-H2AX (cyan) staining in Ctrl and SMS NPCs. Bottom left: outlined nuclei for γ-H2AX quantification. Scale bars, 10 μm. Right: Quantification of γ-H2AX foci per cell in SMS and Ctrl NPCs. $n = 400$ cells per genotype with 100 cells per cell line. Each dot represents one cell. $t = 9.065$, df = 798, $p < 0.0001$; unpaired t test.
(N) Expression of p-53BP1 in SMS and Ctrl NPCs. Left: representative images of p-53BP1 (cyan) staining in Ctrl and SMS NPCs. Bottom left: outlined nuclei for p-53BP1 quantification. Scale bars, 10 μm. Right: quantification of p-53BP1 foci per cell in SMS and Ctrl NPCs. $n = 400$ cells per genotype with 100 cells per cell line. Each dot represents one cell. $t = 7.964$, df = 798, $p < 0.0001$; unpaired t test.

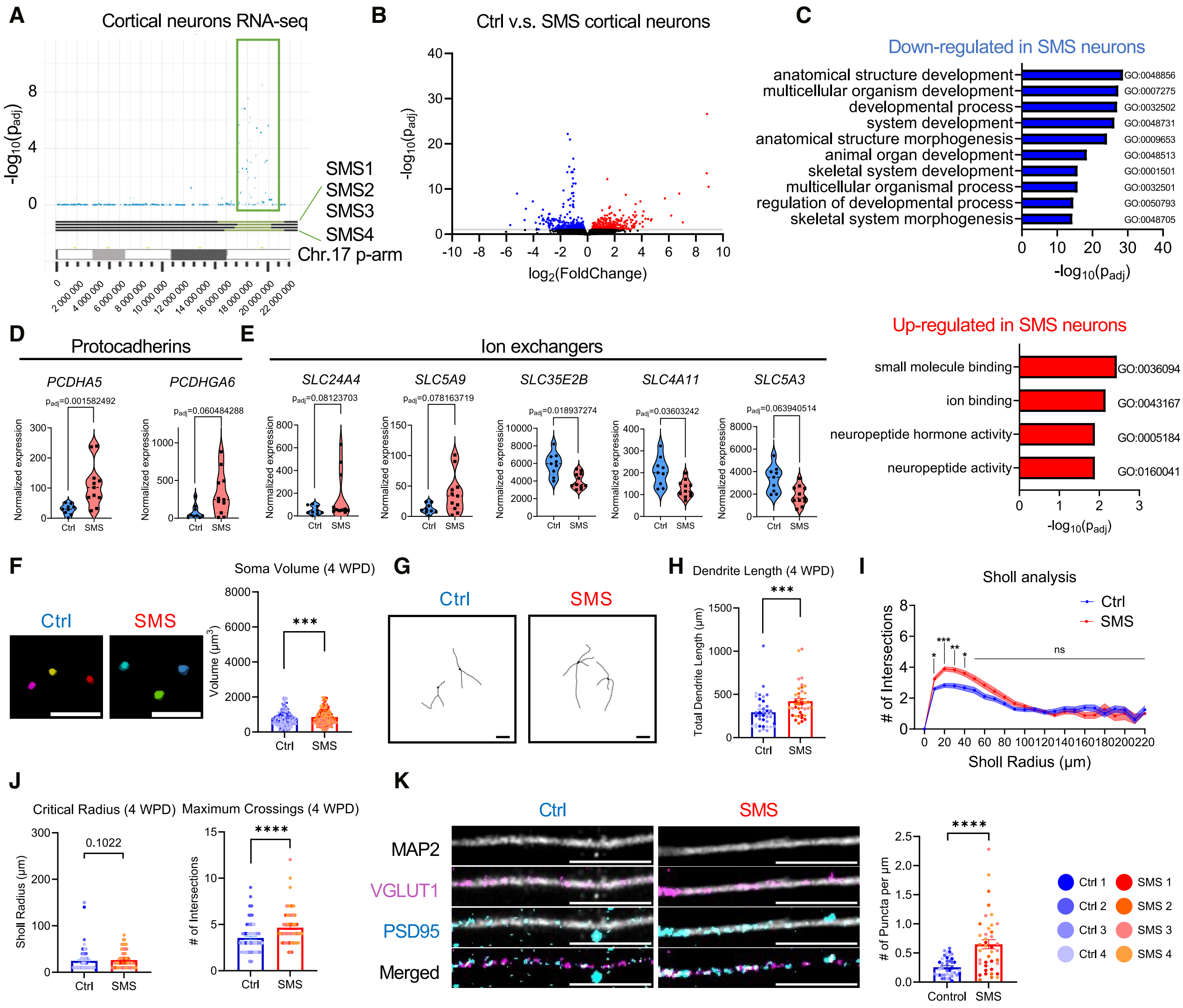

Figure 5. SMS hiPSC-derived cortical neurons showed aberrant gene expression and dendritic growth

(A) Manhattan plot displaying the genomic landscape of significantly downregulated genes on the chromosome 17 p-arm in SMS cortical neurons (compared to Ctrl neurons) based on $-\log_{10}$ transformed p_{adj} derived from a one-sided Wald test. The x axis represents the genomic position, and the y axis corresponds to the significance level of differential expression. The blue dots represent individual genes, with their positions on the x axis representing their location on chromosome 17p and their heights on the y axis indicating the significance of their differential expressions. The dark horizontal lines at the bottom indicate chromosomes, with the green segments corresponding to the regions subjected to del(17)p11.2 in SMS cortical neurons.

(B) Volcano plot showing the global transcriptomic changes by comparing SMS with Ctrl cortical neurons. Each dot represents a gene. The $\log_2$ fold change of each gene is represented on the x axis, and the $-\log_{10}$ of its p_{adj} is on the y axis. Upregulated genes in SMS cortical neurons with p_{adj} less than 0.1 are indicated by red dots. Downregulated genes in SMS cortical neurons with p_{adj} less than 0.1 are indicated by blue dots. The gray line indicates $p_{adj} = 0.1$.

(C) GO analysis of SMS cortical neurons. The GO terms for downregulated genes (in blue) and upregulated genes (in red) and the respective $-\log_{10}(p_{adj})$ are shown.

(D) Violin plots of protocadherin gene expression from bulk RNA-seq in SMS and Ctrl cortical neurons. Each dot represents a sample. p_{adj} calculated by Wald test.

(E) Violin plots of expression of ion-exchanger genes from bulk RNA-seq in SMS and Ctrl cortical neurons. Each dot represents a sample. p_{adj} calculated by Wald test.

(F) Soma volume of SMS cortical neurons compared to Ctrl at 4 WPD. Left: representative 4-WPD soma 3D images of Ctrl and SMS cortical neurons transduced with myrGFP lentivirus. Scale bars, 50 μm. Right: barplot of soma volume of Ctrl and SMS neurons at 4 WPD ($n = 400$ cells per genotype, with 100 cells per cell line; each dot represents one soma and is colored based on cell line). $t = 3.412$, df $= 798$, $p = 0.0007$; unpaired t test.

(G) Representative 3D reconstituted neuron images of myrGFP-transduced Ctrl and SMS cortical neurons at 4 WPD. Scale bars, 50 μm.

(H) Quantification of total dendrite length in myrGFP$^+$ SMS and Ctrl cortical neurons at 4 WPD. $n = 40$ neurons per genotype with 10 neurons per cell line. Each dot represents one neuron. $U = 443$, $p = 0.0005$; two-tailed Mann-Whitney test.

(legend continued on next page)

II and III = single AP; types IV and V = repetitive AP; Figure S15C). We found similar proportions of Ctrl and SMS cortical neurons in immature (I and II/III) and mature (IV/V) categories (Figure S15D). We also observed similar proportions of type IV and type V mature Ctrl and SMS neurons (Figure S15E). These data confirmed that we could reproducibly generate high-purity cortical excitatory neurons and that del(17)p11.2 did not impact the proportions of cortical neurons in each AP category.

To investigate how del(17)p11.2 impacts the transcriptome of human cortical neurons, we performed bulk RNA-seq, which confirmed the downregulation of 40 del(17)p11.2 genes in SMS neurons (Figure 5A). DEG analysis identified a *trans* effect of del(17)p11.2 on gene expression, inducing 487 upregulated genes and 407 downregulated non-del(17)p11.2 genes in SMS cortical neurons (Figure 5B). GO analysis found that genes upregulated in SMS cortical neurons participate in small-molecule and ion binding and neuropeptide activity (i.e., *CCK* and *NTS*). In contrast, genes downregulated in SMS cortical neurons are involved in anatomical structure and multicellular organism development, including genes implicated in neurodevelopmental disorders (i.e., *CHD4*, *UBE3B*, *KDM5B*, and *SETD1B*) (Figure 5C). Selective protocadherin genes differentially expressed in SMS hiPSCs and NPCs, including *PCDHA5* and *PCDHGA6*, were similarly differentially expressed in SMS neurons (Figure 5D). Notably, SMS cortical neurons also showed differential expression of multiple genes encoding soluble ion exchangers. This includes the upregulation of *SLC24A4* and *SLC5A9* and downregulation of *SLC35E2B*, *SLC4A11*, and *SLC5A3* (Figure 5E), suggesting that neurons carrying del(17)p11.2 have abnormal cellular excitability.

The altered expression of neurodevelopmental and protocadherin genes led us to characterize how these changes affect SMS neuronal morphology. Ctrl and SMS neurons were transduced with lentivirus expressing synapsin promoter-driven myristoylated GFP (myrGFP) to target the neuronal membrane. SMS neurons had a significantly greater mean soma volume at 4 WPD (but not at 8 WPD) when compared to Ctrl neurons (Figures 5F and S15F). At 4WPD, 3D dendritic reconstruction showed that the mean total dendrite length was significantly greater in SMS neurons than in Ctrl neurons (Figures 5G and 5H). Sholl analysis found that SMS neurons at 4 WPD showed a greater neurite complexity compared to Ctrl neurons between 10 and 40 μm from the soma center (Figure 5I). Quantification of the critical radius (the Sholl radius at which a neuron has the greatest number of intersections) and the maximum number of crossings (the greatest number intersections of a neuron) found that SMS neurons have no differences in mean critical radii at 4 WPD but have a significantly greater mean maximum number of crossings compared to Ctrl neurons (Figure 5J). Analyses at 8 WPD showed no significant differences in overall dendrite complexity, critical radius, or the maximum number of crossings, but there was a lower mean total dendrite length (Figures S15G–S15J). Thus, SMS neurons showed an initial acceleration of neurite outgrowth at 4 WPD that was largely normalized by 8 WPD. To investigate how del(17)p11.2 impacts excitatory synapse formation, we quantified the density of $VGLUT1^{+}PSD95^{+}$ and $SYNAPSIN\text{-}1^{+}PSD95^{+}$ puncta contacting the $MAP2^{+}$ dendrites at 6 WPD (Figures 5K and S15K). SMS neurons showed an increased excitatory synapse formation compared to age-matched Ctrl neurons (Figures 5K and S15K). To determine whether the increased excitatory synapse density in SMS neurons corresponded with altered excitatory synaptic drive, we measured mEPSCs in Ctrl and SMS neurons. Ctrl and SMS neurons showed similar baseline mEPSC amplitudes (Figures S16A and S16B). We then induced synaptic upscaling with prolonged TTX treatment (1 μM for 24 h), whereby both Ctrl and SMS neurons significantly increased mEPSC amplitude in response to a sustained decrease in neuronal firing rate (Figures S16C and S16D). These data collectively demonstrate that SMS cortical neurons had neuronal morphological deficits and impaired expression of genes involved in neural development, ion binding, and neuropeptide activity, but they retained normal excitatory synaptic transmission and homeostatic upscaling.

Increased intrinsic excitability in SMS hiPSC-derived cortical neurons

To determine whether del(17)p11.2 alters the intrinsic neuronal excitability of human cortical neurons, we conducted whole-cell patch-clamp recordings under both V_{clamp} and I_{clamp} modes (Figure 6A). A hallmark of human cortical neurons is repetitive AP firing. At a holding potential close to the resting membrane potential of human cortical neurons (−70 mV), we found a higher percentage of mature (types IV/V) SMS neurons able to fire

(I) Sholl analysis of Ctrl and SMS cortical neurons at 4 WPD. $n = 80$ neurons per genotype with 20 neurons per cell line. Presented as differences between means (line) ± SEM (shade). Radius 10: $t = 3.514$, df = 158, $p_{adj} = 0.01247$; radius 20: $t = 5.203$, df = 158, $p_{adj} = 0.000014$; radius 30: $t = 4.404$, df = 158, $p_{adj} = 0.000449$; radius 40: $t = 3.517$, df = 158, $p_{adj} = 0.012467$; multiple t tests.
(J) Quantification of Sholl critical radius (left) and maximum crossings (right) of Ctrl and SMS cortical neurons at 4 WPD. $n = 80$ neurons per genotype with 20 neurons per cell line. Each dot represents one neuron. Critical radius: $U = 2{,}743$, $p = 0.1022$; maximum crossing: $U = 1{,}945$, $p < 0.0001$; two-tailed Mann-Whitney test.
(K) Quantification of excitatory synapse density ($VGLUT1^{+}$ and $PSD95^{+}$) in Ctrl and SMS cortical neurons at 6 WPD. Left: representative images of Ctrl and SMS neurons stained with MAP2 (gray), VGLUT1 (magenta), and PSD95 (cyan). Scale bars, 10 μm. Right: quantification of the number of puncta ($VGLUT1^{+}$ and $PSD95^{+}$) per μm. Each dot represents one 50-μm segment, $n = 40$ segments per genotype with 10 segments per cell line. $U = 365.5$, $p < 0.0001$; two-tailed Mann-Whitney test.

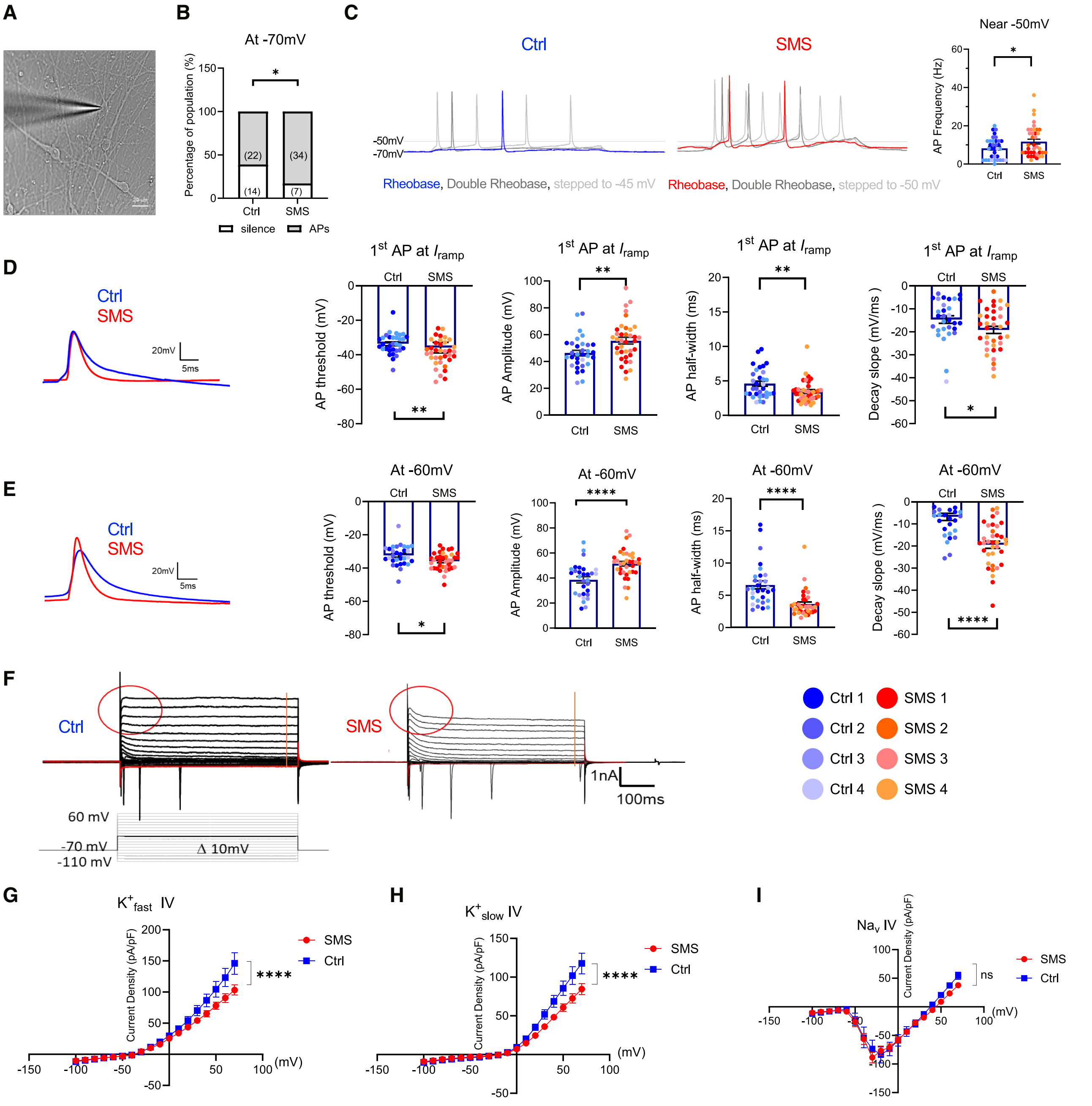

Figure 6. SMS hiPSC-derived cortical neurons showed altered AP properties and reduced potassium conductance that favored hyperexcitability

(A) Representative image of whole-cell patched hiPSC-derived cortical neuron. Scale bar, 20 μm.

(B) Percentage of neurons firing spontaneous APs in SMS versus Ctrl neurons (recorded at I_{clamp}, gap-free mode). Chi-squared = 4.599, df = 1, $p = 0.032$; chi-squared analysis. Cell numbers for each AP firing category are in parentheses.

(C) Evoked APs were elicited from a holding potential of −70 mV followed by 10-pA increment steps to the voltage close to −50 mV. The representative traces of AP number at rheobase, double rheobase (gray), and the voltage close to −50 mV (light gray) are presented. Right: AP frequency of Ctrl and SMS cortical neurons. $t = 2.188$, df = 66.03, $p = 0.0322$; Welch's t test.

(D) AP properties analyzed from the first AP evoked by a ramp test: 1-min ramp (nA/ms) followed by a hyperpolarized step (∼−90 mV). From left to right, the representative evoked AP of Ctrl (blue) and SMS (red) cortical neurons. AP threshold: $U = 367$, $p = 0.0063$; AP amplitude: $U = 361$, $p = 0.005$; AP half-width: $U = 392$, $p = 0.0156$; decay slope: $U = 360$, $p = 0.0297$; two-tailed Mann-Whitney tests.

(E) AP property analyses at a potential of −60 mV. From left to right, the representative AP of Ctrl (blue) and SMS (red) cortical neurons. AP threshold: $U = 347.5$, $p = 0.0126$; AP amplitude: $U = 227$, $p < 0.0001$; AP half-width: $U = 174$, $p < 0.0001$; decay slope: $U = 174$, $p < 0.0001$; two-tailed Mann-Whitney tests.

(F) Representative traces of whole-cell voltage clamp of Ctrl (left) and SMS (right) cortical neurons. The red circles and lines indicate the fast and slow voltage-gated components, respectively.

(legend continued on next page)

spontaneous APs than Ctrl neurons (Figure 6B), suggesting that SMS neurons were more active. The Ctrl and SMS neurons that did show spontaneous APs had similar firing frequencies (Figure S17A). To evoke APs and measure their properties, a current was injected to hold the resting membrane potential at −70 mV, followed by a step protocol (500 ms, 10 pA increment) ranging from a hyperpolarized potential to the potential at which neurons fail to fire APs. We found that at 9–11 WPD, categories IV/V SMS neurons could initiate APs at similar rheobases as Ctrl neurons (Figure S17B) but showed an increase in AP firing frequency at a potential close to −50 mV (Figure 6C). We further characterized AP properties from the first AP evoked under the I_{clamp} protocol, which more closely resembles changes in physiological membrane potentials (Figure 6D). Given that AP properties alter at different membrane potentials, we specifically analyzed AP properties at a potential of −60 mV, closer to the resting membrane potential of human cortical neurons (Figure 6E). Under both current-clamp protocols, SMS cortical neurons began to fire AP at a lower threshold and displayed larger AP overshoots, had a narrower AP half-width, and showed faster AP decays (Figures 6D and 6E). By contrast, membrane properties were similar between Ctrl and SMS cortical neurons (Figure S17C). These findings suggest that SMS cortical neurons could have altered ion-channel functions or compositions that drive changes in excitability. To examine whether excitatory neurons in 3D SMS organoids exhibit dysregulation of genes encoding synaptic proteins that could enhance neuronal excitability, we examined the expression of synaptic genes[80] in SMS Glu-N1 and Glu-N2 neurons. We found that *LMO7*, which encodes a cell-adhesion molecule with a PDZ domain, was overexpressed in both glutamatergic neuronal subtypes (Figure S17D). By contrast, most downregulated synaptic genes in SMS excitatory neurons belong to del(17)p11.2. Interestingly, LMO7 interacts with AFADIN and ACTININ, which are known to promote excitatory synapse function.[81,82] Therefore, *LMO7* overexpression might enhance neuronal excitability in Glu-N1 and Glu-N2 excitatory neurons.

To identify the origin of the changes in excitability, we analyzed the current density of voltage-gated potassium components (K^+_{fast} and K^+_{slow}) and the sodium component (Na_v) under the V_{clamp} mode. We found downshifted K^+_{fast} and K^+_{slow} *I-V* curves in SMS cortical neurons compared to Ctrl cortical neurons (Figures 6F–6H). By contrast, the Na_v component was intact (Figure 6I). This indicates that the greater AP overshoot of SMS cortical neurons was associated with reduced K^+ currents. Altogether, these data show that SMS neurons have altered AP firing and potassium channel function, which could contribute to cortical hyperexcitability observed in individuals with SMS.

Discussion

In this study, we demonstrated that hiPSC-derived neuronal and organoid models of SMS recapitulate features of human disease. Using these models, we identified how del(17)p11.2 alters neuronal development and function features that could potentially drive SMS pathology. While del(17)p11.2 is the known driver of the majority of SMS cases, the underlying molecular and neurobiological defects in SMS human brains have remained unclear due to a lack of relevant neural models. By differentiating SMS hiPSCs carrying del(17)p11.2 into 2D NPCs, 2D cortical neurons, and 3D cortical organoids, we were able to show how the deletion affects neuronal function and development.

We defined several key effects of del(17)p11.2 in human cellular models. First, del(17)p11.2 impacts gene expression in *cis* (for genes within the SMS locus) and in *trans* at multiple stages of cortical development. Second, del(17)p11.2 drives multi-scale chromatin rewiring in neural tissues and, to a lesser extent, in stem cells. The alteration of topological domains in SMS tissues could partially explain the *trans* effects of del(17)p11.2 on gene expression in hiPSCs. Third, SMS cortical organoids embody dysregulation of genes involved in cell-cycle regulation, metabolic processes, and neuronal signaling. Fourth, SMS cortical organoids showed impaired cell-cycle progression, reduced growth, and increased ventricle size. Fifth, SMS cortical neurons exhibited increased excitatory synapse density and an initially accelerated morphological complexity. Finally, SMS cortical neurons were more intrinsically excitable, which could be explained by reduced potassium conductance. Together, our data provide molecular and cellular mechanisms that explain how del(17)p11.2 could impact cortical development and function in SMS (summarized in Figure S18).

Individuals with SMS who carry del(17)p11.2 have a lower level of intellectual functioning as well as speech and motor delay and sensory issues not found in individuals with smaller deletions or *RAI1* mutations.[5] Therefore, it is likely that del(17)p11.2 impacts local chromatin organization and the global network of *trans* contacts that could contribute to these clinical phenotypes. Here, we performed chromosomal conformation capture analysis using SMS-relevant human-derived cell types and found that del(17)p11.2 induces significant alteration of

(G and H) *I-V* curves show that SMS cortical neurons exhibited decreased voltage-gated potassium fast component (G) and slow component (H) current density. K_{fast}: genotype = $F(1,1151) = 26.25$, $p < 0.0001$; K_{slow}: genotype = $F(1,1278) = 29.55$, $p < 0.001$; two-way ANOVA with Sidak's multiple comparison tests.

(I) *I-V* curve shows that the current density of voltage-gated sodium components was similar in SMS and Ctrl cortical neurons. Na_v: genotype = $F(1,1224) = 2.775$, $p = 0.096$; two-way ANOVA.

multi-scale chromosomal configurations. Locally, SMS hiPSCs and cortical organoids showed decreased *cis* contacts along chromosome 17 and increased chromosomal contacts flanking the SMS-deleted regions and within the deletion boundaries. In Ctrl hiPSCs and organoids, genes located within the SMS locus belonged to multiple TADs. In SMS tissues, the entire del(17)p11.2 region was encompassed in a newly formed TAD regardless of the size of the deletion, which could reshape the regulatory landscape of the remaining SMS genes in the intact chromosome. Therefore, del(17)p11.2 in one of the two homologous chromosomes was sufficient to induce increased contacts between the regions flanking del(17) p11.2, disrupt existing TAD boundaries, and induce the fusion of TADs. The precise mechanism of TAD fusion awaits further investigation.

Beyond chromosome 17, SMS cells showed altered inter-chromosomal contacts, with SMS cortical organoids showing significantly more differential chromosomal contacts than SMS hiPSCs. Importantly, we found evidence that differential TADs are associated with dysregulation of 70%–77% of non-del(17)p11.2 genes in SMS tissues. Together, these findings provide a plausible explanation by which an altered chromatin environment in SMS cells could cause global gene-expression defects in SMS. The relationship between chromatin conformation and steady-state gene expression is not always linear and deterministic.[83,84] Mounting evidence suggests that 3D chromosomal conformation changes could underlie neuronal activity-dependent transcriptional regulation or even beyond altering gene expression, such as neuronal responsiveness and circuit connectivity.[85–87] Therefore, del(17)p11.2-dependent TAD changes could contribute to neural dysfunction by disrupting the regulatory landscape critical for normal brain development. Understanding the precise mechanisms involved will require further investigation.

Our snRNA-seq experiments unveiled cell types and molecular pathways that could be associated with the clinical features of SMS. Specifically, del(17)p11.2 induces the most DEGs in tRG, migrating neurons, and glutamatergic neurons. The ventricle-contacting tRG are important for forming the cortical ventricles, and they provide an architectural basis for brain expansion.[88] Our pathway enrichment analysis revealed candidate pathways potentially associated with cortical dysfunction in SMS. Specifically, multiple cell clusters showed downregulation of genes in metabolic and biosynthetic pathways and upregulation of genes involved in DNA transcription and cell-cycle regulation. These changes suggest that del(17) p11.2 contributes to metabolic dysfunction and cortical abnormalities in individuals with SMS. Additionally, we found that SMS organoid gene signatures mirrored those of ASD and SCZ signatures. In particular, genes upregulated in astroglia, tRG, migrating neurons, and glutamatergic neurons were enriched for ASD and SCZ signatures. This, together with the larger number of DEGs found in glutamatergic neurons when compared to GABAergic neurons, highlights that del(17)p11.2 preferentially impacts transcriptional programs in excitatory neurons, versus other neuronal types, to affect clinical phenotypes. By further differentiating SMS NPCs into a relatively homogenous population of 2D cortical excitatory neurons, we found that SMS cortical neurons showed overexpression of SCZ-associated *PCDHA3* and *PCDHA5*,[89,90] neurodevelopmental disorder-associated *PCDHGA5*,[91] and underexpression of *PCDH15*, which is associated with Usher syndrome type I (MIM: 602083)[92] and bipolar affective disorder.[93] Members of the *PCDH* family often localize to neuronal surfaces to facilitate dendritic arborization,[94] which could contribute to the increased dendritic complexity we found in 4-WPD SMS cortical neurons. These data indicate that a salient feature of SMS neural tissues is the altered expression of protocadherins, which could contribute to aberrant synapse assembly and neuronal connectivity.[89,94,95] These findings are consistent with our previous RNA-seq findings in *Rai1* mutant mice[70] and together indicate that del(17)p11.2 and RAI1 are important for regulating the expression of cell-surface molecules.

Neuroanatomical hallmarks in SMS include mild ventriculomegaly[8] and reduced gray matter volume.[7] Interestingly, prenatal brain imaging found mild lateral ventriculomegaly in fetuses carrying chromosomal deletions spanning the SMS region,[11] consistent with an embryonic origin of the phenotype. Ventriculomegaly is found across neuropsychiatric diseases, including autism and SCZ, and is associated with dysregulation of prenatal NPCs.[13,96] For example, loss of *SOX2* or *FOXG1*, genes critical for NPC proliferation, results in enlarged lateral ventricles in mouse models and humans with SMS.[97,98] Reduced cortical organoid growth is also associated with NPC abnormalities. Similar to our findings in SMS cortical organoids, cortical organoids carrying *TCF4* mutations showed an increased proportion of cycling progenitors, reduced proliferative capacity, and reduced organoid size.[27] Disrupted cell-cycle regulation is also observed in several mouse and human neuronal models carrying mutations of ASD-risk genes such as 16p11.2[99] and *MECP2*.[100] In line with these studies, we found that SMS organoids showed reduced size and ventriculomegaly as well as early cell-cycle exits and misregulation of cell-cycle pathways in oRG and the ventricle-forming tRG.[88] This was further supported by our 2D SMS NPC model, which showed impaired cell-cycle progression (G_1 stalling) and increased genomic instability. Together, these data suggest that del (17)p11.2 induces misexpression of cell-cycle genes, reduced progenitor proliferative capacity, defective ventricle formation, and impaired cortical organoid growth. In contrast to our findings in human SMS cortical organoids, neuroanatomical studies in the SMS mouse model did not find SMS-like cortex volume reduction or ventriculomegaly.[17] This is likely due to human cortical organoids containing molecular pathways and cell types

that do not exist in mouse models. For example, our snRNA-seq data found profound gene-expression changes in ventricle-contacting tRG, a type of NPC only found in gyrencephalic mammals and not in lissencephalic species like the mouse.[88,101] Moreover, multiple cell clusters in SMS organoids shared downregulation of primate-specific genes *POTEI* and *POTEF*.[102] The loss of *POTEI* is implicated in hearing loss,[103] which warrants further investigation given that individuals with SMS commonly suffer from hearing loss.[104] Therefore, while mouse models continue to be useful, given that they recapitulate cortical hyperexcitability and obesity in SMS,[14,15,17] the early neurodevelopmental phenotypes of SMS are better recapitulated using human hiPSC-derived neural models.

SMS is frequently associated with cortical epileptiform electroencephalographic abnormalities.[105] Here, we found that SMS cortical neurons displayed hyperexcitability and increased AP firing due to a lower AP firing threshold, higher overshoot, and sharper waveforms. The narrower AP waveform indicates that SMS neuronal membranes repolarize faster, potentiating the Na_v channel opening and facilitating the next AP firing. These property changes likely enable higher-frequency AP firing in SMS cortical neurons. In our previous study, we found neuronal hyperexcitability in *Rai1*-deficient dentate gyrus granule cells, which was associated with a lower AP threshold and narrower AP waveforms.[17] These changes were attributable to altered expression of a T-type calcium channel $Ca_v3.1$ and potassium channels *HCN1* and *HCN4*. By contrast, our bulk RNA-seq data found differential expression of the solute carrier (SLC) family in SMS cortical neurons. These SLC transporters determine the exchange of ions, nutrients, metabolites, and drugs across membranes.[106,107] SLCs can modulate neuronal excitability directly via their channel-like properties to allow for ion exchange and modulation of the function of other synaptic channels, or indirectly through controlling the substrate transmission at synapses. Our RNA-seq data showed increased expression of *SLC24A4* and *SLC5A9* and decreased expression of several SLC members, including *SLC5A3* (STIM1) and *SLC38A4*. Members of the SLC38A subfamily are involved in glutamate transport and affect synaptic transmission.[106] STIM1 is a myo-inositol transporter, which enhances potassium outflux via KCNQ2/3 channels.[108] SMS cortical neurons showed a decreased *STIM1* expression, which could contribute to reduced K^+ current density. In our experiments, we detected decreased potassium current density and a robust AP waveform change. Our RNA-seq data in 2D SMS neurons also revealed an upregulation of the potassium channel regulatory subunit *KCNAB3* (encoding Kvβ3) in SMS neurons (Figure S17E). Upregulation of Kvβ3 could lead to reduced K^+ currents and delayed repolarization, thus favoring frequent firing and neuronal hyperexcitability.[109] The direct involvement of potassium-channel dysfunction in SMS neuronal hyperexcitability requires further pharmacological investigation. Studying the role of *KCNAB3* in SMS-associated channelopathy, such as blocking Kv1 channels and interrupting Kvβ3- Kvα interaction in SMS neurons, can be a future direction. Because the 2D differentiation protocol used in our study favored the generation of excitatory neurons, whether del(17)p11.2 affects the intrinsic properties of GABAergic neurons also remains to be investigated. Furthermore, future efforts studying the cellular composition of SMS organoids at later time points could help decipher the contribution of different cell types to neuronal hyperexcitability.

While our study exclusively focused on modeling the 90% of SMS cases that carry del(17)p11.2, future studies that model the remaining 10% of SMS cases with *RAI1* heterozygous mutations will enable a full understanding of disease pathology. This study also focused on hiPSCs derived from women due to the higher prevalence of ASD symptoms in women with SMS compared to men. Further studies in men SMS hiPSC-derived neuronal models would complement these findings. Moreover, future multi-electrode arrays experiments using SMS organoids will help determine whether they also show deficits in excitability. Finally, we did not directly compare transcriptomic defects of hiPSC-derived 2D cortical neurons and 3D cortical organoids due to differences in maturation status, growth environment, culturing conditions (attached versus floating), the diversity of cell types, and sequencing methods. An RNA-seq study performed in hiPSC-derived cerebral organoids and 2D cortical neurons showed transcriptomic differences between these two models.[110] Despite these limitations, our work represents an important step toward using SMS-derived neural models to delineate SMS pathophysiology in a disease-relevant *in vitro* system. This study thus provides a framework for future studies to dissect molecular mechanisms underlying disease phenotypes and to develop therapeutics.

Data and code availability

The FASTQ files for Hi-C (GEO: GSE298284) and snRNA-seq (GEO: GSE295166) generated in this study were deposited in the NCBI Gene Expression Omnibus database. Bulk RNA-seq data are available at Zenodo (https://doi.org/10.5281/zenodo.15391244). Hi-C and snRNA-seq reads were aligned to the human genome reference GRCh38. The code used in the study is publicly available.

Acknowledgments

This work was funded by the Canadian Institutes of Health Research (CIHR), the SMS Research Foundation, Campus – Espace de formation, Dr. Anne-Sophie Villeneuve, and Dr. Simon Lafrenière. We thank the iPSC cell reprogramming core facility of CHU Sainte-Justine for assisting with hiPSC generation.

Author contributions

This work was conceptualized by Y.-J.L., Y.-T.C., X.G., and W.-H. H. Experiments, data analysis, and figures were done by Y.-J.L., Y.-T.C., Y.C., M.K., A.D., A.P., S.K., F.L., and Q.Z. Writing was

done by Y.-J.L. and Y.-T.C. and editing by W.-H.H., with input from all authors.

Declaration of interests

The authors declare no competing interests.

Supplemental information

Supplemental information can be found online at https://doi.org/10.1016/j.ajhg.2025.07.020.

Web resources

Human Brain Transcriptome atlas, https://hbatlas.org
OMIM, https://www.omim.org/
R package fgsea, http://bioconductor.org/packages/fgsea/
R package GeneOverlap, https://bioconductor.org/packages/release/bioc/html/GeneOverlap.html
Velocyto, https://bioconductor.org/packages/release/bioc/html/velociraptor.html

Received: February 24, 2025
Accepted: July 31, 2025
Published: August 28, 2025

References

1. Javed, S., Selliah, T., Lee, Y.J., and Huang, W.H. (2020). Dosage-sensitive genes in autism spectrum disorders: From neurobiology to therapy. Neurosci. Biobehav. Rev. *118*, 538–567. https://doi.org/10.1016/j.neubiorev.2020.08.009.
2. Laje, G., Morse, R., Richter, W., Ball, J., Pao, M., and Smith, A.C.M. (2010). Autism spectrum features in Smith-Magenis syndrome. Am. J. Med. Genet. C Semin. Med. Genet. *154C*, 456–462. https://doi.org/10.1002/ajmg.c.30275.
3. Nag, H.E., Nordgren, A., Anderlid, B.M., and Nærland, T. (2018). Reversed gender ratio of autism spectrum disorder in Smith-Magenis syndrome. Mol. Autism *9*, 1. https://doi.org/10.1186/s13229-017-0184-2.
4. Slager, R.E., Newton, T.L., Vlangos, C.N., Finucane, B., and Elsea, S.H. (2003). Mutations in RAI1 associated with Smith-Magenis syndrome. Nat. Genet. *33*, 466–468. https://doi.org/10.1038/ng1126.
5. Girirajan, S., Vlangos, C.N., Szomju, B.B., Edelman, E., Trevors, C.D., Dupuis, L., Nezarati, M., Bunyan, D.J., and Elsea, S.H. (2006). Genotype-phenotype correlation in Smith-Magenis syndrome: evidence that multiple genes in 17p11.2 contribute to the clinical spectrum. Genet. Med. *8*, 417–427. https://doi.org/10.1097/01.gim.0000228215.32110.89.
6. Linders, C.C., van Eeghen, A.M., Zinkstok, J.R., van den Boogaard, M.J., and Boot, E. (2023). Intellectual and Behavioral Phenotypes of Smith-Magenis Syndrome: Comparisons between Individuals with a 17p11.2 Deletion and Pathogenic RAI1 Variant. Genes *14*, 1514. https://doi.org/10.3390/genes14081514.
7. Boddaert, N., De Leersnyder, H., Bourgeois, M., Munnich, A., Brunelle, F., and Zilbovicius, M. (2004). Anatomical and functional brain imaging evidence of lenticulo-insular anomalies in Smith Magenis syndrome. Neuroimage *21*, 1021–1025. https://doi.org/10.1016/j.neuroimage.2003.10.040.
8. Greenberg, F., Lewis, R.A., Potocki, L., Glaze, D., Parke, J., Killian, J., Murphy, M.A., Williamson, D., Brown, F., Dutton, R., et al. (1996). Multi-disciplinary clinical study of Smith-Magenis syndrome (deletion 17p11.2). Am. J. Med. Genet. *62*, 247–254. https://doi.org/10.1002/(SICI)1096-8628(19960329)62:3<247::AID-AJMG9>3.0.CO;2-Q.
9. Nijim, Y., Adawi, A., Bisharat, B., and Bowirrat, A. (2016). First Case Report of Smith-Magenis Syndrome (SMS) Among the Arab Community in Nazareth: View and Overview. Medicine (Baltim.) *95*, e2362. https://doi.org/10.1097/MD.0000000000002362.
10. Natacci, F., Corrado, L., Pierri, M., Rossetti, M., Zuccarini, C., Riva, P., Miozzo, M., and Larizza, L. (2000). Patient with large 17p11.2 deletion presenting with Smith-Magenis syndrome and Joubert syndrome phenotype. Am. J. Med. Genet. *95*, 467–472. https://doi.org/10.1002/1096-8628(20001218)95:5<467::aid-ajmg11>3.0.co;2-t.
11. Lei, T.Y., Li, R., Fu, F., Wan, J.H., Zhang, Y.L., Jing, X.Y., and Liao, C. (2016). Prenatal diagnosis of Smith-Magenis syndrome in two fetuses with increased nuchal translucency, mild lateral ventriculomegaly, and congenital heart defects. Taiwan. J. Obstet. Gynecol. *55*, 886–890. https://doi.org/10.1016/j.tjog.2015.07.007.
12. Zhang, P., Sun, Y., Tian, H., Rong, L., Wang, F., Yu, X., Li, Y., and Gao, J. (2020). Prenatal diagnosis and neonatal phenotype of a de novo microdeletion of 17p11.2p12 associated with Smith-Magenis syndrome and external genital defects. J. Genet. *99*, 50.
13. Duy, P.Q., Rakic, P., Alper, S.L., Butler, W.E., Walsh, C.A., Sestan, N., Geschwind, D.H., Jin, S.C., and Kahle, K.T. (2022). Brain ventricles as windows into brain development and disease. Neuron *110*, 12–15. https://doi.org/10.1016/j.neuron.2021.12.009.
14. Javed, S., Chang, Y.T., Cho, Y., Lee, Y.J., Chang, H.C., Haque, M., Lin, Y.C., and Huang, W.H. (2023). Smith-Magenis syndrome protein RAI1 regulates body weight homeostasis through hypothalamic BDNF-producing neurons and neurotrophin downstream signalling. eLife *12*, 90333. https://doi.org/10.7554/eLife.90333.
15. Javed, S., Lee, Y.J., Xu, J., and Huang, W.H. (2021). Temporal dissection of Rai1 function reveals brain-derived neurotrophic factor as a potential therapeutic target for Smith-Magenis syndrome. Hum. Mol. Genet. *31*, 275–288. https://doi.org/10.1093/hmg/ddab245.
16. Huang, W.H., Wang, D.C., Allen, W.E., Klope, M., Hu, H., Shamloo, M., and Luo, L. (2018). Early adolescent Rai1 reactivation reverses transcriptional and social interaction deficits in a mouse model of Smith-Magenis syndrome. Proc. Natl. Acad. Sci. USA *115*, 10744–10749. https://doi.org/10.1073/pnas.1806796115.
17. Chang, Y.T., Kowalczyk, M., Fogerson, P.M., Lee, Y.J., Haque, M., Adams, E.L., Wang, D.C., DeNardo, L.A., Tessier-Lavigne, M., Huguenard, J.R., et al. (2022). Loss of Rai1 enhances hippocampal excitability and epileptogenesis in mouse models of Smith-Magenis syndrome. Proc. Natl. Acad. Sci. USA *119*, e2210122119. https://doi.org/10.1073/pnas.2210122119.
18. Mora-Bermudez, F., Badsha, F., Kanton, S., Camp, J.G., Vernot, B., Kohler, K., Voigt, B., Okita, K., Maricic, T., He, Z., et al. (2016). Differences and similarities between human and chimpanzee neural progenitors during cerebral cortex

development. eLife *5*, 18683. https://doi.org/10.7554/eLife.18683.

19. Otani, T., Marchetto, M.C., Gage, F.H., Simons, B.D., and Livesey, F.J. (2016). 2D and 3D Stem Cell Models of Primate Cortical Development Identify Species-Specific Differences in Progenitor Behavior Contributing to Brain Size. Cell Stem Cell *18*, 467–480. https://doi.org/10.1016/j.stem.2016.03.003.
20. Hodge, R.D., Bakken, T.E., Miller, J.A., Smith, K.A., Barkan, E.R., Graybuck, L.T., Close, J.L., Long, B., Johansen, N., Penn, O., et al. (2019). Conserved cell types with divergent features in human versus mouse cortex. Nature *573*, 61–68. https://doi.org/10.1038/s41586-019-1506-7.
21. Zemke, N.R., Armand, E.J., Wang, W., Lee, S., Zhou, J., Li, Y. E., Liu, H., Tian, W., Nery, J.R., Castanon, R.G., et al. (2023). Conserved and divergent gene regulatory programs of the mammalian neocortex. Nature *624*, 390–402. https://doi.org/10.1038/s41586-023-06819-6.
22. Kalmbach, B.E., Buchin, A., Long, B., Close, J., Nandi, A., Miller, J.A., Bakken, T.E., Hodge, R.D., Chong, P., de Frates, R., et al. (2018). h-Channels Contribute to Divergent Intrinsic Membrane Properties of Supragranular Pyramidal Neurons in Human versus Mouse Cerebral Cortex. Neuron *100*, 1194–1208.e5. https://doi.org/10.1016/j.neuron.2018.10.012.
23. Eyal, G., Verhoog, M.B., Testa-Silva, G., Deitcher, Y., Lodder, J.C., Benavides-Piccione, R., Morales, J., DeFelipe, J., de Kock, C.P., Mansvelder, H.D., and Segev, I. (2016). Unique membrane properties and enhanced signal processing in human neocortical neurons. eLife *5*, e16553. https://doi.org/10.7554/eLife.16553.
24. Ghosh, R.P., and Meyer, B.J. (2021). Spatial Organization of Chromatin: Emergence of Chromatin Structure During Development. Annu. Rev. Cell Dev. Biol. *37*, 199–232. https://doi.org/10.1146/annurev-cellbio-032321-035734.
25. Wallace, J.L., and Pollen, A.A. (2024). Human neuronal maturation comes of age: cellular mechanisms and species differences. Nat. Rev. Neurosci. *25*, 7–29. https://doi.org/10.1038/s41583-023-00760-3.
26. Fujita, Y., Pather, S.R., Ming, G.L., and Song, H. (2022). 3D spatial genome organization in the nervous system: From development and plasticity to disease. Neuron *110*, 2902–2915. https://doi.org/10.1016/j.neuron.2022.06.004.
27. Papes, F., Camargo, A.P., de Souza, J.S., Carvalho, V.M.A., Szeto, R.A., LaMontagne, E., Teixeira, J.R., Avansini, S.H., Sánchez-Sánchez, S.M., Nakahara, T.S., et al. (2022). Transcription Factor 4 loss-of-function is associated with deficits in progenitor proliferation and cortical neuron content. Nat. Commun. *13*, 2387. https://doi.org/10.1038/s41467-022-29942-w.
28. Rylaarsdam, L., Rakotomamonjy, J., Pope, E., and Guemez-Gamboa, A. (2024). iPSC-derived models of PACS1 syndrome reveal transcriptional and functional deficits in neuron activity. Nat. Commun. *15*, 827. https://doi.org/10.1038/s41467-024-44989-7.
29. Blair, J.D., Hockemeyer, D., and Bateup, H.S. (2018). Genetically engineered human cortical spheroid models of tuberous sclerosis. Nat. Med. *24*, 1568–1578. https://doi.org/10.1038/s41591-018-0139-y.
30. Khan, T.A., Revah, O., Gordon, A., Yoon, S.J., Krawisz, A.K., Goold, C., Sun, Y., Kim, C.H., Tian, Y., Li, M.Y., et al. (2020). Neuronal defects in a human cellular model of 22q11.2 deletion syndrome. Nat. Med. *26*, 1888–1898. https://doi.org/10.1038/s41591-020-1043-9.
31. Sundberg, M., Pinson, H., Smith, R.S., Winden, K.D., Venugopal, P., Tai, D.J.C., Gusella, J.F., Talkowski, M.E., Walsh, C.A., Tegmark, M., and Sahin, M. (2021). 16p11.2 deletion is associated with hyperactivation of human iPSC-derived dopaminergic neuron networks and is rescued by RHOA inhibition in vitro. Nat. Commun. *12*, 2897. https://doi.org/10.1038/s41467-021-23113-z.
32. Sebastian, R., Jin, K., Pavon, N., Bansal, R., Potter, A., Song, Y., Babu, J., Gabriel, R., Sun, Y., Aronow, B., and Pak, C. (2023). Schizophrenia-associated NRXN1 deletions induce developmental-timing- and cell-type-specific vulnerabilities in human brain organoids. Nat. Commun. *14*, 3770. https://doi.org/10.1038/s41467-023-39420-6.
33. Chambers, S.M., Fasano, C.A., Papapetrou, E.P., Tomishima, M., Sadelain, M., and Studer, L. (2009). Highly efficient neural conversion of human ES and iPS cells by dual inhibition of SMAD signaling. Nat. Biotechnol. *27*, 275–280. https://doi.org/10.1038/nbt.1529.
34. Mariani, J., Simonini, M.V., Palejev, D., Tomasini, L., Coppola, G., Szekely, A.M., Horvath, T.L., and Vaccarino, F.M. (2012). Modeling human cortical development in vitro using induced pluripotent stem cells. Proc. Natl. Acad. Sci. USA *109*, 12770–12775. https://doi.org/10.1073/pnas.1202944109.
35. Shi, Y., Kirwan, P., Smith, J., Robinson, H.P.C., and Livesey, F. J. (2012). Human cerebral cortex development from pluripotent stem cells to functional excitatory synapses. Nat. Neurosci. *15*, 477–S1. https://doi.org/10.1038/nn.3041.
36. Yoon, S.J., Elahi, L.S., Paşca, A.M., Marton, R.M., Gordon, A., Revah, O., Miura, Y., Walczak, E.M., Holdgate, G.M., Fan, H.C., et al. (2019). Reliability of human cortical organoid generation. Nat. Methods *16*, 75–78. https://doi.org/10.1038/s41592-018-0255-0.
37. Watson, J.V., Chambers, S.H., and Smith, P.J. (1987). A pragmatic approach to the analysis of DNA histograms with a definable G1 peak. Cytometry *8*, 1–8. https://doi.org/10.1002/cyto.990080101.
38. Ewels, P.A., Peltzer, A., Fillinger, S., Patel, H., Alneberg, J., Wilm, A., Garcia, M.U., Di Tommaso, P., and Nahnsen, S. (2020). The nf-core framework for community-curated bioinformatics pipelines. Nat. Biotechnol. *38*, 276–278. https://doi.org/10.1038/s41587-020-0439-x.
39. Servant, N., Varoquaux, N., Lajoie, B.R., Viara, E., Chen, C. J., Vert, J.P., Heard, E., Dekker, J., and Barillot, E. (2015). HiC-Pro: an optimized and flexible pipeline for Hi-C data processing. Genome Biol. *16*, 259. https://doi.org/10.1186/s13059-015-0831-x.
40. Abdennur, N., and Mirny, L.A. (2020). Cooler: scalable storage for Hi-C data and other genomically labeled arrays. Bioinformatics *36*, 311–316. https://doi.org/10.1093/bioinformatics/btz540.
41. Ramirez, F., Bhardwaj, V., Arrigoni, L., Lam, K.C., Gruning, B.A., Villaveces, J., Habermann, B., Akhtar, A., and Manke, T. (2018). High-resolution TADs reveal DNA sequences underlying genome organization in flies. Nat. Commun. *9*, 189. https://doi.org/10.1038/s41467-017-02525-w.
42. Lun, A.T.L., and Smyth, G.K. (2015). diffHic: a Bioconductor package to detect differential genomic interactions in Hi-C data. BMC Bioinf. *16*, 258. https://doi.org/10.1186/s12859-015-0683-0.

43. Robinson, M.D., McCarthy, D.J., and Smyth, G.K. (2010). edgeR: a Bioconductor package for differential expression analysis of digital gene expression data. Bioinformatics *26*, 139–140. https://doi.org/10.1093/bioinformatics/btp616.
44. Zheng, G.X.Y., Terry, J.M., Belgrader, P., Ryvkin, P., Bent, Z. W., Wilson, R., Ziraldo, S.B., Wheeler, T.D., McDermott, G. P., Zhu, J., et al. (2017). Massively parallel digital transcriptional profiling of single cells. Nat. Commun. *8*, 14049. https://doi.org/10.1038/ncomms14049.
45. Hao, Y., Hao, S., Andersen-Nissen, E., Mauck, W.M., 3rd, Zheng, S., Butler, A., Lee, M.J., Wilk, A.J., Darby, C., Zager, M., et al. (2021). Integrated analysis of multimodal single-cell data. Cell *184*, 3573–3587.e29. https://doi.org/10.1016/j.cell.2021.04.048.
46. Germain, P.L., Lun, A., Garcia Meixide, C., Macnair, W., and Robinson, M.D. (2021). Doublet identification in single-cell sequencing data using scDblFinder. F1000Res. *10*, 979. https://doi.org/10.12688/f1000research.73600.2.
47. Mangiola, S., Roth-Schulze, A.J., Trussart, M., Zozaya-Valdés, E., Ma, M., Gao, Z., Rubin, A.F., Speed, T.P., Shim, H., and Papenfuss, A.T. (2023). sccomp: Robust differential composition and variability analysis for single-cell data. Proc. Natl. Acad. Sci. USA *120*, e2203828120. https://doi.org/10.1073/pnas.2203828120.
48. Robinson, M.D., and Oshlack, A. (2010). A scaling normalization method for differential expression analysis of RNA-seq data. Genome Biol. *11*, R25. https://doi.org/10.1186/gb-2010-11-3-r25.
49. Ritchie, M.E., Phipson, B., Wu, D., Hu, Y., Law, C.W., Shi, W., and Smyth, G.K. (2015). limma powers differential expression analyses for RNA-sequencing and microarray studies. Nucleic Acids Res. *43*, e47. https://doi.org/10.1093/nar/gkv007.
50. Kuleshov, M.V., Jones, M.R., Rouillard, A.D., Fernandez, N. F., Duan, Q., Wang, Z., Koplev, S., Jenkins, S.L., Jagodnik, K. M., Lachmann, A., et al. (2016). Enrichr: a comprehensive gene set enrichment analysis web server 2016 update. Nucleic Acids Res. *44*, W90–W97. https://doi.org/10.1093/nar/gkw377.
51. Sayols, S. (2023). rrvgo: a Bioconductor package for interpreting lists of Gene Ontology terms. MicroPubl. Biol. *2023*, 000811. https://doi.org/10.17912/micropub.biology.000811.
52. Wang, L., Mirabella, V.R., Dai, R., Su, X., Xu, R., Jadali, A., Bernabucci, M., Singh, I., Chen, Y., Tian, J., et al. (2024). Analyses of the autism-associated neuroligin-3 R451C mutation in human neurons reveal a gain-of-function synaptic mechanism. Mol. Psychiatry *29*, 1620–1635. https://doi.org/10.1038/s41380-022-01834-x.
53. Kang, H.J., Kawasawa, Y.I., Cheng, F., Zhu, Y., Xu, X., Li, M., Sousa, A.M.M., Pletikos, M., Meyer, K.A., Sedmak, G., et al. (2011). Spatio-temporal transcriptome of the human brain. Nature *478*, 483–489. https://doi.org/10.1038/nature10523.
54. Bergen, V., Lange, M., Peidli, S., Wolf, F.A., and Theis, F.J. (2020). Generalizing RNA velocity to transient cell states through dynamical modeling. Nat. Biotechnol. *38*, 1408–1414. https://doi.org/10.1038/s41587-020-0591-3.
55. La Manno, G., Soldatov, R., Zeisel, A., Braun, E., Hochgerner, H., Petukhov, V., Lidschreiber, K., Kastriti, M.E., Lönnerberg, P., Furlan, A., et al. (2018). RNA velocity of single cells. Nature *560*, 494–498. https://doi.org/10.1038/s41586-018-0414-6.
56. Raudvere, U., Kolberg, L., Kuzmin, I., Arak, T., Adler, P., Peterson, H., and Vilo, J. (2019). g:Profiler: a web server for functional enrichment analysis and conversions of gene lists (2019 update). Nucleic Acids Res. *47*, W191–W198. https://doi.org/10.1093/nar/gkz369.
57. Huang, W.H., Tupal, S., Huang, T.W., Ward, C.S., Neul, J.L., Klisch, T.J., Gray, P.A., and Zoghbi, H.Y. (2012). Atoh1 governs the migration of postmitotic neurons that shape respiratory effectiveness at birth and chemoresponsiveness in adulthood. Neuron *75*, 799–809. https://doi.org/10.1016/j.neuron.2012.06.027.
58. Chang, H.C., Lee, Y.J., Javed, S., Haque, M., Chang, Y.T., Lin, Y.C., Oram, C., and Huang, W.H. (2023). rAAV-CRISPRa therapy corrects Rai1 haploinsufficiency and rescues selective disease features in Smith-Magenis syndrome mice. J. Biol. Chem. *299*, 102728. https://doi.org/10.1016/j.jbc.2022.102728.
59. Chang, Y.T., Lee, Y.J., Haque, M., Chang, H.C., Javed, S., Lin, Y.C., Cho, Y., Abramovitz, J., Chin, G., Khamis, A., et al. (2024). Comparative analyses of the Smith-Magenis syndrome protein RAI1 in mice and common marmoset monkeys. J. Comp. Neurol. *532*, e25589. https://doi.org/10.1002/cne.25589.
60. Kowalczyk, M., Lee, Y.J., and Huang, W.H. (2025). TSC2-mTORC1 axis regulates morphogenesis and neurological function of Gli1(+) adult-born dentate granule cells. Mol. Biol. Cell *36*, br1. https://doi.org/10.1091/mbc.E24-08-0366.
61. Bardy, C., van den Hurk, M., Kakaradov, B., Erwin, J.A., Jaeger, B.N., Hernandez, R.V., Eames, T., Paucar, A.A., Gorris, M., Marchand, C., et al. (2016). Predicting the functional states of human iPSC-derived neurons with single-cell RNA-seq and electrophysiology. Mol. Psychiatry *21*, 1573–1588. https://doi.org/10.1038/mp.2016.158.
62. Zhang, X., Zhang, Y., Zhu, X., Purmann, C., Haney, M.S., Ward, T., Khechaduri, A., Yao, J., Weissman, S.M., and Urban, A.E. (2018). Local and global chromatin interactions are altered by large genomic deletions associated with human brain development. Nat. Commun. *9*, 5356. https://doi.org/10.1038/s41467-018-07766-x.
63. Melo, U.S., Schöpflin, R., Acuna-Hidalgo, R., Mensah, M.A., Fischer-Zirnsak, B., Holtgrewe, M., Klever, M.K., Türkmen, S., Heinrich, V., Pluym, I.D., et al. (2020). Hi-C Identifies Complex Genomic Rearrangements and TAD-Shuffling in Developmental Diseases. Am. J. Hum. Genet. *106*, 872–884. https://doi.org/10.1016/j.ajhg.2020.04.016.
64. Lieberman-Aiden, E., van Berkum, N.L., Williams, L., Imakaev, M., Ragoczy, T., Telling, A., Amit, I., Lajoie, B.R., Sabo, P.J., Dorschner, M.O., et al. (2009). Comprehensive mapping of long-range interactions reveals folding principles of the human genome. Science *326*, 289–293. https://doi.org/10.1126/science.1181369.
65. Dixon, J.R., Selvaraj, S., Yue, F., Kim, A., Li, Y., Shen, Y., Hu, M., Liu, J.S., and Ren, B. (2012). Topological domains in mammalian genomes identified by analysis of chromatin interactions. Nature *485*, 376–380. https://doi.org/10.1038/nature11082.
66. Lupianez, D.G., Kraft, K., Heinrich, V., Krawitz, P., Brancati, F., Klopocki, E., Horn, D., Kayserili, H., Opitz, J.M., Laxova, R., et al. (2015). Disruptions of topological chromatin domains cause pathogenic rewiring of gene-enhancer interactions. Cell *161*, 1012–1025. https://doi.org/10.1016/j.cell.2015.04.004.

67. Xiao, J.Y., Hafner, A., and Boettiger, A.N. (2021). How subtle changes in 3D structure can create large changes in transcription. eLife *10*, e64320. https://doi.org/10.7554/eLife.64320.
68. Lopez-Tobon, A., Shyti, R., Villa, C.E., Cheroni, C., Fuentes-Bravo, P., Trattaro, S., Caporale, N., Troglio, F., Tenderini, E., Mihailovich, M., et al. (2023). GTF2I dosage regulates neuronal differentiation and social behavior in 7q11.23 neurodevelopmental disorders. Sci. Adv. *9*, eadh2726. https://doi.org/10.1126/sciadv.adh2726.
69. Uzquiano, A., Kedaigle, A.J., Pigoni, M., Paulsen, B., Adiconis, X., Kim, K., Faits, T., Nagaraja, S., Antón-Bolaños, N., Gerhardinger, C., et al. (2022). Proper acquisition of cell class identity in organoids allows definition of fate specification programs of the human cerebral cortex. Cell *185*, 3770–3788.e27. https://doi.org/10.1016/j.cell.2022.09.010.
70. Huang, W.H., Guenthner, C.J., Xu, J., Nguyen, T., Schwarz, L.A., Wilkinson, A.W., Gozani, O., Chang, H.Y., Shamloo, M., and Luo, L. (2016). Molecular and Neural Functions of Rai1, the Causal Gene for Smith-Magenis Syndrome. Neuron *92*, 392–406. https://doi.org/10.1016/j.neuron.2016.09.019.
71. Renthal, W., Boxer, L.D., Hrvatin, S., Li, E., Silberfeld, A., Nagy, M.A., Griffith, E.C., Vierbuchen, T., and Greenberg, M.E. (2018). Characterization of human mosaic Rett syndrome brain tissue by single-nucleus RNA sequencing. Nat. Neurosci. *21*, 1670–1679. https://doi.org/10.1038/s41593-018-0270-6.
72. Kang, Y., Zhou, Y., Li, Y., Han, Y., Xu, J., Niu, W., Li, Z., Liu, S., Feng, H., Huang, W., et al. (2021). A human forebrain organoid model of fragile X syndrome exhibits altered neurogenesis and highlights new treatment strategies. Nat. Neurosci. *24*, 1377–1391. https://doi.org/10.1038/s41593-021-00913-6.
73. Sarieva, K., Kagermeier, T., Khakipoor, S., Atay, E., Yentür, Z., Becker, K., and Mayer, S. (2023). Human brain organoid model of maternal immune activation identifies radial glia cells as selectively vulnerable. Mol. Psychiatry *28*, 5077–5089. https://doi.org/10.1038/s41380-023-01997-1.
74. Lee, Y., Ise, T., Ha, D., Saint Fleur, A., Hahn, Y., Liu, X.F., Nagata, S., Lee, B., Bera, T.K., and Pastan, I. (2006). Evolution and expression of chimeric POTE-actin genes in the human genome. Proc. Natl. Acad. Sci. USA *103*, 17885–17890. https://doi.org/10.1073/pnas.0608344103.
75. Korteling, D., Musch, J.L.I., Zinkstok, J.R., and Boot, E. (2024). Psychiatric and neurological manifestations in adults with Smith-Magenis syndrome: A scoping review. Am. J. Med. Genet. B Neuropsychiatr. Genet. *195*, e32956. https://doi.org/10.1002/ajmg.b.32956.
76. Arboleda, V.A., Lee, H., Parnaik, R., Fleming, A., Banerjee, A., Ferraz-de-Souza, B., Délot, E.C., Rodriguez-Fernandez, I. A., Braslavsky, D., Bergadá, I., et al. (2012). Mutations in the PCNA-binding domain of CDKN1C cause IMAGe syndrome. Nat. Genet. *44*, 788–792. https://doi.org/10.1038/ng.2275.
77. Barone, G., Staples, C.J., Ganesh, A., Patterson, K.W., Bryne, D.P., Myers, K.N., Patil, A.A., Eyers, C.E., Maslen, S., Skehel, J.M., et al. (2016). Human CDK18 promotes replication stress signaling and genome stability. Nucleic Acids Res. *44*, 8772–8785. https://doi.org/10.1093/nar/gkw615.
78. Di Leonardo, A., Linke, S.P., Clarkin, K., and Wahl, G.M. (1994). DNA damage triggers a prolonged p53-dependent G1 arrest and long-term induction of Cip1 in normal human fibroblasts. Genes Dev. *8*, 2540–2551. https://doi.org/10.1101/gad.8.21.2540.
79. Pasca, S.P. (2018). The rise of three-dimensional human brain cultures. Nature *553*, 437–445. https://doi.org/10.1038/nature25032.
80. Bayes, A., van de Lagemaat, L.N., Collins, M.O., Croning, M. D., Whittle, I.R., Choudhary, J.S., and Grant, S.G. (2011). Characterization of the proteome, diseases and evolution of the human postsynaptic density. Nat. Neurosci. *14*, 19–21. https://doi.org/10.1038/nn.2719.
81. Beaudoin, G.M.J., Schofield, C.M., Nuwal, T., Zang, K., Ullian, E.M., Huang, B., and Reichardt, L.F. (2012). Afadin, a Ras/Rap effector that controls cadherin function, promotes spine and excitatory synapse density in the hippocampus. J. Neurosci. *32*, 99–110. https://doi.org/10.1523/JNEUROSCI.4565-11.2012.
82. Kalinowska, M., Chávez, A.E., Lutzu, S., Castillo, P.E., Bukauskas, F.F., and Francesconi, A. (2015). Actinin-4 Governs Dendritic Spine Dynamics and Promotes Their Remodeling by Metabotropic Glutamate Receptors. J. Biol. Chem. *290*, 15909–15920. https://doi.org/10.1074/jbc.M115.640136.
83. Ing-Simmons, E., Vaid, R., Bing, X.Y., Levine, M., Mannervik, M., and Vaquerizas, J.M. (2021). Independence of chromatin conformation and gene regulation during Drosophila dorsoventral patterning. Nat. Genet. *53*, 487–499. https://doi.org/10.1038/s41588-021-00799-x.
84. Varrone, M., Nanni, L., Ciriello, G., and Ceri, S. (2020). Exploring chromatin conformation and gene co-expression through graph embedding. Bioinformatics *36*, i700–i708. https://doi.org/10.1093/bioinformatics/btaa803.
85. Beagan, J.A., Pastuzyn, E.D., Fernandez, L.R., Guo, M.H., Feng, K., Titus, K.R., Chandrashekar, H., Shepherd, J.D., and Phillips-Cremins, J.E. (2020). Three-dimensional genome restructuring across timescales of activity-induced neuronal gene expression. Nat. Neurosci. *23*, 707–717. https://doi.org/10.1038/s41593-020-0634-6.
86. Fernandez-Albert, J., Lipinski, M., Lopez-Cascales, M.T., Rowley, M.J., Martin-Gonzalez, A.M., Del Blanco, B., Corces, V.G., and Barco, A. (2019). Immediate and deferred epigenomic signatures of in vivo neuronal activation in mouse hippocampus. Nat. Neurosci. *22*, 1718–1730. https://doi.org/10.1038/s41593-019-0476-2.
87. Marco, A., Meharena, H.S., Dileep, V., Raju, R.M., Davila-Velderrain, J., Zhang, A.L., Adaikkan, C., Young, J.Z., Gao, F., Kellis, M., and Tsai, L.H. (2020). Mapping the epigenomic and transcriptomic interplay during memory formation and recall in the hippocampal engram ensemble. Nat. Neurosci. *23*, 1606–1617. https://doi.org/10.1038/s41593-020-00717-0.
88. Bilgic, M., Wu, Q., Suetsugu, T., Shitamukai, A., Tsunekawa, Y., Shimogori, T., Kadota, M., Nishimura, O., Kuraku, S., Kiyonari, H., and Matsuzaki, F. (2023). Truncated radial glia as a common precursor in the late corticogenesis of gyrencephalic mammals. eLife *12*, 91406. https://doi.org/10.7554/eLife.91406.
89. Shao, Z., Noh, H., Bin Kim, W., Ni, P., Nguyen, C., Cote, S.E., Noyes, E., Zhao, J., Parsons, T., Park, J.M., et al. (2019). Dysregulated protocadherin-pathway activity as an intrinsic defect in induced pluripotent stem cell-derived cortical interneurons from subjects with schizophrenia. Nat. Neurosci. *22*, 229–242. https://doi.org/10.1038/s41593-018-0313-z.

90. Handunnetthi, L., Saatci, D., Hamley, J.C., and Knight, J.C. (2021). Maternal immune activation downregulates schizophrenia genes in the foetal mouse brain. Brain Commun. *3*, fcab275. https://doi.org/10.1093/braincomms/fcab275.
91. Margot, H., Pizano, A., Amestoy, A., Lacombe, D., Berges, C., Beneteau, C., and Innes, A.M. (2024). Investigations of an individual with a Marfanoid habitus, mild intellectual disability, and severe social anxiety identifies PCDHGA5 as a candidate neurodevelopmental disorder gene. Am. J. Med. Genet. C Semin. Med. Genet. *196*, e32087. https://doi.org/10.1002/ajmg.c.32087.
92. Alagramam, K.N., Yuan, H., Kuehn, M.H., Murcia, C.L., Wayne, S., Srisailpathy, C.R., Lowry, R.B., Knaus, R., Van Laer, L., Bernier, F.P., et al. (2001). Mutations in the novel protocadherin PCDH15 cause Usher syndrome type 1F. Hum. Mol. Genet. *10*, 1709–1718. https://doi.org/10.1093/hmg/10.16.1709.
93. Georgieva, L., Rees, E., Moran, J.L., Chambert, K.D., Milanova, V., Craddock, N., Purcell, S., Sklar, P., McCarroll, S., Holmans, P., et al. (2014). De novo CNVs in bipolar affective disorder and schizophrenia. Hum. Mol. Genet. *23*, 6677–6683. https://doi.org/10.1093/hmg/ddu379.
94. Garrett, A.M., Schreiner, D., Lobas, M.A., and Weiner, J.A. (2012). gamma-protocadherins control cortical dendrite arborization by regulating the activity of a FAK/PKC/MARCKS signaling pathway. Neuron *74*, 269–276. https://doi.org/10.1016/j.neuron.2012.01.028.
95. Rakotomamonjy, J., Rylaarsdam, L., Fares-Taie, L., McDermott, S., Davies, D., Yang, G., Fagbemi, F., Epstein, M., Fairbanks-Santana, M., Rozet, J.M., and Guemez-Gamboa, A. (2023). PCDH12 loss results in premature neuronal differentiation and impeded migration in a cortical organoid model. Cell Rep. *42*, 112845. https://doi.org/10.1016/j.celrep.2023.112845.
96. Styner, M., Lieberman, J.A., McClure, R.K., Weinberger, D. R., Jones, D.W., and Gerig, G. (2005). Morphometric analysis of lateral ventricles in schizophrenia and healthy controls regarding genetic and disease-specific factors. Proc. Natl. Acad. Sci. USA *102*, 4872–4877. https://doi.org/10.1073/pnas.0501117102.
97. Miyagi, S., Masui, S., Niwa, H., Saito, T., Shimazaki, T., Okano, H., Nishimoto, M., Muramatsu, M., Iwama, A., and Okuda, A. (2008). Consequence of the loss of Sox2 in the developing brain of the mouse. FEBS Lett. *582*, 2811–2815. https://doi.org/10.1016/j.febslet.2008.07.011.
98. Pringsheim, M., Mitter, D., Schröder, S., Warthemann, R., Plümacher, K., Kluger, G., Baethmann, M., Bast, T., Braun, S., Büttel, H.M., et al. (2019). Structural brain anomalies in patients with FOXG1 syndrome and in Foxg1+/- mice. Ann. Clin. Transl. Neurol. *6*, 655–668. https://doi.org/10.1002/acn3.735.
99. Fetit, R., Barbato, M.I., Theil, T., Pratt, T., and Price, D.J. (2023). 16p11.2 deletion accelerates subpallial maturation and increases variability in human iPSC-derived ventral telencephalic organoids. Development *150*, dev201227. https://doi.org/10.1242/dev.201227.
100. Cobolli Gigli, C., Scaramuzza, L., De Simone, M., Rossi, R.L., Pozzi, D., Pagani, M., Landsberger, N., and Bedogni, F. (2018). Lack of Methyl-CpG Binding Protein 2 (MeCP2) Affects Cell Fate Refinement During Embryonic Cortical Development. Cereb. Cortex *28*, 1846–1856. https://doi.org/10.1093/cercor/bhx360.
101. Nowakowski, T.J., Pollen, A.A., Sandoval-Espinosa, C., and Kriegstein, A.R. (2016). Transformation of the Radial Glia Scaffold Demarcates Two Stages of Human Cerebral Cortex Development. Neuron *91*, 1219–1227. https://doi.org/10.1016/j.neuron.2016.09.005.
102. Qazi, S., Jit, B.P., Das, A., Karthikeyan, M., Saxena, A., Ray, M.D., Singh, A.R., Raza, K., Jayaram, B., and Sharma, A. (2022). BESFA: bioinformatics based evolutionary, structural & functional analysis of prostrate, Placenta, Ovary, Testis, and Embryo (POTE) paralogs. Heliyon *8*, e10476. https://doi.org/10.1016/j.heliyon.2022.e10476.
103. Wonkam, A., Adadey, S.M., Schrauwen, I., Aboagye, E.T., Wonkam-Tingang, E., Esoh, K., Popel, K., Manyisa, N., Jonas, M., deKock, C., et al. (2022). Exome sequencing of families from Ghana reveals known and candidate hearing impairment genes. Commun. Biol. *5*, 369. https://doi.org/10.1038/s42003-022-03326-8.
104. Brendal, M.A., King, K.A., Zalewski, C.K., Finucane, B.M., Introne, W., Brewer, C.C., and Smith, A.C.M. (2017). Auditory Phenotype of Smith-Magenis Syndrome. J. Speech Lang. Hear. Res. *60*, 1076–1087. https://doi.org/10.1044/2016_JSLHR-H-16-0024.
105. Goldman, A.M., Potocki, L., Walz, K., Lynch, J.K., Glaze, D. G., Lupski, J.R., and Noebels, J.L. (2006). Epilepsy and chromosomal rearrangements in Smith-Magenis Syndrome [del (17)(p11.2p11.2)]. J. Child Neurol. *21*, 93–98. https://doi.org/10.1177/08830738060210021201.
106. Hu, C., Tao, L., Cao, X., and Chen, L. (2020). The solute carrier transporters and the brain: Physiological and pharmacological implications. Asian J. Pharm. Sci. *15*, 131–144. https://doi.org/10.1016/j.ajps.2019.09.002.
107. Ayka, A., and Şehirli, A.Ö. (2020). The Role of the SLC Transporters Protein in the Neurodegenerative Disorders. Clin. Psychopharmacol. Neurosci. *18*, 174–187. https://doi.org/10.9758/cpn.2020.18.2.174.
108. Dai, G., Yu, H., Kruse, M., Traynor-Kaplan, A., and Hille, B. (2016). Osmoregulatory inositol transporter SMIT1 modulates electrical activity by adjusting PI(4,5)P2 levels. Proc. Natl. Acad. Sci. USA *113*, E3290–E3299. https://doi.org/10.1073/pnas.1606348113.
109. Morales, M.J., Castellino, R.C., Crews, A.L., Rasmusson, R. L., and Strauss, H.C. (1995). A novel beta subunit increases rate of inactivation of specific voltage-gated potassium channel alpha subunits. J. Biol. Chem. *270*, 6272–6277. https://doi.org/10.1074/jbc.270.11.6272.
110. Kathuria, A., Lopez-Lengowski, K., Watmuff, B., and Karmacharya, R. (2020). Comparative Transcriptomic Analysis of Cerebral Organoids and Cortical Neuron Cultures Derived from Human Induced Pluripotent Stem Cells. Stem Cells Dev. *29*, 1370–1381. https://doi.org/10.1089/scd.2020.0069.

The American Journal of Human Genetics, Volume *112*

Supplemental information

Molecular and developmental deficits in Smith-Magenis syndrome human stem cell-derived cortical neural models

Yu-Ju Lee, Ya-Ting Chang, Yoobin Cho, Max Kowalczyk, Adrian Dragoiescu, Alain Pacis, Senthilkumar Kailasam, François Lefebvre, Qihuang Zhang, Xiaojing Gao, and Wei-Hsiang Huang

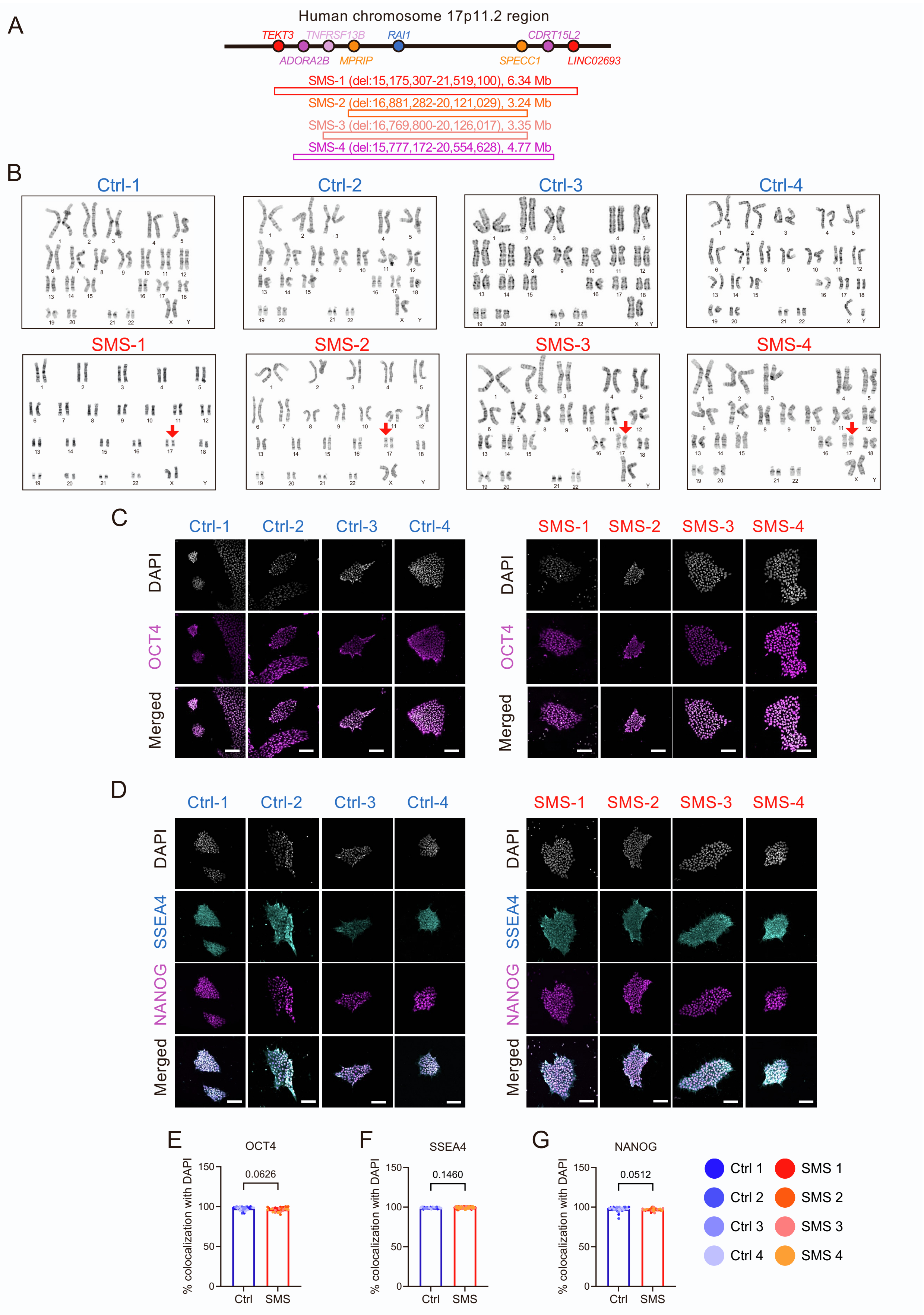

A
Human chromosome 17p11.2 region
TEKT3
TNFRSF13B
RAI1
CDRT15L2
ADORA2B
MPRIP
SPECC1
LINC02693
SMS-1 (del:15,175,307-21,519,100), 6.34 Mb
SMS-2 (del:16,881,282-20,121,029), 3.24 Mb
SMS-3 (del:16,769,800-20,126,017), 3.35 Mb
SMS-4 (del:15,777,172-20,554,628), 4.77 Mb
B
Ctrl-1
Ctrl-2
Ctrl-3
Ctrl-4
SMS-1
SMS-2
SMS-3
SMS-4
C
DAPI
OCT4
Merged
D
SSEA4
NANOG
E
OCT4
F
SSEA4
G
NANOG
% colocalization with DAPI
0.0626
0.1460
0.0512
Ctrl
SMS
Ctrl 1
Ctrl 2
Ctrl 3
Ctrl 4
SMS 1
SMS 2
SMS 3
SMS 4

Figure S1: Characterization of Ctrl and SMS hiPSCs

(A) Schematic diagram showing del(17)p11.2 in four clinically diagnosed SMS patients, including the boundary genes deleted at both extremities of the CNV. From left to right: Tektin 3 (*TEKT3*); Adenosine A2b receptor (*ADORA2B*); TNF receptor superfamily member 13B (*TNFRSF13B*); Myosin phosphatase Rho interacting protein (*MPRIP*); Sperm antigen with calponin homology and coiled-coil domains 1 (*SPECC1*); CMT1A duplicated region transcript 15-like 2 (*CDRT15L2*); Long intergenic non-protein coding RNA 2693 (*LINC02693*). *RAI1* (in blue) is deleted in all four patients.

(B) Karyograms of hiPSCs showing normal chromosomes in all lines except del(17)p11.2 (red arrows) in SMS lines. Note that all samples (except Ctrl-4) were derived from female individuals.

(C) Representative images of Ctrl and SMS hiPSC colonies stained with a pluripotency marker OCT4 (magenta). DAPI-labeled nuclei are in grey. Scale bars: 100µm.

(D) Representative images of Ctrl and SMS hiPSC colonies stained with pluripotency markers SSEA4 (cyan) and NANOG (magenta). DAPI-labeled nuclei are in grey. Scale bars: 100µm.

(E) Quantification of OCT4 in Ctrl and SMS hiPSCs. 32 hiPSC colonies per genotype with 8 colonies per cell line. Each dot represents one colony. $U = 373$, p=0.0626.

(F) Quantification of SSEA4 in Ctrl and SMS hiPSCs. 32 hiPSC colonies per genotype with 8 colonies per cell line. Each dot represents one colony. $U = 405.5$, p=0.1460.

(G) Quantification of NANOG in Ctrl and SMS hiPSCs. 32 hiPSC colonies per genotype with 8 colonies per cell line. Each dot represents one colony. $U = 367$, p=0.0512.

Data presented as mean ± S.E.M.; (**E-G**) U- and p-values by two-tailed Mann-Whitney tests.

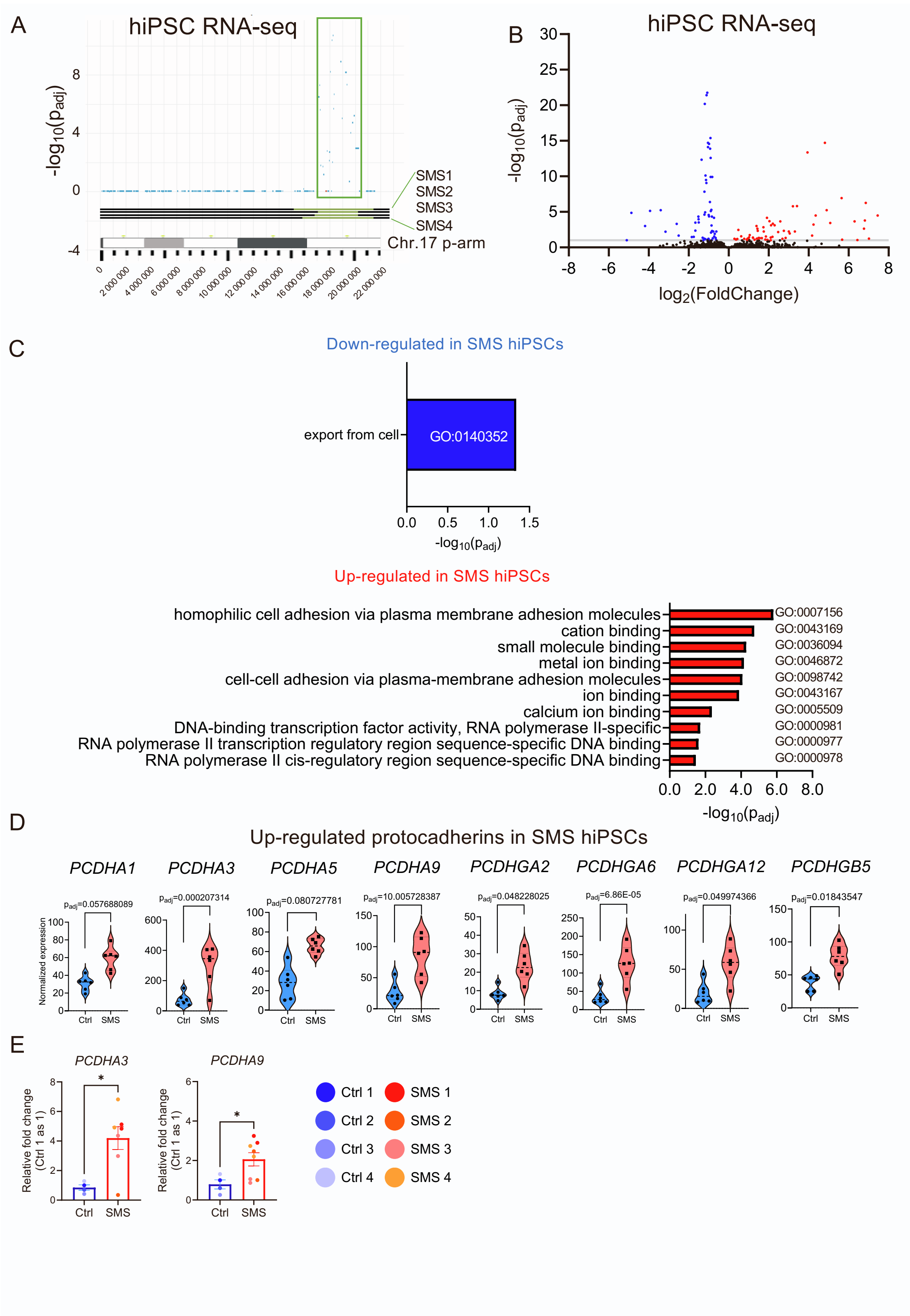
A
hiPSC RNA-seq
-log10(padj)
SMS1
SMS2
SMS3
SMS4
Chr.17 p-arm
B
hiPSC RNA-seq
-log10(padj)
log2(FoldChange)
C
Down-regulated in SMS hiPSCs
export from cell
GO:0140352
-log10(padj)
Up-regulated in SMS hiPSCs
homophilic cell adhesion via plasma membrane adhesion molecules
cation binding
small molecule binding
metal ion binding
cell-cell adhesion via plasma-membrane adhesion molecules
ion binding
calcium ion binding
DNA-binding transcription factor activity, RNA polymerase II-specific
RNA polymerase II transcription regulatory region sequence-specific DNA binding
RNA polymerase II cis-regulatory region sequence-specific DNA binding
GO:0007156
GO:0043169
GO:0036094
GO:0046872
GO:0098742
GO:0043167
GO:0005509
GO:0000981
GO:0000977
GO:0000978
-log10(padj)
D
Up-regulated protocadherins in SMS hiPSCs
PCDHA1
PCDHA3
PCDHA5
PCDHA9
PCDHGA2
PCDHGA6
PCDHGA12
PCDHGB5
padj=0.057688089
padj=0.000207314
padj=0.080727781
padj=10.005728387
padj=0.048228025
padj=6.86E-05
padj=0.049974366
padj=0.01843547
Normalized expression
Ctrl
SMS
E
PCDHA3
PCDHA9
Relative fold change (Ctrl 1 as 1)
Ctrl
SMS
Ctrl 1
Ctrl 2
Ctrl 3
Ctrl 4
SMS 1
SMS 2
SMS 3
SMS 4

Figure S2: RNA-sequencing showing dysregulation of genes encoding organismal development and cell adhesion molecules in SMS hiPSCs

(A) A Manhattan plot displaying the genomic landscape of significantly downregulated genes on the chromosome 17 p-arm in SMS hiPSCs (compared to Ctrl hiPSCs) based on -$\log_{10}$ transformed p_{adj} derived from a one-sided Wald test. The x-axis represents the genomic position, and the y-axis corresponds to the significance level of differential expression. The blue dots represent individual genes, with their positions on the x-axis representing their location on chromosome 17p and their heights on the y-axis indicating the significance of their differential expressions. The dark horizontal lines at the bottom indicate chromosomes, with the green segments corresponding to the regions subjected to del(17)p11.2 in SMS hiPSCs.

(B) A Volcano plot showing the global transcriptomic changes when comparing SMS with Ctrl hiPSCs. Each dot represents a gene. The $\log_2$ fold change of each gene is represented on the x-axis and the -$\log_{10}$ of its $\mathbf{p_{adj}}$ is on the y-axis. Up-regulated genes in SMS hiPSCs with p_{adj} less than 0.1 are indicated by red dots. Down-regulated genes in SMS hiPSCs with p_{adj} less than 0.1 are indicated by blue dots. The grey line indicates p_{adj}=0.1.

(C) GO analysis showing that cell export genes are down-regulated in SMS hiPSCs and cell adhesion molecules are up-regulated in SMS hiPSCs. The GO terms for down-regulated genes (in blue) and up-regulated genes (in red) and the respective -$\log_{10}(p_{adj})$ are shown.

(D) Violin plots illustrating that bulk RNA-seq detected up-regulated protocadherin genes in SMS hiPSCs when compared to Ctrls. Each dot represents a sample. p_{adj} by Wald test.

(E) Quantitative RT-PCR experiments confirming altered expression of *PCDHA3* (t=3.181, df=9, p=0.0112) and *PCDHA9* (t=2.487, df=10, p=0.0322) in SMS hiPSCs when compared to Ctrls (relative to Ctrl 1). t-ratios, df, and p-values by two-tailed unpaired t-tests.

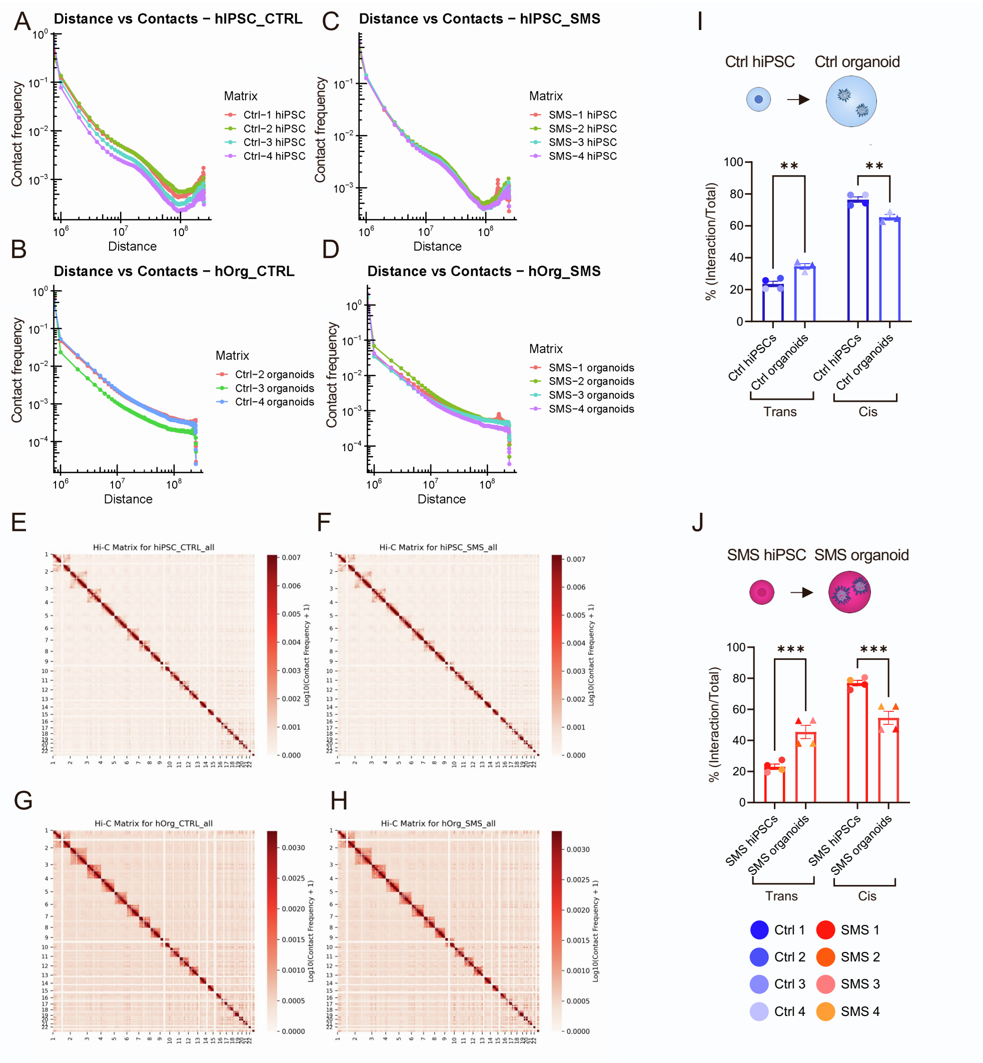

Figure S3: Quality control of Hi-C data obtained from Ctrl and SMS hiPSCs and cortical organoid models at day 75 differentiation

(A-D) Plots showing interaction frequency decay as a function of the genomic distance. Normalized Hi-C data binned at 250 kb were converted to contact frequency datasets. The X-axis indicates distance and the Y-axis indicates contact frequencies (both in log_{10} scales). Shown are the decay plots for Ctrl hiPSC (**A**), Ctrl cortical organoids (**B**), SMS hiPSC (**C**), and SMS cortical organoids (**D**). Note that the Ctrl-1 organoid library was removed due to insufficient data quality.

(E-H) Interchromosomal contact maps in Ctrl hiPSCs (1-4 combined, **E**), SMS hiPSCs (1-4 combined, **F**), Ctrl cortical organoids (2-4 combined, **G**), and SMS cortical organoids (1-4 combined, **H**).

(I-J) Percent distribution of trans- and cis-chromosomal interactions during in vitro corticogenesis from hiPSCs to organoids for Ctrl (**I**, $F(1,10)=38.8$, $p=0.0027$) and SMS (**J**, $F(1,12)=47.19$, $p=0.0008$) samples. F(DFn, DFd) and *p*-values were calculated by two-way ANOVA with post hoc Šídák multiple comparisons. Ctrl and SMS organoids showed increased trans-interactions and decreased cis-interactions compared to corresponding hiPSCs. Circles represent hiPSCs and triangles represent organoids.

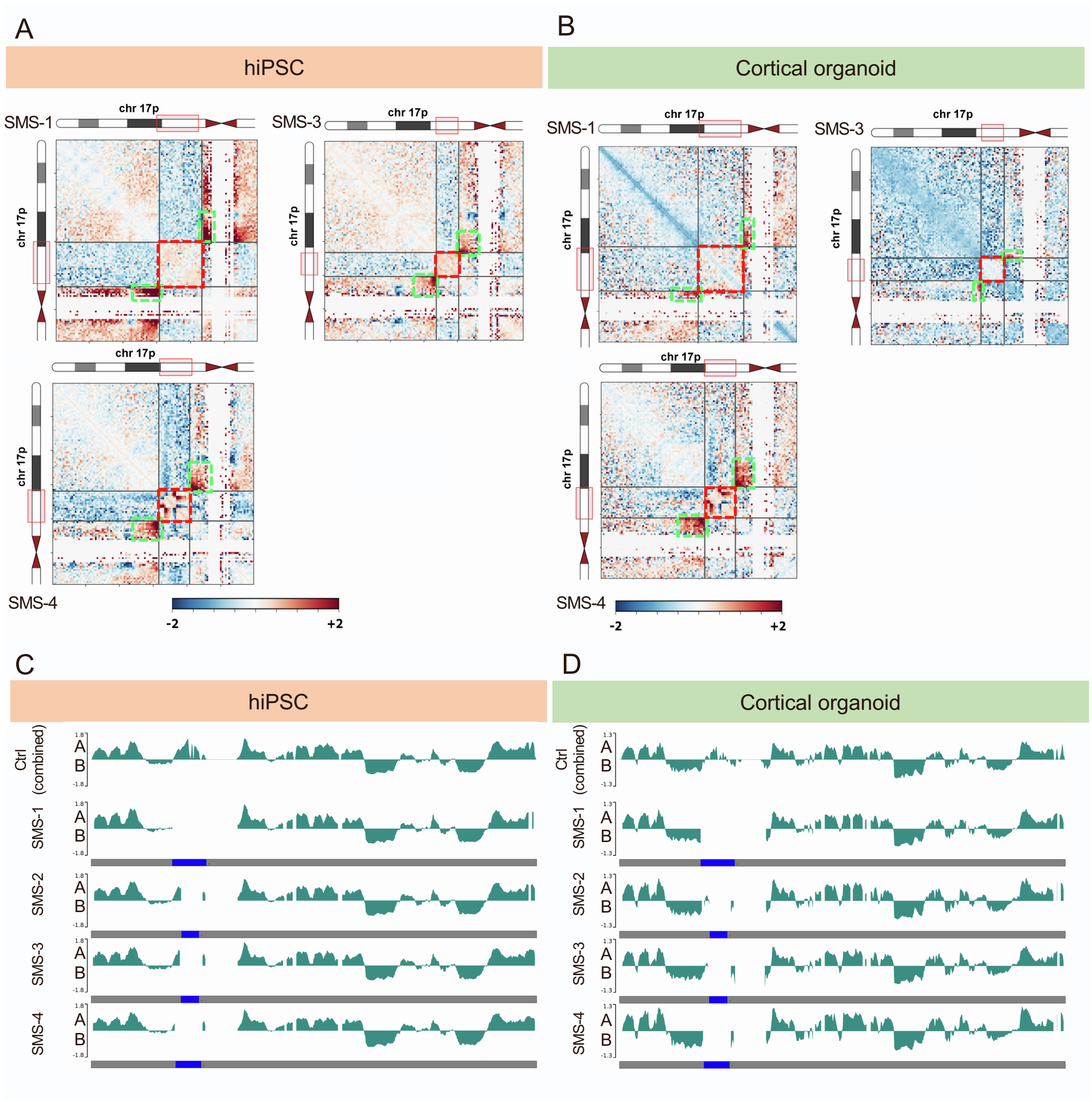

Figure S4: Hi-C data obtained from Ctrl and SMS hiPSCs and cortical organoid models at day 75 differentiation

(A-B) Heatmaps showing increased cis-contacts within del(17)p11.2 deletion boundaries (red dashed boxes) and between the DNA regions flanking del(17)p11.2 (green dashed boxes) in SMS hiPSCs (**A**) and SMS cortical organoids (**B**) compared to corresponding Ctrl samples.

(C-D) A/B compartments identified on chromosome 17 in Ctrl hiPSCs (1-4 combined) and SMS 1-4 hiPSC (**C**) and in Ctrl cortical organoids (2-4 combined) and SMS 1-4 cortical organoids (**D**). Del(17)p11.2 regions in each SMS line are indicated by blue boxes at the bottom. No A/B switching was observed in SMS lines.

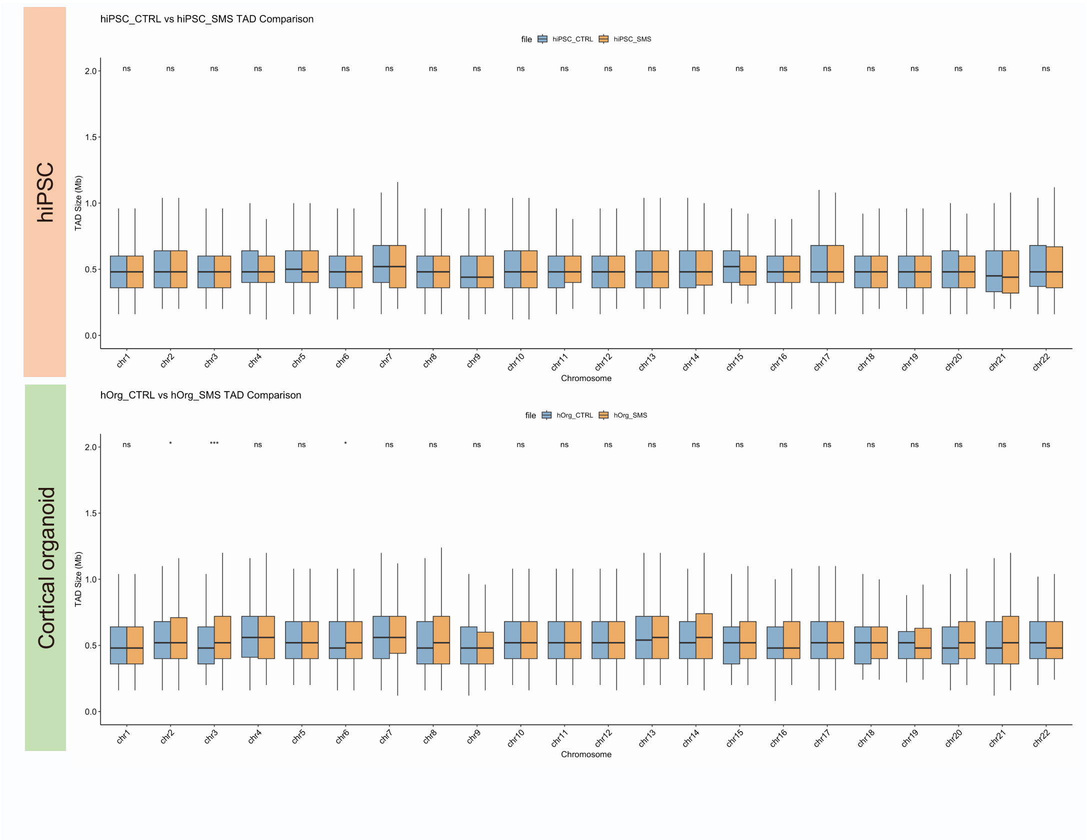

Figure S5: Comparison of TAD sizes in Ctrl and SMS hiPSCs and cortical organoid models at day 75 differentiation

Averaged TAD size (Mb) in Ctrl hiPSCs (1-4 combined) versus SMS hiPSCs (1-4 combined) (top) and Ctrl cortical organoids (2-4 combined) versus SMS cortical organoids (1-4 combined) (bottom). At this scale, no significant differences in TAD sizes were observed in most pairs of autosomes (ns= p >0.05, two-tailed t-tests).

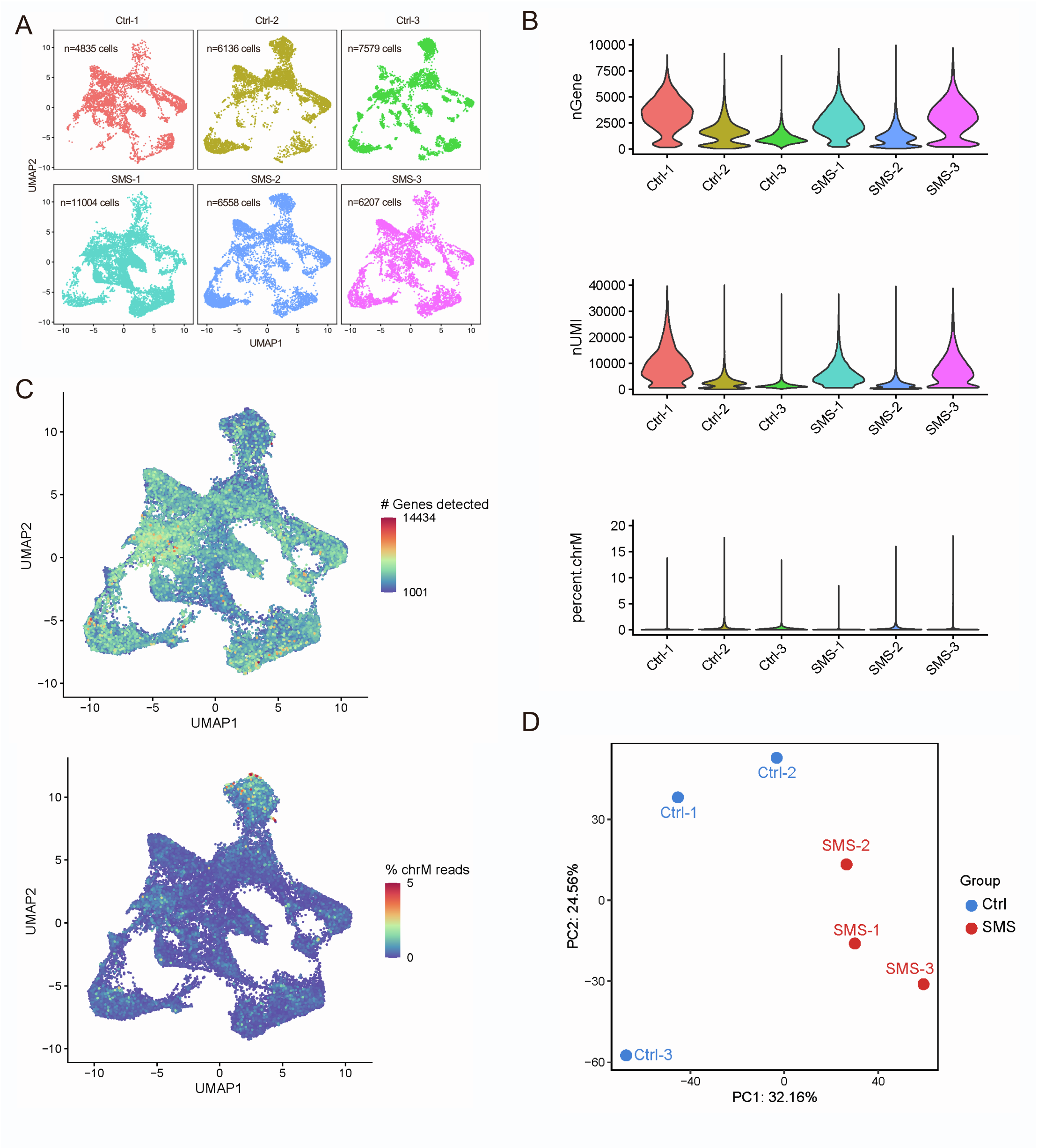

Figure S6: Quality control of snRNA-seq data from Ctrl and SMS hiPSCs-derived cortical organoids at day 75 differentiation

(A-D) Quality control of snRNA-seq data, including (**A**), the number of cells harvested from each line (**B**), the number of detected genes (top), the number of unique molecular identifiers (UMIs) (middle), and the percentage of UMIs assigned to mitochondrial genes (bottom) per cell for each line. **(C)** UMAP plot of all cells color-coded by number of genes detected and the proportion of mitochondrial reads. **(D)** PCA analysis showing that the pseudo-bulked transcriptomes of Ctrl-1-3 cortical organoids were separated from SMS-1-3 cortical organoids.

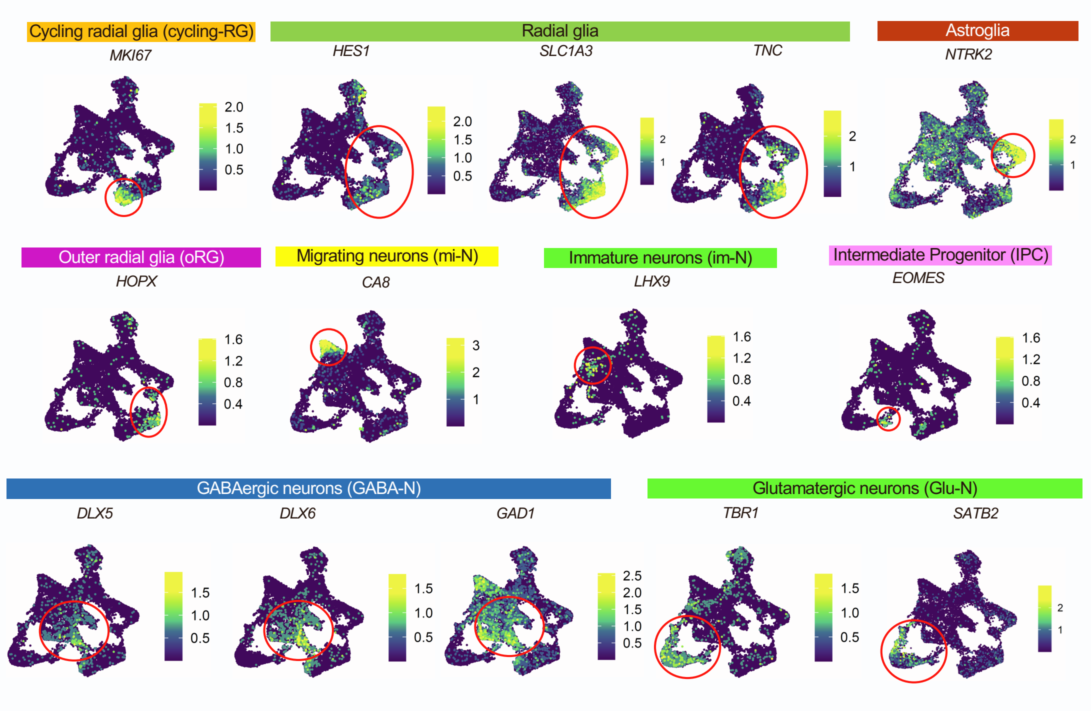

Figure S7: Cell type classification using snRNA-seq data from Ctrl and SMS hiPSCs-derived cortical organoids at day 75 differentiation

UMAP visualization of all cell clusters for additional markers indicated with red circles. *MKI67* shows actively dividing cycling-RGs, radial glial progenitors are marked by *HES1*, *SLC1A3*, and *TNC*, astroglia expresses high levels of *NTRK2*, outer radial glia (oRG) is labelled by *HOPX*, *CA8* expression is enriched in migrating neurons (mi-N), *LHX9* is expressed in immature neurons (im-N), intermediate progenitor cells (IPC) express *EOMES*, multiple GABAergic neuronal groups (GABA-N) express *DLX5*, *DLX6*, and *GAD1*, and glutamatergic neurons (Glu-N) are defined by *TBR1* and *SATB2* expression. Note that *TBR1* and *GAD1* are also expressed in some tRG, immature neurons, and migrating neurons that likely become Glu-N or GABA-N.

A

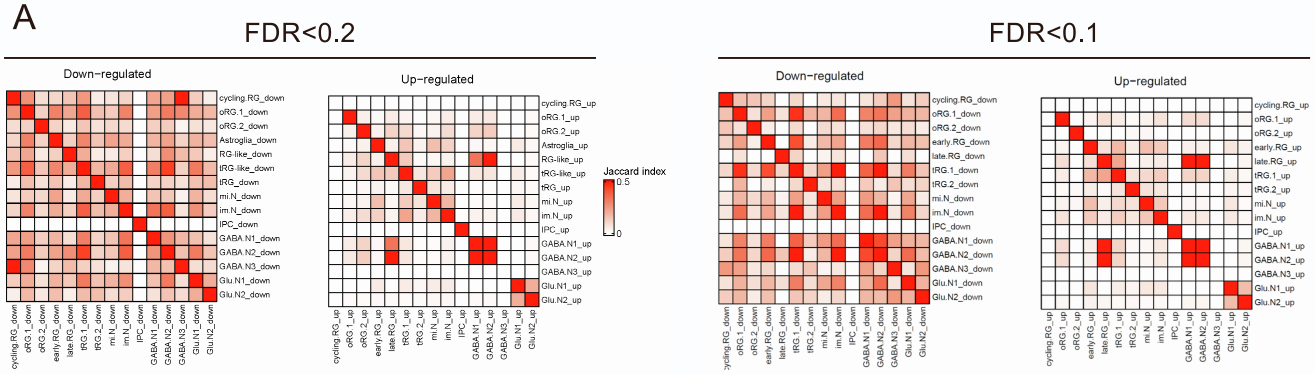

B

DEG number	Cycling-RG	Early RG	GABA-N1	GABA-N2	GABA-N3	Glu-N1	Glu-N2	im-N	IPC	Late-RG	mi-N	oRG-1	oRG-2	tRG-1	tRG-2
FDR<0.2	8	77	37	19	7	128	96	72	5	10	149	47	48	48	187
FDR<0.1	4	42	26	15	4	84	51	46	3	5	93	37	24	30	90

C

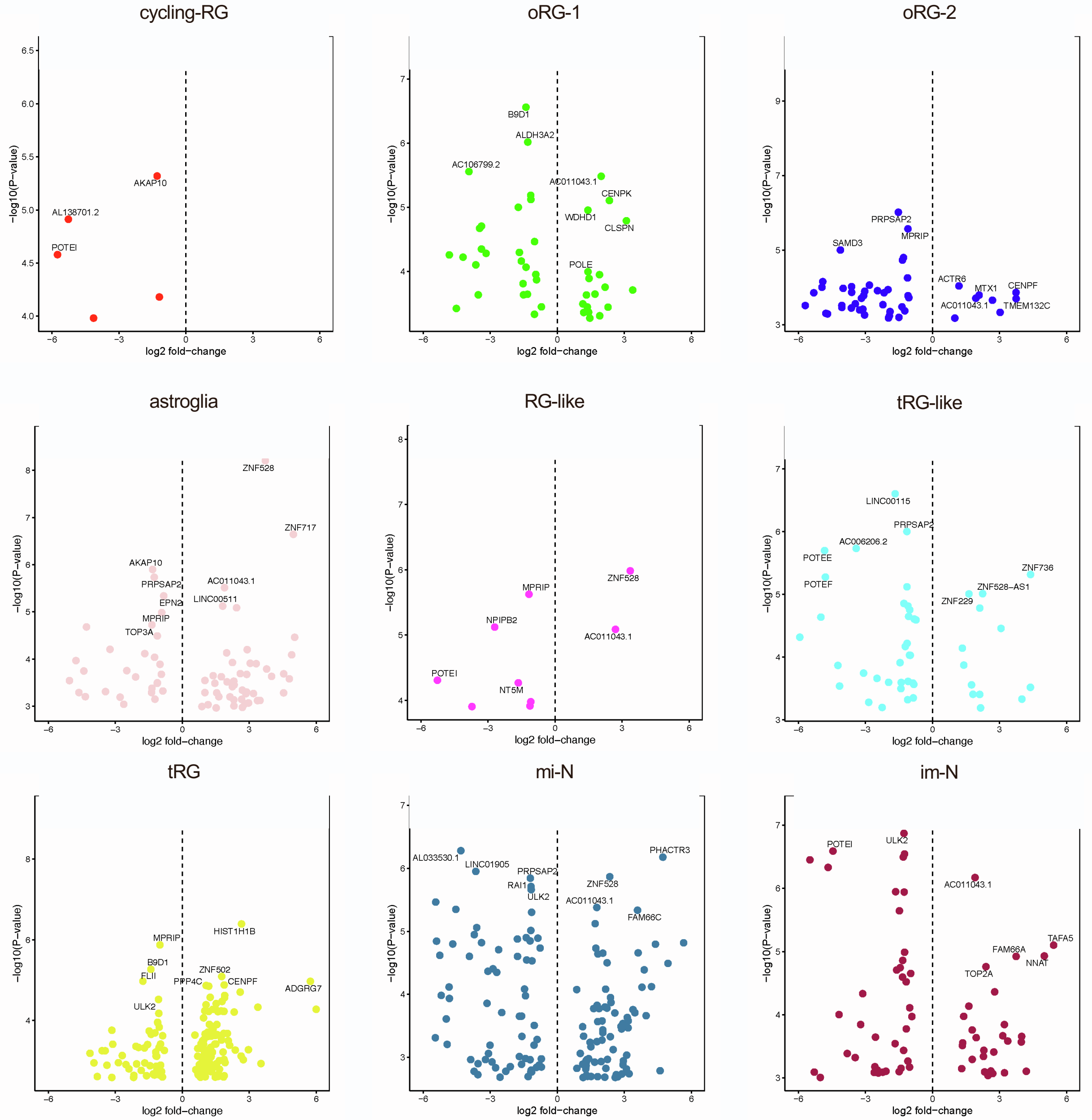

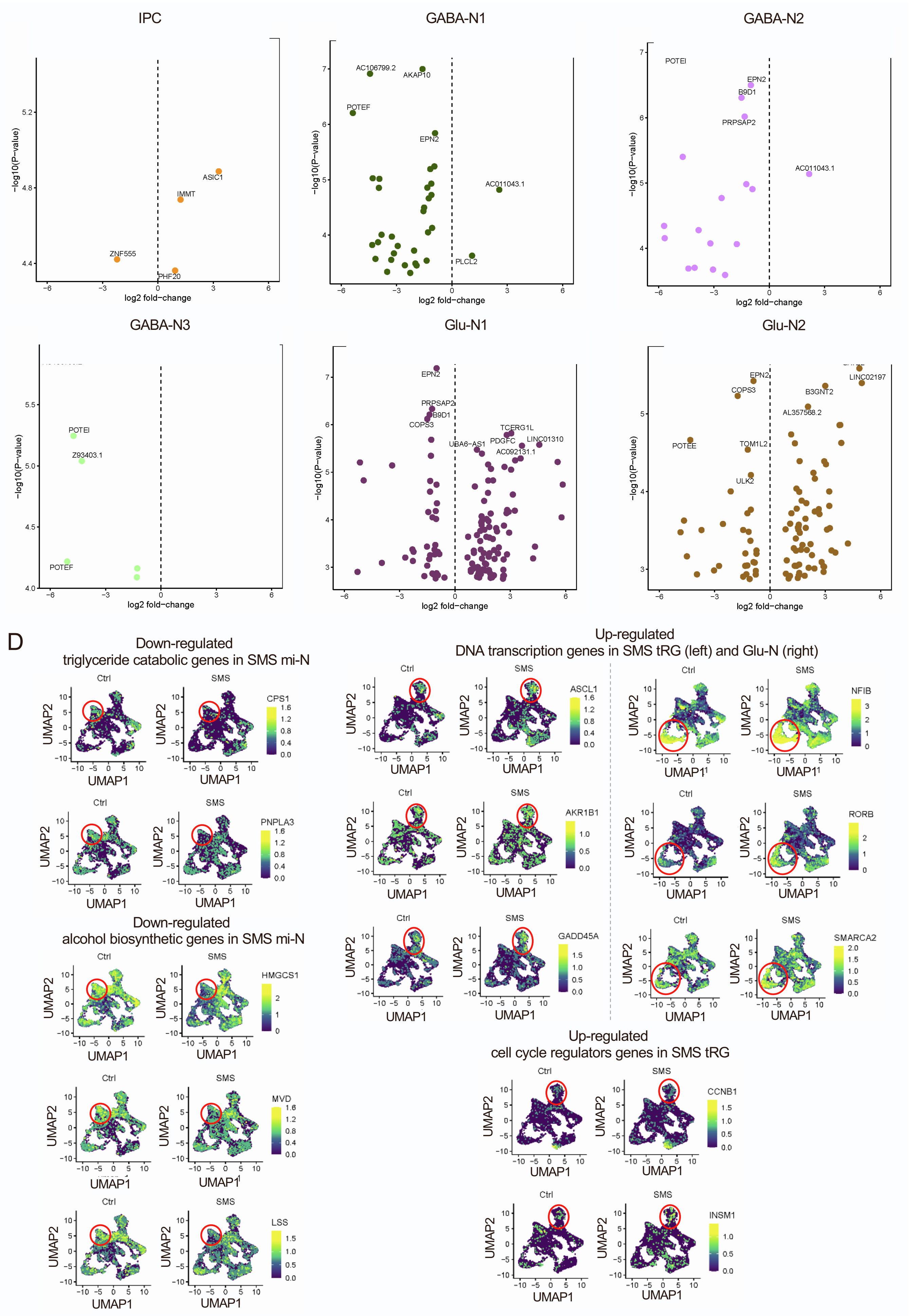
IPC
GABA-N1
GABA-N2
GABA-N3
Glu-N1
Glu-N2
−log10(P−value)
log2 fold−change
ASIC1
IMMT
ZNF555
PHF20
AC106799.2
AKAP10
POTEF
EPN2
AC011043.1
PLCL2
POTEI
B9D1
PRPSAP2
Z93403.1
COPS3
UBA6−AS1
PDGFC
TCERG1L
LINC01310
AC092131.1
LINC02197
B3GNT2
AL357568.2
POTEE
TOM1L2
ULK2
D
Down-regulated
triglyceride catabolic genes in SMS mi-N
Ctrl
SMS
UMAP1
UMAP2
CPS1
PNPLA3
Down-regulated
alcohol biosynthetic genes in SMS mi-N
HMGCS1
MVD
LSS
Up-regulated
DNA transcription genes in SMS tRG (left) and Glu-N (right)
ASCL1
AKR1B1
GADD45A
NFIB
RORB
SMARCA2
Up-regulated
cell cycle regulators genes in SMS tRG
CCNB1
INSM1

Figure S8: Differential gene expression analysis of snRNA-seq data from Ctrl and SMS hiPSCs-derived cortical organoids at day 75 differentiation

(A) Heatmaps showing similarity levels (Jaccard similarity index) of up-regulated and down-regulated genes between different cell clusters in cortical organoids, even after removing the del(17)p11.2 genes. A Jaccard similarity coefficient of 0 indicates no overlap and 1 indicates complete overlap. Left panels: FDR<0.2; Right panels: FDR<0.1.

(B) The number of DEGs in each cell cluster using FDR <0.2 or FDR<0.1.

(C) Volcano plots showing DEGs in each cell cluster ($p_{adj} < 0.2$). The representations are as follows: x-axis, $\log_2$ fold-change; y-axis, $-\log_{10}$ of a *p*-value.

(D) UMAP visualization of down-regulated catabolic genes in SMS mi-N (upper left), down-regulated alcohol biosynthetic genes in SMS mi-N (lower left), up-regulated DNA transcription genes in SMS tRG and Glu-N (upper right), and up-regulated cell cycle regulator genes in SMS tRG (lower right).

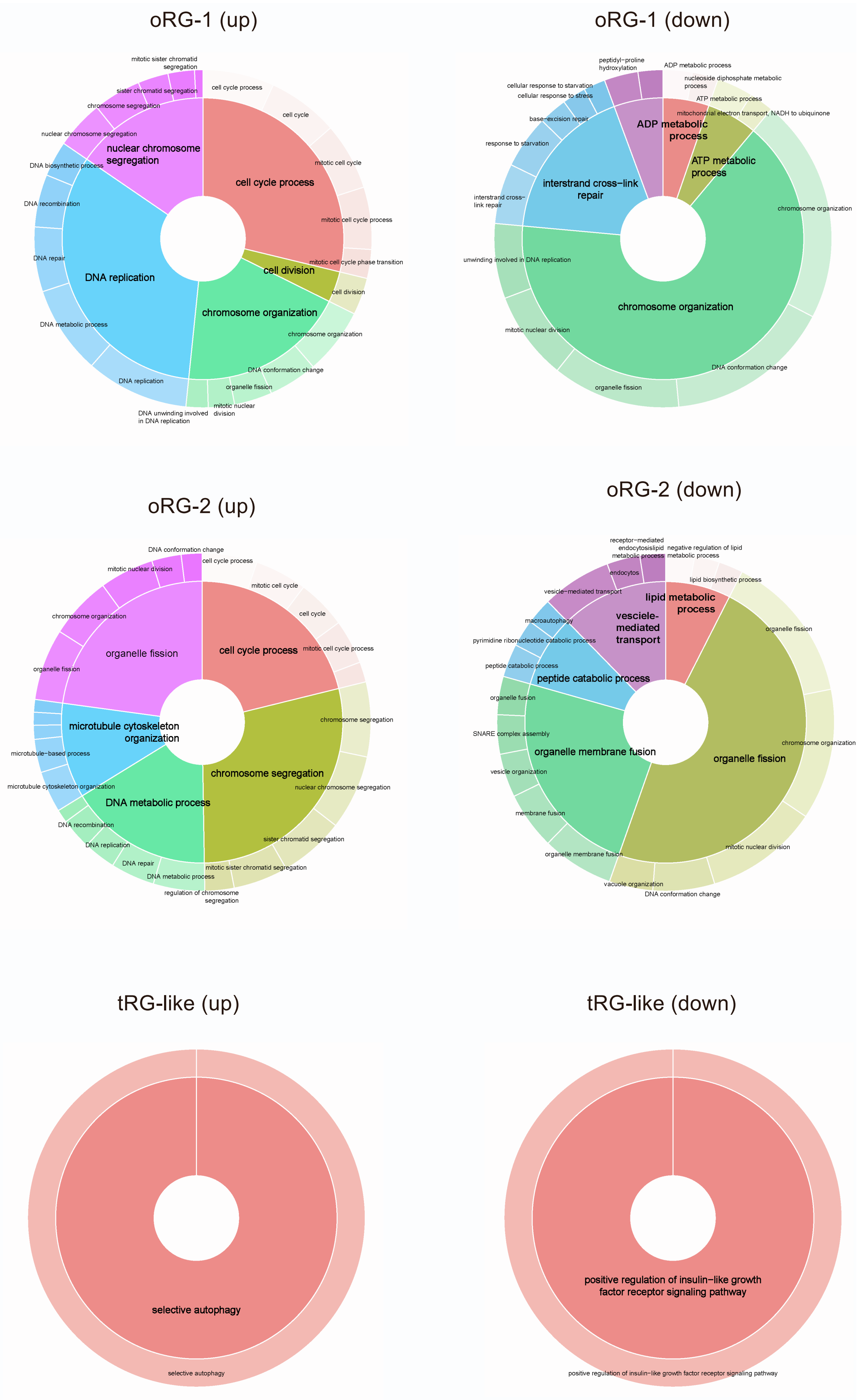
oRG-1 (up)
mitotic sister chromatid segregation
sister chromatid segregation
chromosome segregation
nuclear chromosome segregation
DNA biosynthetic process
DNA recombination
DNA repair
DNA metabolic process
DNA replication
DNA unwinding involved in DNA replication
mitotic nuclear division
organelle fission
DNA conformation change
chromosome organization
cell division
mitotic cell cycle phase transition
mitotic cell cycle process
mitotic cell cycle
cell cycle
cell cycle process
nuclear chromosome segregation
DNA replication
chromosome organization
oRG-1 (down)
peptidyl−proline hydroxylation
ADP metabolic process
nucleoside diphosphate metabolic process
ATP metabolic process
mitochondrial electron transport, NADH to ubiquinone
cellular response to starvation
cellular response to stress
base−excision repair
response to starvation
interstrand cross−link repair
unwinding involved in DNA replication
mitotic nuclear division
organelle fission
DNA conformation change
chromosome organization
ADP metabolic process
ATP metabolic process
interstrand cross−link repair
oRG-2 (up)
DNA conformation change
cell cycle process
mitotic nuclear division
mitotic cell cycle
cell cycle
chromosome organization
mitotic cell cycle process
organelle fission
chromosome segregation
microtubule−based process
microtubule cytoskeleton organization
nuclear chromosome segregation
DNA recombination
DNA replication
DNA repair
DNA metabolic process
sister chromatid segregation
mitotic sister chromatid segregation
regulation of chromosome segregation
organelle fission
microtubule cytoskeleton organization
DNA metabolic process
oRG-2 (down)
receptor−mediated endocytosis
lipid metabolic process
negative regulation of lipid metabolic process
endocytosis
lipid biosynthetic process
vesicle−mediated transport
macroautophagy
organelle fission
pyrimidine ribonucleotide catabolic process
peptide catabolic process
organelle fusion
SNARE complex assembly
vesicle organization
membrane fusion
organelle membrane fusion
vacuole organization
DNA conformation change
mitotic nuclear division
chromosome organization
lipid metabolic process
vesicle-mediated transport
peptide catabolic process
organelle membrane fusion
tRG-like (up)
selective autophagy
selective autophagy
tRG-like (down)
positive regulation of insulin−like growth factor receptor signaling pathway
positive regulation of insulin−like growth factor receptor signaling pathway

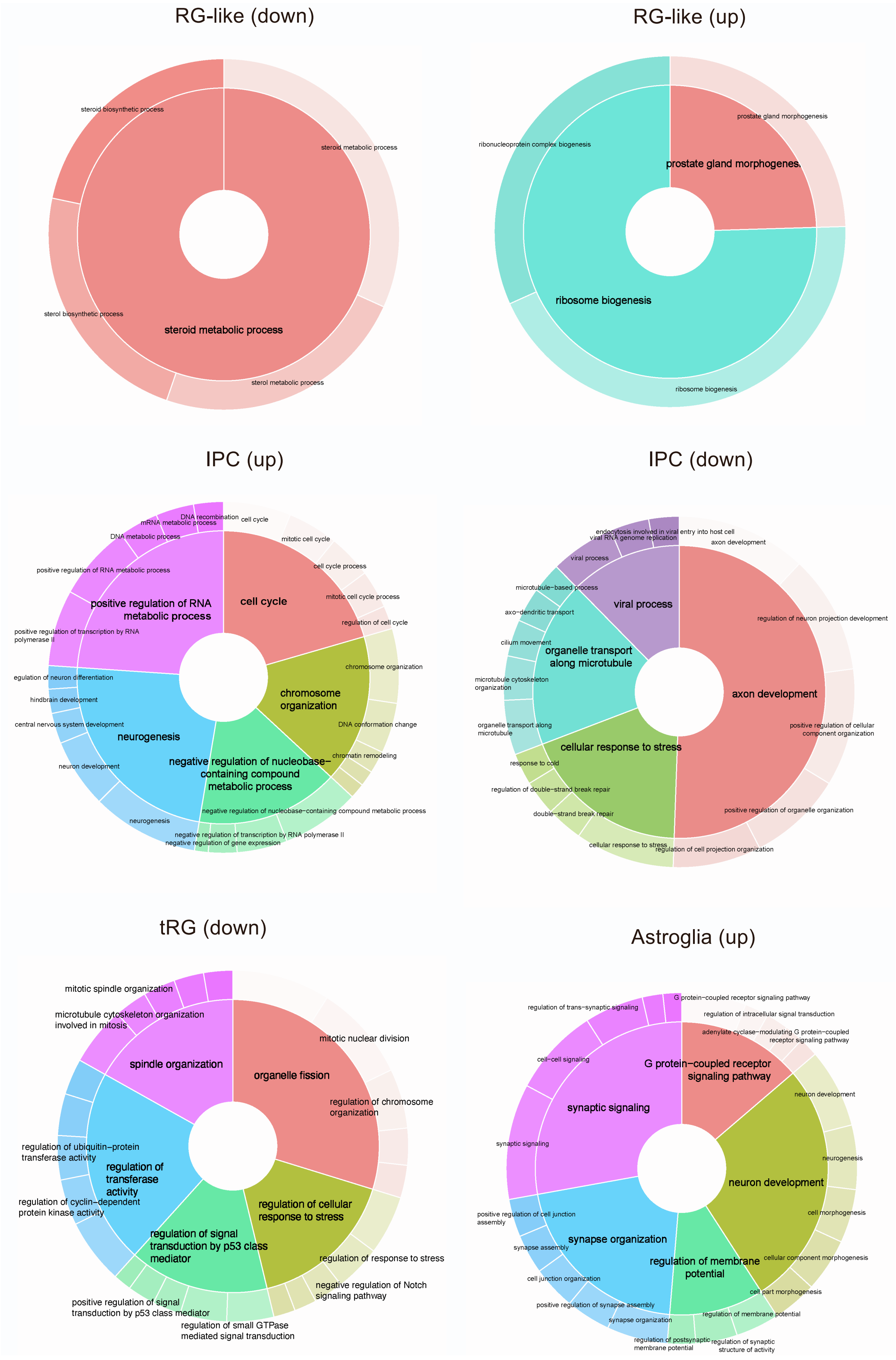

RG-like (down)
steroid biosynthetic process
steroid metabolic process
sterol biosynthetic process
steroid metabolic process
sterol metabolic process
RG-like (up)
ribonucleoprotein complex biogenesis
prostate gland morphogenesis
prostate gland morphogenes
ribosome biogenesis
ribosome biogenesis
IPC (up)
DNA recombination
mRNA metabolic process
cell cycle
DNA metabolic process
mitotic cell cycle
cell cycle process
positive regulation of RNA metabolic process
mitotic cell cycle process
cell cycle
positive regulation of RNA metabolic process
regulation of cell cycle
positive regulation of transcription by RNA polymerase II
chromosome organization
egulation of neuron differentiation
chromosome organization
hindbrain development
DNA conformation change
central nervous system development
neurogenesis
chromatin remodeling
negative regulation of nucleobase−containing compound metabolic process
neuron development
negative regulation of nucleobase−containing compound metabolic process
neurogenesis
negative regulation of transcription by RNA polymerase II
negative regulation of gene expression
IPC (down)
endocytosis involved in viral entry into host cell
viral RNA genome replication
axon development
viral process
microtubule−based process
viral process
axo−dendritic transport
regulation of neuron projection development
cilium movement
organelle transport along microtubule
microtubule cytoskeleton organization
axon development
organelle transport along microtubule
positive regulation of cellular component organization
cellular response to stress
response to cold
regulation of double−strand break repair
positive regulation of organelle organization
double−strand break repair
cellular response to stress
regulation of cell projection organization
tRG (down)
mitotic spindle organization
microtubule cytoskeleton organization involved in mitosis
mitotic nuclear division
spindle organization
organelle fission
regulation of chromosome organization
regulation of ubiquitin−protein transferase activity
regulation of transferase activity
regulation of cellular response to stress
regulation of cyclin−dependent protein kinase activity
regulation of signal transduction by p53 class mediator
regulation of response to stress
negative regulation of Notch signaling pathway
positive regulation of signal transduction by p53 class mediator
regulation of small GTPase mediated signal transduction
Astroglia (up)
G protein−coupled receptor signaling pathway
regulation of trans−synaptic signaling
regulation of intracellular signal transduction
adenylate cyclase−modulating G protein−coupled receptor signaling pathway
cell−cell signaling
G protein−coupled receptor signaling pathway
synaptic signaling
neuron development
synaptic signaling
neurogenesis
neuron development
cell morphogenesis
positive regulation of cell junction assembly
synapse organization
cellular component morphogenesis
synapse assembly
regulation of membrane potential
cell junction organization
cell part morphogenesis
positive regulation of synapse assembly
regulation of membrane potential
synapse organization
regulation of postsynaptic membrane potential
regulation of synaptic structure of activity

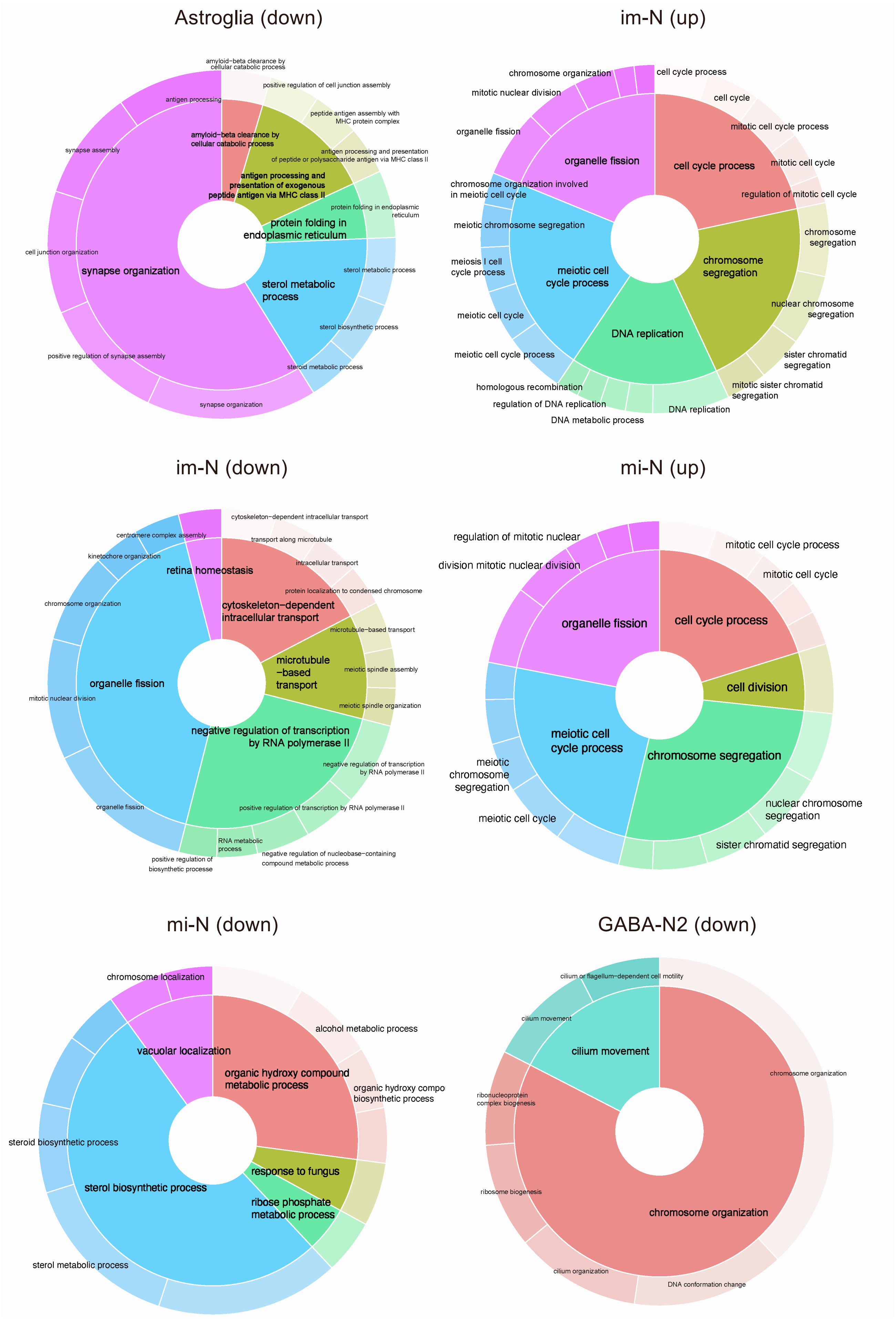

Astroglia (down)
amyloid−beta clearance by cellular catabolic process
positive regulation of cell junction assembly
peptide antigen assembly with MHC protein complex
antigen processing and presentation of peptide or polysaccharide antigen via MHC class II
antigen processing
synapse assembly
amyloid−beta clearance by cellular catabolic process
antigen processing and presentation of exogenous peptide antigen via MHC class II
protein folding in endoplasmic reticulum
protein folding in endoplasmic reticulum
cell junction organization
synapse organization
sterol metabolic process
sterol metabolic process
sterol biosynthetic process
positive regulation of synapse assembly
steroid metabolic process
synapse organization
im-N (up)
chromosome organization
cell cycle process
mitotic nuclear division
cell cycle
organelle fission
mitotic cell cycle process
organelle fission
cell cycle process
mitotic cell cycle
chromosome organization involved in meiotic cell cycle
regulation of mitotic cell cycle
meiotic chromosome segregation
chromosome segregation
chromosome segregation
meiosis I cell cycle process
meiotic cell cycle process
meiotic cell cycle
nuclear chromosome segregation
DNA replication
meiotic cell cycle process
sister chromatid segregation
homologous recombination
mitotic sister chromatid segregation
regulation of DNA replication
DNA replication
DNA metabolic process
im-N (down)
cytoskeleton−dependent intracellular transport
centromere complex assembly
transport along microtubule
kinetochore organization
retina homeostasis
intracellular transport
protein localization to condensed chromosome
chromosome organization
cytoskeleton−dependent intracellular transport
microtubule−based transport
microtubule −based transport
meiotic spindle assembly
organelle fission
mitotic nuclear division
meiotic spindle organization
negative regulation of transcription by RNA polymerase II
negative regulation of transcription by RNA polymerase II
organelle fission
positive regulation of transcription by RNA polymerase II
RNA metabolic process
positive regulation of biosynthetic processe
negative regulation of nucleobase−containing compound metabolic process
mi-N (up)
regulation of mitotic nuclear
mitotic cell cycle process
division mitotic nuclear division
mitotic cell cycle
organelle fission
cell cycle process
cell division
meiotic cell cycle process
chromosome segregation
meiotic chromosome segregation
nuclear chromosome segregation
meiotic cell cycle
sister chromatid segregation
mi-N (down)
chromosome localization
alcohol metabolic process
vacuolar localization
organic hydroxy compound metabolic process
organic hydroxy compo biosynthetic process
steroid biosynthetic process
response to fungus
sterol biosynthetic process
ribose phosphate metabolic process
sterol metabolic process
GABA-N2 (down)
cilium or flagellum−dependent cell motility
cilium movement
cilium movement
chromosome organization
ribonucleoprotein complex biogenesis
ribosome biogenesis
chromosome organization
cilium organization
DNA conformation change

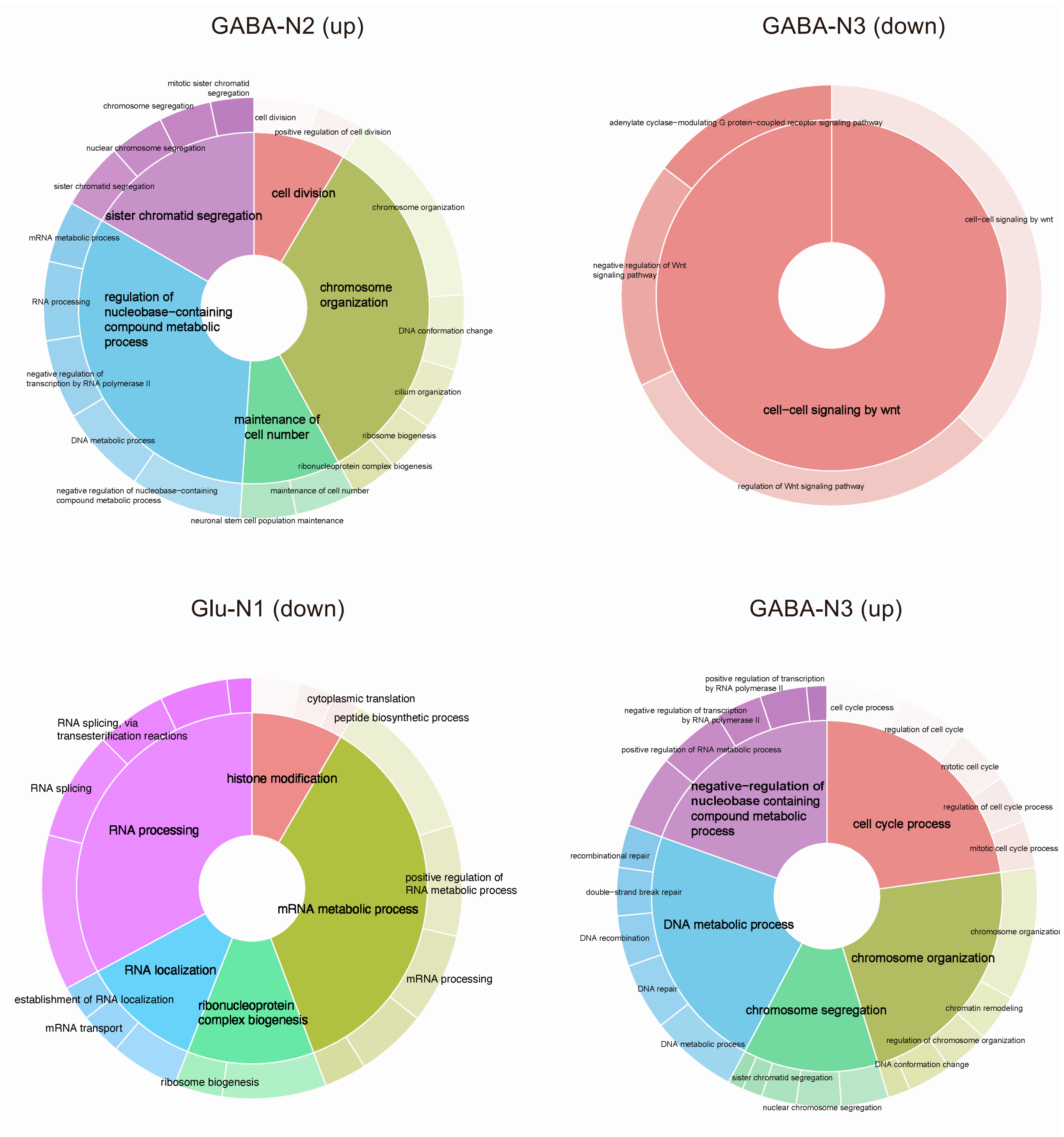

Figure S9: Gene set enrichment analysis (GSEA) and functional enrichment of DEGs in SMS hiPSCs-derived cortical organoids at day 75 differentiation

Hierarchical pie charts showing GO biological processes enriched among up- or down-regulated genes in different SMS cortical organoid cell clusters.

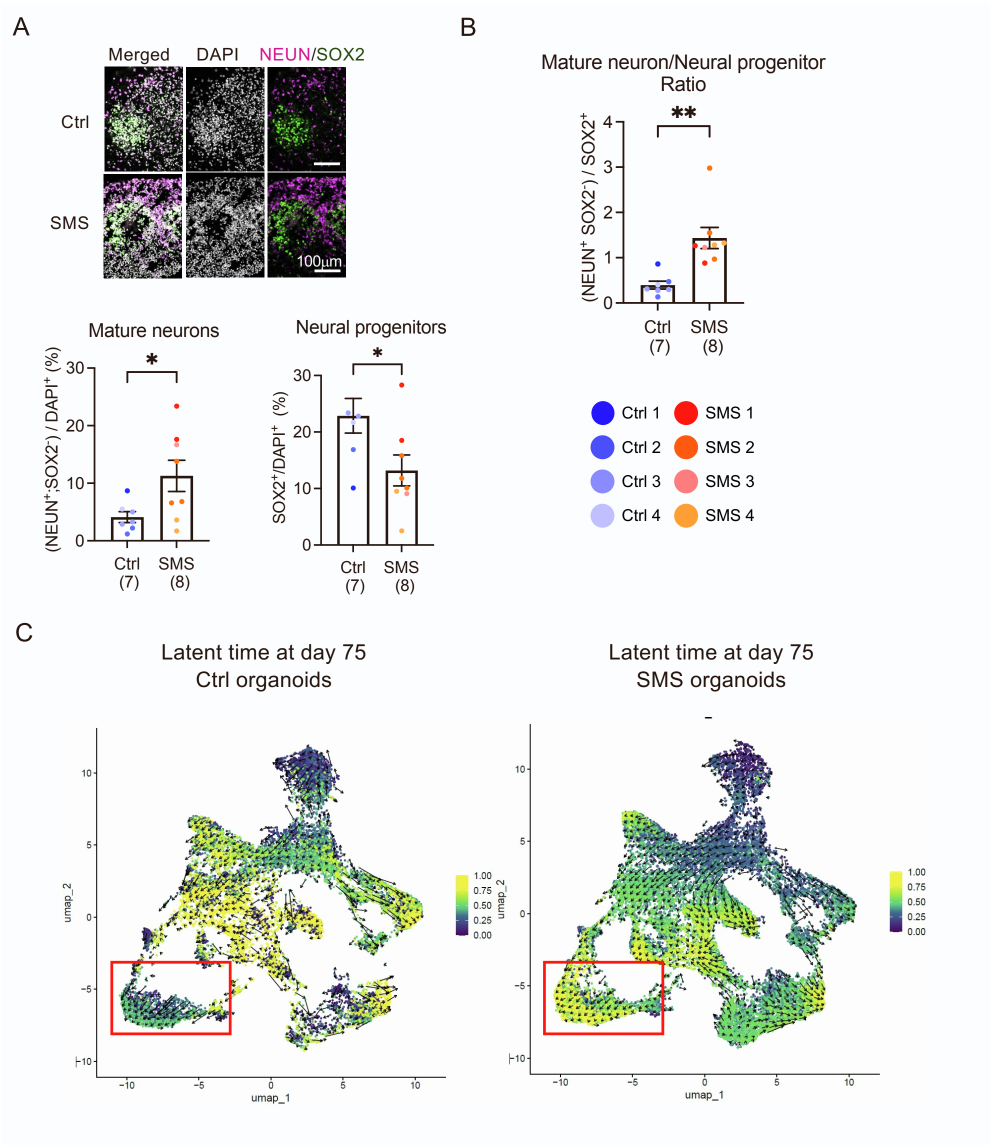

Figure S10: SMS hiPSCs-derived cortical organoids showed an accelerated neuronal maturation

(A-B) Top: Representative images of day 75 Ctrl and SMS organoids with SOX2 and NEUN co-staining. Bottom: SMS organoids showed an increased $NEUN^+$ $SOX2^-$ mature neuronal population, a decreased $SOX2^+$ neural progenitor population **(A)**, and an increased NEUN-to-SOX2 ratio **(B)** at day 75 of differentiation. Data were analyzed from 4um-thick confocal images of >6000 cells from each line (Ctrl n= 7 organoids, 16 images, 63557 cells; SMS n=8 organoids, 12 images, 40744 cells). *p<0.05, **p<0.01, *p*-values by Welch's t-tests.

(C) Latent time calculated using the snRNA-seq dataset revealed that SMS Glu-N1 and Glu-N2 populations (red rectangles) undergo accelerated neuronal maturation. By contrast, Ctrl cells are more biased towards the migrating neuron and immature neuronal trajectories than SMS cells. Heatmap: a latent time value of 0 represents the start of a cell's differentiation process, while a latent time value of 1 represents a terminal or final state.

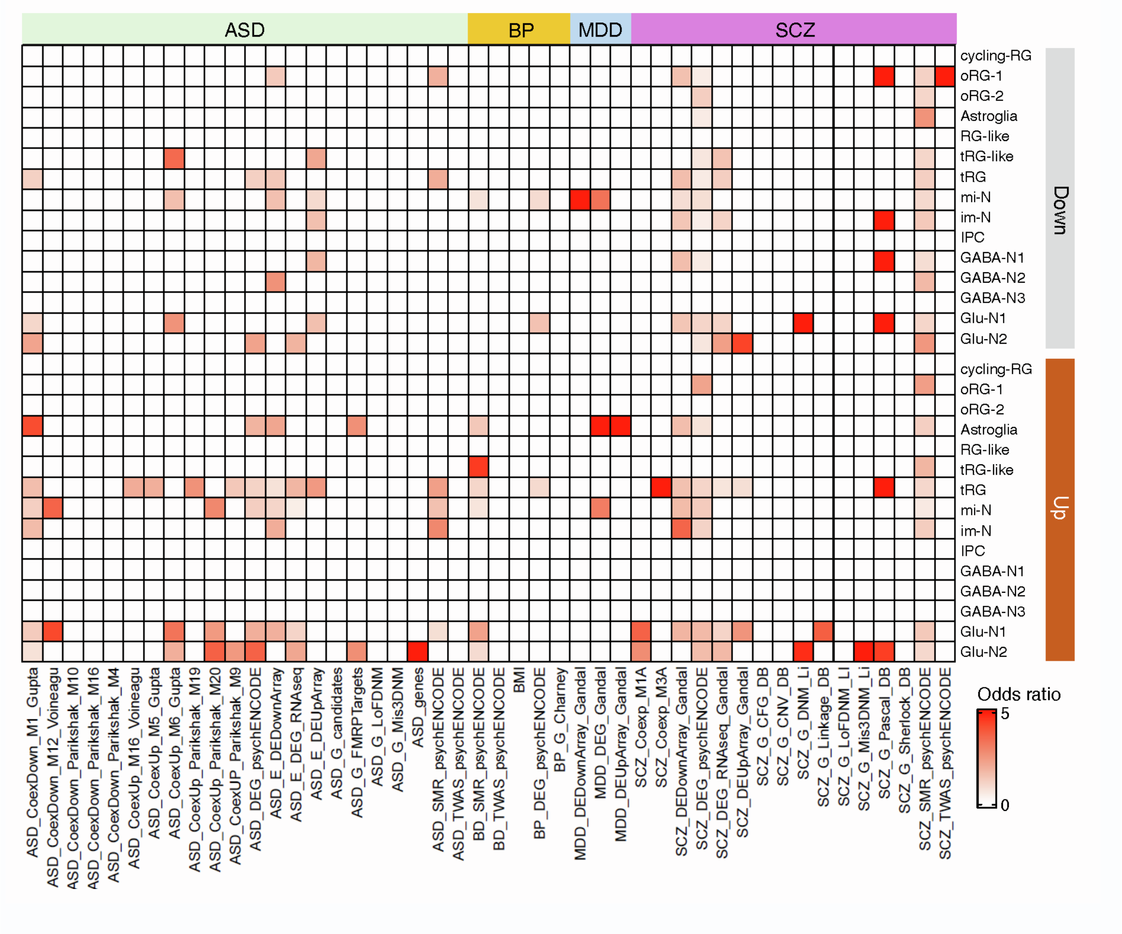

Figure S11: ASD and neuropsychiatric gene enrichment analysis using SMS DEGs (FDR<0.1) Heatmap showing over-representation of autism spectrum disorder (ASD), bipolar disorder (BD), major depressive disorder (MDD) and schizophrenia (SCZ)-related genes in different cell cluster DEGs (FDR<0.1). The color of the box shows the odds ratio for enrichment. The odds ratios were calculated by Fisher's exact test.

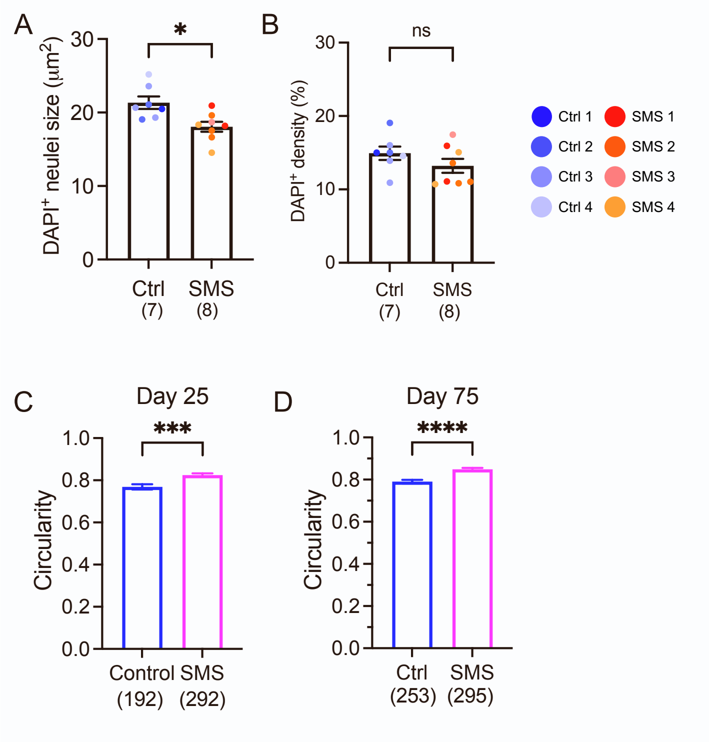

Figure S12: Cellular characterization of SMS cortical organoids

(A-B) SMS organoids exhibited a significantly decreased DAPI$^+$ nuclear size **(A)**, without measurable changes in DAPI$^+$ nuclear density **(B)** at day 75 of differentiation. Data were analyzed from 4μm-thick, confocal images of >40,000 cells from each genotype (Ctrl n= 7 orgnaoids; SMS n=8 orgnaoids). DAPI$^+$ density was measured by the percentage of DAPI$^+$ area within the orgnoid's surface area (excluding the ventricles).

(C-D) Measuring the circularity found that SMS organoids showed a small but significant increase in the C value at both day 25 **(C)** and day 75 **(D)** of differentiation when compared to age-matched Ctrl organoids. Shown are mean±SEM. The number of organoids in each group are listed below.

ns, not statistically different; *p<0.05, ***p<0.001, ****p<0.001, *p*-values by Welch's t-tests.

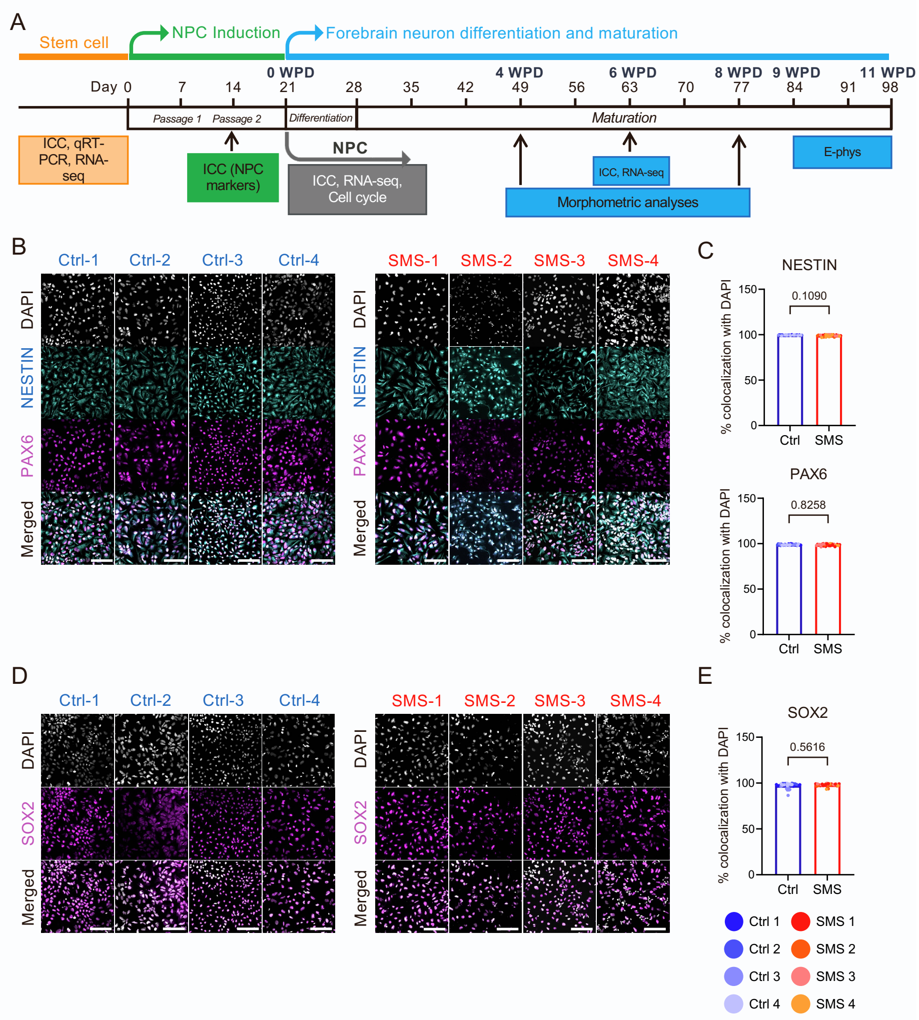

Figure S13: Characterization of Ctrl and SMS hiPSCs-derived neural progenitor cells (NPCs)

(A) Schematic diagram of neural induction from hiPSC into NPC using a monolayer protocol then differentiation into cortical neurons, denoting timepoints of experiments. WPD: weeks post-neural differentiation.

(B) Representative images of Ctrl and SMS hiPSC-derived NPCs stained with NPC markers NESTIN (cyan) and PAX6 (magenta). DAPI-labeled nuclei are in grey. Scale bars: 100µm.

(C) Quantification of NESTIN (top) and PAX6 (bottom) in Ctrl and SMS hiPSC-derived NPCs. n = 32 images per genotype with 8 images per cell line, each dot represents 1 image coloured based on the cell line. NESTIN: U=392.5, p=0.1090; PAX6: U=495, p=0.8258.

(D) Representative images of Ctrl and SMS hiPSC-derived NPCs stained with the NPC marker SOX2 (magenta). DAPI-labeled nuclei are in grey. Scale bars: 100µm.

(E) Quantification of SOX2 in Ctrl and SMS hiPSC-derived NPCs. n = 32 images per genotype with 8 images per cell line, each dot represents 1 image coloured based on the cell line. U=468, p=0.5616.

Data presented as means ± S.E.M.; (**C** and **E**) U- and p-values by two-tailed Mann-Whitney tests.

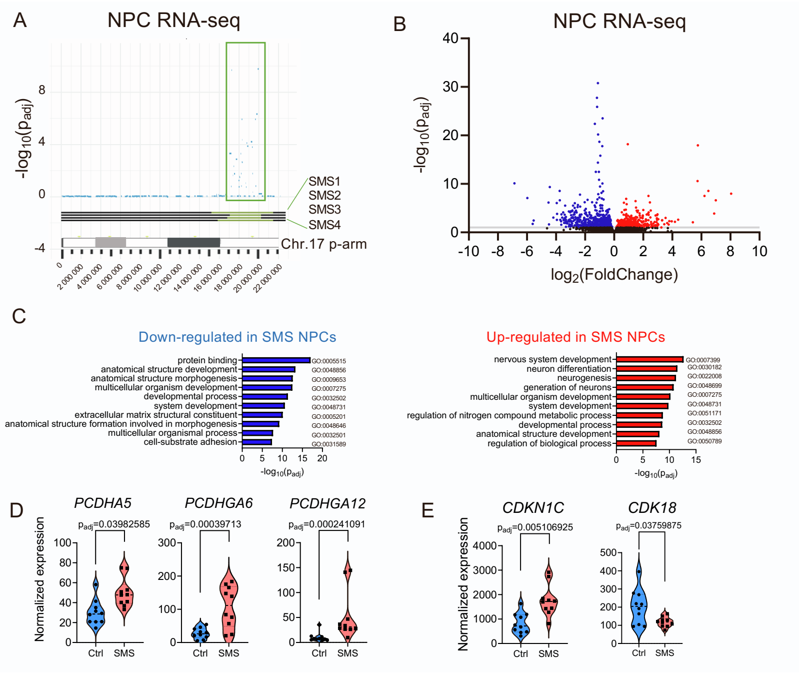

Figure S14: RNA-seq data obtained from Ctrl and SMS hiPSCs-derived neural progenitor cells (NPCs)

(A) A Manhattan plot displaying the genomic landscape of significantly downregulated genes on the chromosome 17 p-arm in SMS NPCs based on -$\log_{10}$ transformed p_{adj} derived from a one-sided Wald test. The x-axis represents the genomic position, and the y-axis corresponds to the significance level of differential expression. The blue dots represent individual genes, with their positions on the x-axis representing their location on chromosome 17p and their heights indicating the significance of their differential expressions. The dark horizontal lines indicate chromosomes, with the green segments corresponding to the regions subjected to del(17)p11.2 in SMS NPCs.

(B) Volcano plots showing the global transcriptomic changes when comparing SMS with Ctrl NPCs. Each dot represents a gene. The $\log_2$ fold change of each gene is represented on the x-axis and the -$\log_{10}$ of its p_{adj} is on the y-axis. Up-regulated genes in SMS NPCs with a p_{adj} less than 0.1 are indicated by red dots. Down-regulated genes in SMS NPCs with a p_{adj} less than 0.1 are indicated by blue dots.

(C) Gene ontology analyses of genes involved in anatomical development and protein binding that were down-regulated in SMS NPCs. By contrast, nervous system developmental and differentiation genes were up-regulated in SMS NPCs. The top 10 GO terms for differentially up-regulated genes (in red), down-regulated genes (in blue), and their respective -$\log_{10}(p_{adj})$ are shown.

(D) Violin plots illustrating down-regulated protocadherin genes in SMS NPCs that are similarly mis-regulated in SMS hiPSCs. Each dot represents a sample (2-3 samples/cell line, n = 20). p_{adj} by Wald test.

(E) Violin plots illustrating selective mis-regulated cell cycle genes in SMS NPCs. Each dot represents a sample (2-3 samples/cell line, n = 20). p_{adj} by Wald test.

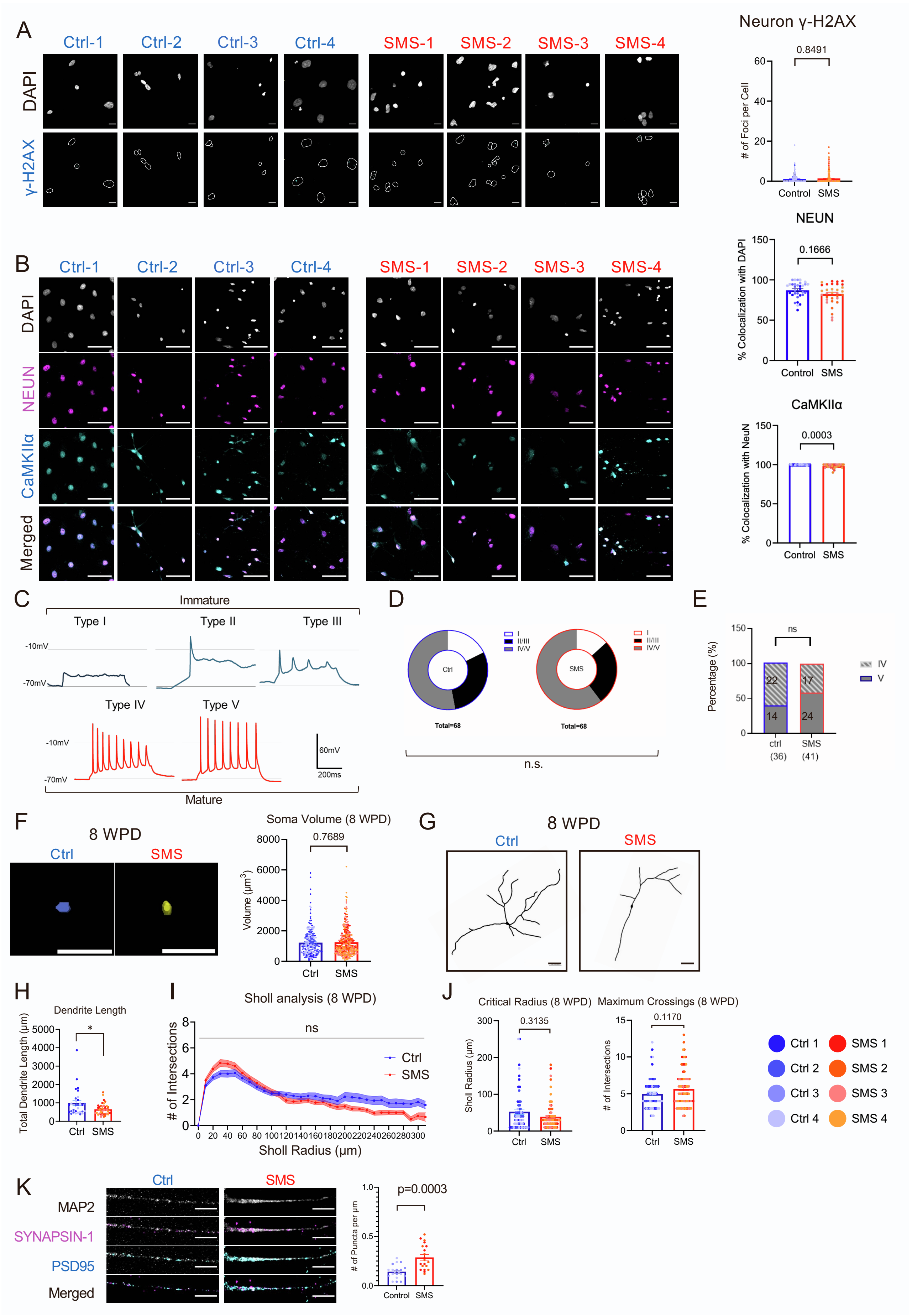

A
Ctrl-1
Ctrl-2
Ctrl-3
Ctrl-4
SMS-1
SMS-2
SMS-3
SMS-4
DAPI
γ-H2AX
Neuron γ-H2AX
0.8491
# of Foci per Cell
Control
SMS
B
NEUN
CaMKIIα
Merged
0.1666
% Colocalization with DAPI
0.0003
% Colocalization with NeuN
C
Immature
Type I
Type II
Type III
Type IV
Type V
-10mV
-70mV
60mV
200ms
Mature
D
Ctrl
SMS
I
II/III
IV/V
Total=68
n.s.
E
ns
Percentage (%)
IV
V
22
14
17
24
ctrl
(36)
(41)
F
8 WPD
Soma Volume (8 WPD)
0.7689
Volume (μm³)
G
H
Dendrite Length
Total Dendrite Length (μm)
I
Sholl analysis (8 WPD)
# of Intersections
Sholl Radius (μm)
J
Critical Radius (8 WPD)
0.3135
Sholl Radius (μm)
Maximum Crossings (8 WPD)
0.1170
# of Intersections
Ctrl 1
Ctrl 2
Ctrl 3
Ctrl 4
SMS 1
SMS 2
SMS 3
SMS 4
K
MAP2
SYNAPSIN-1
PSD95
p=0.0003
# of Puncta per μm

Figure S15: Morphological and functional characterization of Ctrl and SMS hiPSC-derived cortical neurons

(A) Left: Representative images of Ctrl and SMS hiPSC-derived neurons stained with a double-strand break DNA damage marker γ-H2AX (cyan). Bottom: outlined nuclei for γ-H2AX quantification. Scale bars: 10µm. Right: Quantification showing that a similar number of γ-H2AX foci in Ctrl and SMS neurons. Each dot represents one neuron. U=31490, p=0.8491, two-tailed Mann-Whitney test.

(B) Left: Representative images of Ctrl and SMS hiPSC-derived neurons stained with a mature neuronal marker NEUN (magenta) and an excitatory cortical neuronal marker CaMKIIα (cyan). DAPI-labeled nuclei are in grey. Scale bars: 50µm. Upper right: Quantification showing that a similar number of $DAPI^+$ Ctrl and SMS cells express NEUN. Lower right: Quantification showing that 99.8% of Ctrl $NEUN^+$ neurons and 98.2% of SMS $NEUN^+$ neurons express CaMKIIα. Each dot represents one neuron. NEUN: U=408.5, p=0.1666; CaMKIIα: U=289.5, p=0.0003. U- and p-values by two-tailed Mann-Whitney tests.

(C) Representative traces of hiPSC-derived cortical neuronal action potential (AP) firing (I_{clamp} mode). A small current was injected to maintain the resting membrane potential around –70mV. Next, APs were evoked by current steps (increment, 10pA, 500ms, from –110 pA to the deplorization step causing membrane potential rises and APs to fail). A successful AP firing is defined as a fast depolarization overshoot greater than –10 mV. Immature neurons include Types I-III: Type I, failed to fire AP; Type II: single AP firing; Type III, single AP firing followed by unsuccessful events. Mature cortical neurons include Types IV and V: Type IV, repetitive AP firing with decaying overshoot values; Type V, repetitive and uniform AP firing.

(D) Pie charts showing a similar proportion of hiPSC-derived Ctrl (blue border) and SMS (red border) cortical neurons in each AP firing category. n.s., not significantly different, *Chi-square*=0.2506, df=2, p=0.8822, Chi-squared analysis.

(E) A similar percentage of Ctrl (blue border) and SMS (red border) neurons in type IV (grey stripes) and type V (grey bar) categories. n.s., not significantly different, chi-squared analysis. *Chi-square*=0.5951, df=1, p=0.4405, Chi-squared analysis.

(F) Left: Representative 8 WPD soma 3D images of Ctrl and SMS cortical neurons transduced with myrGFP lentivirus. Scale bar: 50µm. Right: Bar plot showing a similar soma volume between Ctrl and SMS neurons at 8 WPD. n = 225-300 cells per genotype with 75 cells per cell line. Each dot represents one soma coloured based on the cell line. t=0.2939, df=523, p=0.7689, unpaired t-test.

(G) Representative 3D reconstituted neuron images of myrGFP-transduced Ctrl and SMS hiPSC-derived neurons at 8 WPD. Scale bar: 50µm.

(H) Quantification showing $myrGFP^+$ Ctrl hiPSC-derived neurons have longer dendrites compared to SMS neurons at 8WPD. Ctrl group has 30 neurons and SMS group has 40 neurons, with 10 neurons per cell line. Each dot represents one neuron. U=393, p=0.0136, two-tailed Mann-Whitney test.

(I) Sholl analysis of Ctrl and SMS hiPSC-derived neurons at 8 WPD. n = 60 Ctrl neurons and 80 SMS neurons. Presented as differences between means (line) ± S.E.M (shade). p-values >0.05 for all bins, calculated by two-sided multiple t-tests.

(J) Quantification of Sholl critical radius (left) and maximum crossings (right) of Ctrl and SMS hiPSC-derived neurons at 8 WPD. Ctrl group has 60 neurons and SMS group has 80 neurons, with 20 neurons per cell line. Each point represents one neuron. Critical radius: U=2163, p=0.3135; Max crossings: U=2033, p=0.1170. U- and p-values by two-tailed Mann-Whitney tests.

(K) Quantification of excitatory synapse density (presynaptic SYNAPSIN-1$^+$ and postsynaptic PSD95$^+$) in Ctrl (Ctrl-3 and Ctrl-4) and SMS (SMS-1 and SMS-2) cortical neurons at 6 WPD. Left: Representative images of Ctrl and SMS neurons stained with MAP2 (grey), SYANPSIN-1 (magenta), and PSD95 (cyan). Scale bar: 10µm. Right: Quantification of the number of puncta (SYANPSIN-1$^+$ and PSD95$^+$) per µm. Each dot represents one 50µm segment, n=20 segments per genotype with 10 segments per cell line. U=62, p=0.0003, two-tailed Mann-Whitney test.

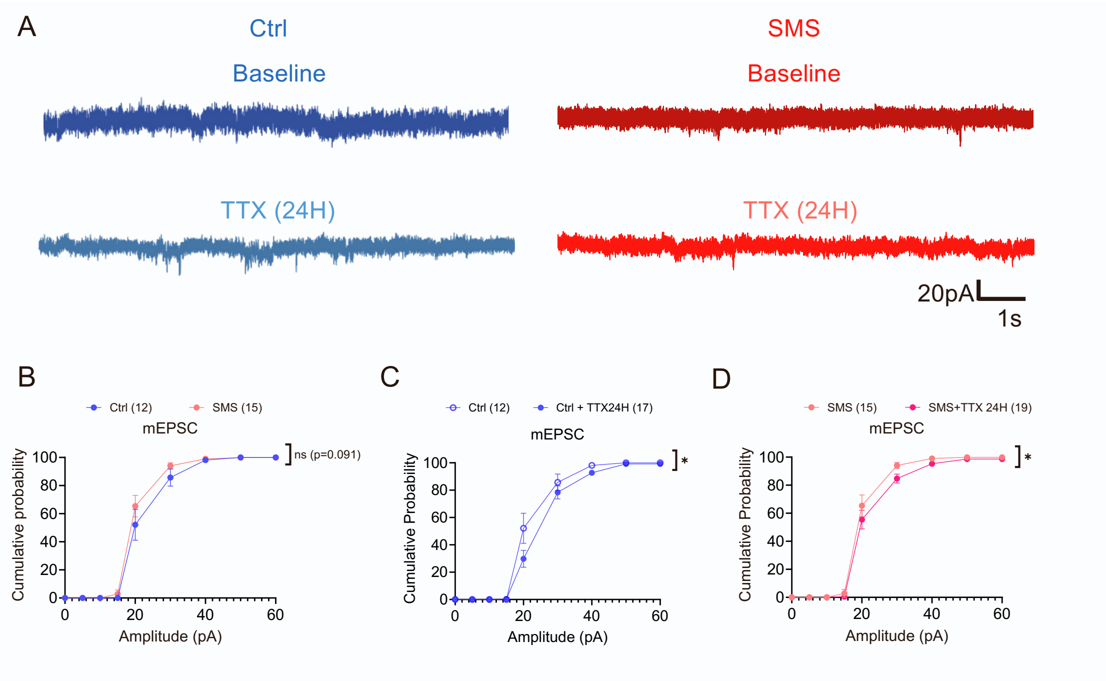

Figure S16: Normal glutamatergic synaptic transmission and up-scaling in Ctrl and SMS hiPSCs-derived 2D cortical neurons

(A) Representative traces of mEPSCs in Ctrl and SMS hiPSC-derived cortical neurons (holding potential at −70 mV). Top: baseline condition with acute TTX treatment to block AP; Bottom: after 24 hours of TTX treatment to silence neuronal firing and induce synaptic upscaling.

(B-D) Distribution of mEPSC amplitudes in Ctrl vs SMS neurons at baseline (genotype=$F(1,325)=2.873$, $p=0.091$; amplitude=$F(12,325)=510.6$, $p<0.0001$) **(B)**. TTX treatment for 24 hours (TTX 24H) in Ctrl neurons induced a right shift in the cumulative probability plot (treatment=$F(1,351)=6.450$, $p=0.0115$; amplitude=$F(12,351)=549.0$, $p<0.0001$) **(C)**, indicating a normal synaptic upscaling. Similarly, TTX treatment for 24 hours in SMS neurons induced synaptic upscaling (treatment=$F(1,416)=6.343$, $p=0.0122$; amplitude=$F(12,416)=817.0$, $p<0.0001$) **(D)**. F(DFn, DFd) and *p*-values were calculated by two-way ANOVA with post hoc Šídák multiple comparisons.

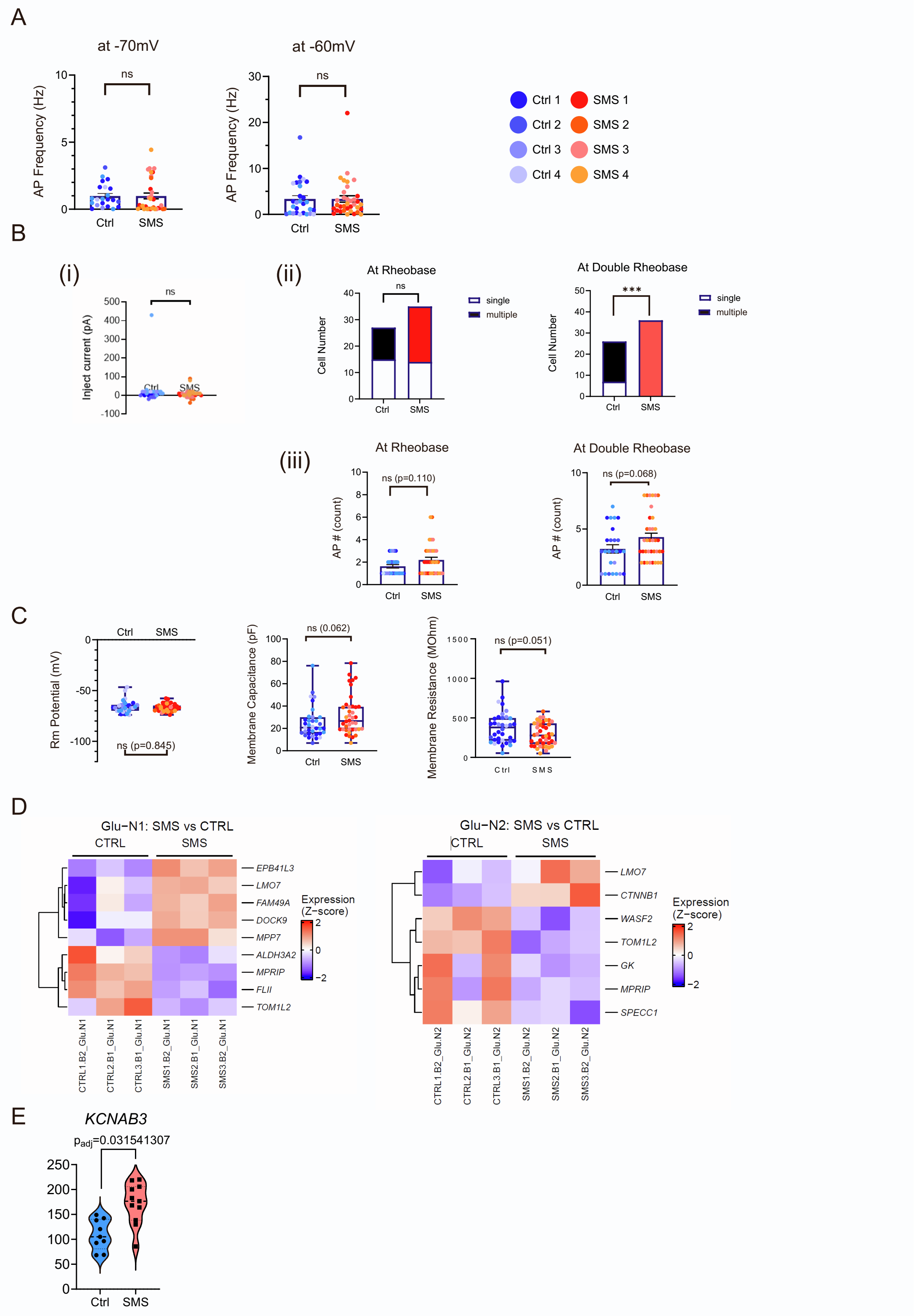
A
at -70mV
at -60mV
AP Frequency (Hz)
ns
Ctrl
SMS
Ctrl 1
Ctrl 2
Ctrl 3
Ctrl 4
SMS 1
SMS 2
SMS 3
SMS 4
B
(i)
Inject current (pA)
(ii)
At Rheobase
At Double Rheobase
Cell Number
single
multiple
***
(iii)
AP # (count)
ns (p=0.110)
ns (p=0.068)
C
Rm Potential (mV)
ns (p=0.845)
Membrane Capacitance (pF)
ns (0.062)
Membrane Resistance (MOhm)
ns (p=0.051)
D
Glu−N1: SMS vs CTRL
Glu−N2: SMS vs CTRL
CTRL
SMS
EPB41L3
LMO7
FAM49A
DOCK9
MPP7
ALDH3A2
MPRIP
FLII
TOM1L2
CTNNB1
WASF2
GK
SPECC1
Expression
(Z−score)
CTRL1.B2_Glu.N1
CTRL2.B1_Glu.N1
CTRL3.B1_Glu.N1
SMS1.B2_Glu.N1
SMS2.B1_Glu.N1
SMS3.B2_Glu.N1
CTRL1.B2_Glu.N2
CTRL2.B1_Glu.N2
CTRL3.B1_Glu.N2
SMS1.B2_Glu.N2
SMS2.B1_Glu.N2
SMS3.B2_Glu.N2
E
KCNAB3
p_adj=0.031541307

Figure S17: Intrinsic excitability of Ctrl and SMS hiPSCs-derived 2D cortical neurons

(A) Spontaneous AP firing frequency at a holding potential of -70mV (left) and -60mV (right). The silenced cortical neurons were excluded. No significant differences were found between Ctrl and SMS cortical neurons. -70mV: U=306, p=0.3318; -60 mV: U=468, p=0.7650; two-tailed Mann-Whitney tests.

(B) At I_{clamp}, (i) the injected currents in Ctrl and SMS neurons to evoke AP at the rheobase were similar, U=402.5, p=0.235, two-tailed Mann-Whitney test; (ii) There were similar numbers of single and multiple firing Ctrl and SMS neuron populations at the rheobase (*chi-cauqre*=0.922, *df*=1, p=0.3368, Chi-squared analysis) and more SMS neurons fired multiple APs at double rheobase (*chi-cauqre*=10.93, *df*=1, p=0.0009, Chi-squared analysis) (iii) The AP numbers were similar between Ctrl and SMS cortical neurons. Rheobase: U=367, p=0.110; Double Rheobase: U=342, p=0.0681, *U*- and *p*-values by two-tailed Mann-Whitney tests.

(C) The membrane properties of Ctrl and SMS cortical neurons. There were no significant differences in resting membrane potential, membrane capacitance, and membrane resistance. Rm: U=521, p=0.845; membrane capacitance: U= 524, p=0.062; membrane resistance: U= 561.5, p=0.051; *U*- and *p*-values by two-tailed Mann-Whitney tests.

(**D**) Heatmaps showing that Glu-N1 and Glu-N2 excitatory neurons from SMS organoids overexpressed several synaptic genes. By contrast, synaptic genes down-regulated in SMS excitatory neurons belong to del(17)p11.2.

(E) Violin plots illustrating that bulk RNA-seq detected up-regulated *KCNAB3* in SMS neurons when compared to Ctrls. Each dot represents a sample. p_{adj} by Wald test.

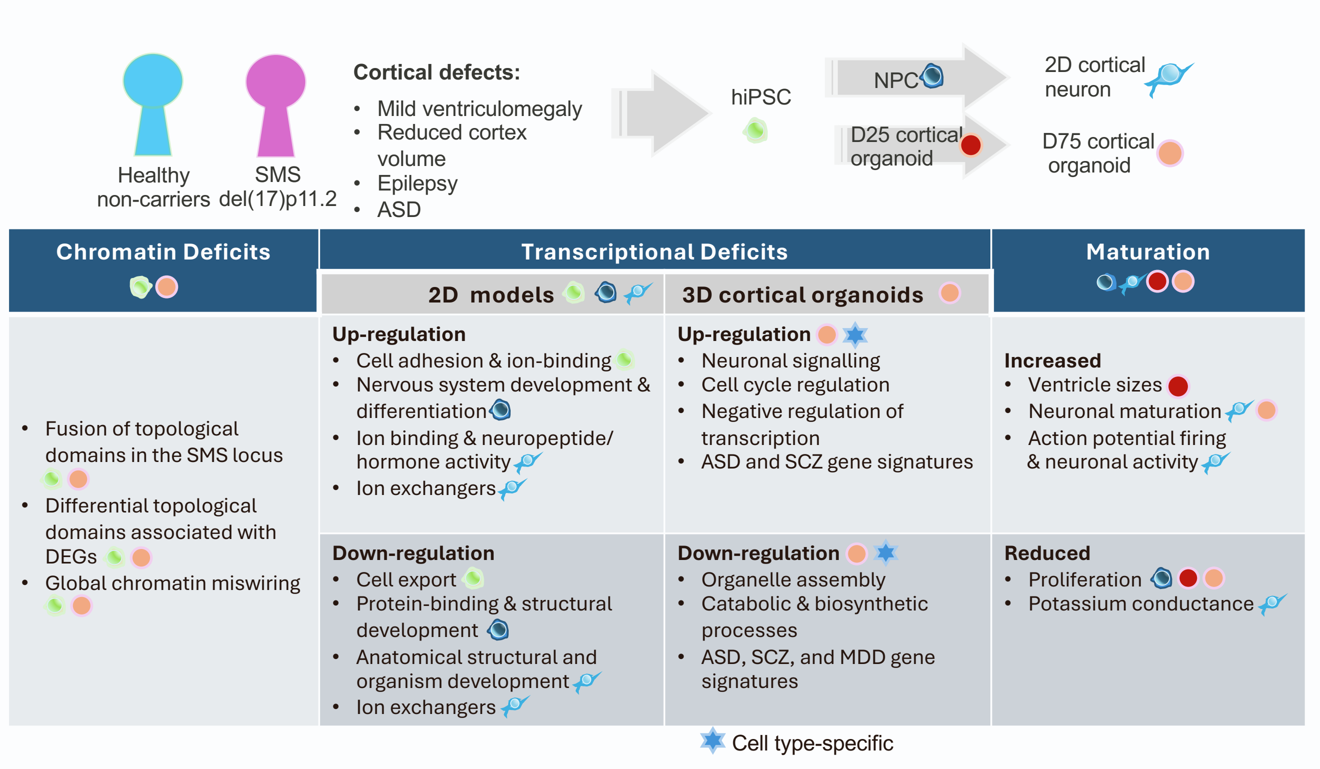

Figure S18: Summary of the findings

Table S1: Information on hiPSC lines

Name	Coriell number	Disease	Genomic deletions	Biopsy Sources	Tissue types	Sex	Age
Ctrl-1	GM23279	Apparently healthy	None	Skin	hiPSC	Female	36 years
Ctrl -2	GM23720	Apparently healthy	None	B-Lymphocyte	hiPSC	Female	22 years
Ctrl -3	GM23716	Apparently healthy	None	Skin	hiPSC	Female	37 years
Ctrl -4	AG27875	Apparently healthy	None	Skin	hiPSC	Male	37 years
SMS-1	NA	SMS	Chr17 del:15,175,307-21,519,100	Skin	hiPSC (reprogrammed from GM25367)	Female	21 years
SMS-2	GM27918	SMS	Chr17 del:16,881,282-20,121,029	Skin	hiPSC	Female	20 years
SMS-3	NA	SMS	Chr17 del:16,769,800-20,126,017	Skin	hiPSC (reprogrammed from GM25371)	Female	12 years
SMS-4	NA	SMS	Chr17 del:15,777,172-20,554,628	Skin	hiPSC (reprogrammed from GM24311)	Female	23 years

Table S2: Clinical features of SMS patients

SMS patients	Clinical features
SMS-1	Diagnosed at age 7 years via FISH test; brachycephaly; midface retrusion (hypoplasia); broad nasal bridge; down-turned upper lip; prognathism; synophrys; defective vision; strabismus; hearing defect; decreased pain sensitivity; dental abnormalities; hoarse voice; pes planus; scoliosis; syndactyly; tetralogy of Fallot; constipation; short stature; overweight/obesity; high total cholesterol; self-injurious behavior
SMS-2	Diagnosed at age 2 years via FISH test; breech pregnancy; laryngomalacia; defective vision; hearing defect; decreased pain sensitivity; coloboma; midface retrusion (hypoplasia); dental abnormalities; down-turned upper lip; scoliosis; cardiac issues include: ASD, VSD, pulmonary stenosis; intellectual disability (52 IQ/DQ); self-injurious behavior
SMS-3	Diagnosed at 9 years of age; brachycephaly; large forehead; down-turned upper lip; hearing defect; hypotonia; decreased pain sensitivity; abnormal gait; brachydactyly; pes planus; scoliosis; constipation; short stature; speech delay; overweight/obese; decreased tolerance to exercise; intellectual disability; sleep disturbance; self-hugging; immunologic abnormalities; ear infections
SMS-4	Diagnosed at 14 years; dysmorphic facial features; hypotelorism; dental abnormalities; hoarse voice; dry skin; brachydactyly; scoliosis; short stature; hypotonia; obesity; defective vision, strabismus; hearing defect; ataxia, decreased pain sensitivity; constipation; fine motor delay; gross motor delay; speech delay; intellectual disability; ADHD; oppositional defiant disorder; obsessive compulsive disorder; sleep disturbance; repetitive behaviors; self-injurious behaviors; anxiety disorder; disruptive behavior disorder; high total cholesterol

Table S3: Hi-C contacts per chromosome for each sample (Excel file provided)

Table S4: qRT-PCR primer sequences

Gene	Forward	Reverse
RAI1	CCTCAGCATTCCCAGTCCTTC	CTGTGCAACTCTTATAGGAGTGG
GAPDH	AAGGTGAAGGTCGGAGTCAA	AATGAAGGGGTCATTGATGG
PCDHA3	CGTGTACTTGATCGTCGCCAT	CAAGGAGGAAGGCTAGGGCTA
PCDHA9	CGCGGTGTCTAGCCTGTTG	CCCGTTCGCTCTGTAGATCC

Table S5: Number of samples for each line used in each experiment (Excel file provided)
